# Twenty years of therapeutic development in tauopathy mouse models: a scoping review

**DOI:** 10.1002/alz.70578

**Published:** 2025-08-18

**Authors:** Vanessa F. Langness, Danielle A. Simmons, Tyne L. M. McHugh, Robert R. Butler, James Zhou, Harry Liu, Tao Yang, Lisa M. Ellerby, Frank M. Longo

**Affiliations:** ^1^ Department of Neurology and Neurological Sciences Stanford University School of Medicine Palo Alto California USA; ^2^ Buck Institute for Research on Aging Novato California USA; ^3^ Leonard Davis School of Gerontology University of Southern California Los Angeles California USA; ^4^ Wu Tsai Neuroscience Institute Cogen Facility Stanford University Stanford California USA

**Keywords:** Alzheimer's disease (AD), frontotemporal lobar degeneration (FTLD), MAPT mouse models, scoping review, synapse, tauopathy, therapeutic strategies, treatment

## Abstract

Tauopathies are neurodegenerative diseases characterized by pathological tau protein inclusions and dementia. Tauopathy mouse models with *MAPT* mutations replicate tau‐related pathologies and are widely used for therapeutic research. This scoping review examines 409 treatment evaluations in *MAPT* mouse models. We identify trends in therapeutic strategies and frequently used mouse models, treatment routes, and endpoints. We also document treatment effects and when treatment is initiated relative to tau pathology emergence. Many treatments produced positive effects in multiple *MAPT* mouse models across many endpoints but showed limited success in clinical trials. Potential barriers to mouse‐to‐human translation include differences between mouse and human studies in the timing of treatment initiation relative to tau pathology onset, predominant testing of a limited number of endpoints, lack of translatable treatment response biomarkers, and the limited ability of individual mouse models to represent the diversity of tauopathies. Addressing these obstacles could improve mouse‐to‐human translation for tauopathy therapeutics.

**Highlights:**

- Two decades of therapeutic research in tauopathy mouse models were reviewed.
- Treatments often began before or at tau pathology onset in tauopathy mouse models.
- Key endpoints (e.g., cognition and synaptic degeneration) were underassessed.
- Well‐characterized preclinical treatments often had limited success in humans.
- Single‐sex mouse studies and a lack of biomarkers hinder clinical translation.

## 1 INTRODUCTION

Tauopathies are a group of over 20 neurodegenerative diseases characterized by inclusions of an insoluble, fibrillar form of the tau protein in the brain and/or spinal cord.^1^, ^2^ These inclusions can be caused by pathological alterations in tau post‐translational modifications (PTMs), primarily hyperphosphorylation, leading to misfolding, self‐aggregation, and fibrillization. Clinical manifestations among tauopathies are varied and include cognitive dysfunction/dementia and behavioral and movement disorders.^3^, ^4^ Tauopathies are accompanied by synaptic dysfunction and loss, which precede neuronal death and brain atrophy and correlate strongly with tau pathology and clinical manifestations.^5^, ^6^, ^7^, ^8^, ^9^, ^10^, ^11^, ^12^, ^13^, ^14^, ^15^, ^16^, ^17^ They typically manifest as adult‐onset neurodegeneration; however, tau pathology also occurs in several neurodevelopmental disorders, including autism, as a result of mutations in genes such as activity‐dependent neuroprotective protein (ADNP) that have downstream effects on tau.^18^, ^19^, ^20^ Tau pathology may have both distinct and overlapping effects in neurodevelopmental disorders and adult‐onset tauopathies.^18^ Alzheimer's disease (AD), the most prevalent tauopathy, is classified as a secondary tauopathy as tau pathology occurs alongside amyloid pathology.^3^, ^21^ Primary tauopathies are driven primarily by tau pathology and marked by frontal and temporal lobe atrophy. They comprise a group of diseases called frontotemporal lobar degeneration with tau inclusions (FTLD‐tau), which includes Pick's disease (PiD), corticobasal degeneration (CBD), progressive supranuclear palsy (PSP), globular glial tauopathy (GGT), and argyrophilic grain disease (AGD).^1^, ^22^, ^23^

**RESEARCH IN CONTEXT**

1. **Systematic review**: We systematically searched PubMed and Embase for preclinical studies that tested treatments in *MAPT* mouse models of tauopathy. We extracted and analyzed detailed information from 409 treatment evaluations across 314 studies spanning over two decades of research, beginning in the year the first *MAPT* mouse model became available.
2. **Interpretation**: We identify therapeutic strategies, frequently studied treatments with broad effects across multiple endpoints, and data‐driven recommendations that may improve the ability of preclinical tauopathy therapeutic studies to predict efficacy in clinical trials.
3. **Future directions**: Translational relevance could be improved by refining model selection, assessing a variety of endpoints (including functional and cellular endpoints), aligning treatment timepoints used in mouse and human studies based on stage of pathology, using clinically relevant administration routes, evaluating sex‐specific treatment effects, and utilizing translatable tau treatment response biomarkers. Continued development of such biomarkers and *MAPT* mouse models will support these efforts.

FTLD‐tau cases are sporadic, but approximately 40% of cases are familial and caused by mutations in the microtubule‐associated protein tau (*MAPT*) gene, suggesting that tau pathology alone can drive neurodegeneration.^1^, ^23^, ^24^ Human brain *MAPT* primarily produces six tau isoforms, which are distinguished by the presence of three or four microtubule‐binding repeats (3R, 4R) and zero, one, or two N‐terminal inserts (0N, 1N, 2N). A seventh isoform, big tau, includes an additional 250 amino acids encoded by the 4a exon and is expressed in specific regions of the central nervous system (CNS).^25^ Tauopathies can be classified as 3R, 4R, or 3R/4R based on the predominant tau isoform(s) present in tau inclusions. Each tauopathy exhibits a unique combination of language, memory, behavioral, and motor deficits, likely influenced by disease‐specific differences in misfolded tau species, predominant tau isoforms, spatiotemporal spread of pathology throughout the brain, and co‐occurring pathologies.^26^, ^27^, ^28^, ^29^ Over 50 *MAPT* mutations are linked to FTLD‐tau and may contribute to pathology through mechanisms shared with sporadic tauopathies, including by promoting tau aggregation and prion‐like activity, reducing microtubule binding and destabilizing microtubules, increasing the ratio of 4R:3R tau, or a combination of these interrelated factors.^23^

Tauopathy‐related degeneration is multifaceted, offering many therapeutic targets that are too numerous to discuss in depth here but were recently reviewed elsewhere.^24^, ^26^, ^30^ Briefly, tau, in its soluble and unfolded state, is enriched in axons, where it binds to and stabilizes microtubules, which support axonal structural integrity and transport as well as neurite outgrowth.^21^, ^24^ Various kinases (e.g., glycogen synthase kinase 3 β [GSK3β], cyclin‐dependent kinase 5, and protein kinase A) and phosphatases (e.g., protein phosphatase 2A [PP2A]) regulate tau phosphorylation at over 80 residues. Hyperphosphorylation of tau reduces its affinity for microtubules, leading to its mislocalization and aggregation into insoluble filaments. In addition to phosphorylation, tau undergoes a variety of other PTMs, including glycosylation, ubiquitination, acetylation, and truncation. Each PTM or combination of PTMs has distinct and interacting effects on tau, including altering its function, localization, aggregation, or clearance through protein degradation pathways.^24^ In tauopathies, PTMs can become imbalanced, contributing to tau's neurotoxicity by causing tau to misfold, accumulate, and propagate in a prion‐like manner, spreading pathology between neurons and across brain regions.^24^ Tau pathology, along with a variety of other damage signals, can trigger immune responses in the brain, which play a complex role in pathogenesis. Neuroinflammation, including microglial and astrocyte activation, initially helps to clear cellular debris, including tau inclusions; however, chronic glial activation can promote tau hyperphosphorylation, misfolding, and neurodegeneration.^24^, ^31^, ^32^ Thus, many tauopathy therapies under development target tau PTM effectors, modulate tau‐related processes (e.g., synthesis, aggregation, or clearance), decrease tau levels directly, or aim to reduce neuroinflammation. Over the past 20 years, a variety of therapeutic strategies have been explored to prevent, reverse, or manage tau‐induced neurodegeneration by targeting these various aspects of tau pathology.

Tauopathy mouse models that express *MAPT* mutations are widely used to investigate potential tauopathy treatments, as they recapitulate key aspects of tau pathology, including tau hyperphosphorylation, tau inclusions (e.g., neurofibrillary tangles [NFTs] or Pick bodies), neuron and synapse loss, and cognitive deficits. Despite reports of promising therapeutic strategies in tauopathy mouse models, no disease‐modifying treatments for tauopathies are available to patients.^33^ To explore this translational gap and to potentially optimize the selection and application of tauopathy preclinical models, we methodically compiled and reviewed 20 years of preclinical tauopathy research, focusing on therapeutic strategies evaluated in *MAPT* mouse models of tauopathy. We examined the most frequently used *MAPT* mouse models, therapeutic approaches, and key treatment endpoints to identify overall patterns of mouse model applications, translational barriers, promising therapeutic strategies, and areas for future research.

## 2 METHODS

### 2.1 Search strategy

A list of 23 *MAPT* mouse models was compiled from the research model database on AlzForum.org by selecting the following search options: “Species: Mouse,” “Gene: *MAPT*,” “Model Type: Single transgene/knock‐in/knock‐out.” ^34^ Electronic databases PubMed and Embase were queried for all available studies published between January 1, 1999 through June 10, 2024, utilizing keywords from each mouse model based on the following keywords and boolean operators: “therapeutic” OR “therapy” OR “treatment” AND “mouse” AND “tau” AND (mouse model name/keyword). Additional studies were identified from internet searches and references from articles obtained from the PubMed/Embase search. January 1, 1999 was used as the start date for the literature search because 1999 is the year that the development of the first single transgene/knock‐in/knock‐out *MAPT* mouse model was published.^35^

### 2.2 Study inclusion and exclusion criteria

**FIGURE 1:**
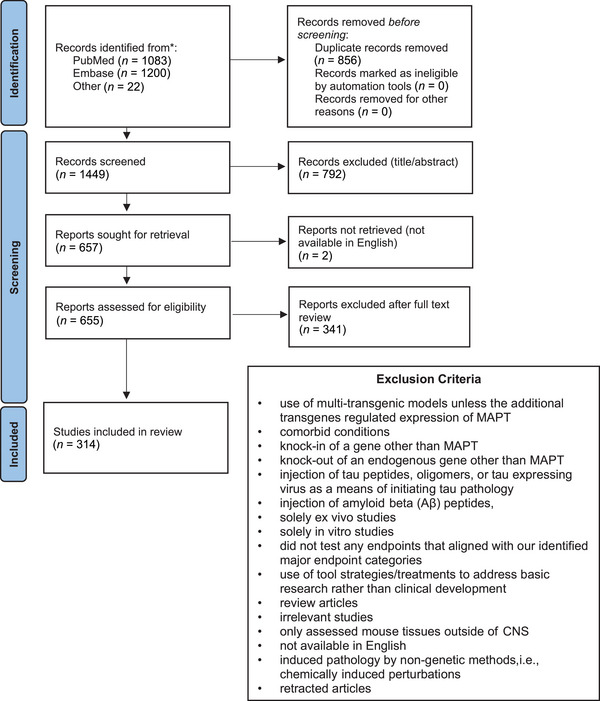
PRISMA flow chart summarizing the study selection process. We identified 2283 records on PubMed and Embase databases using a predefined search string. A total of 22 additional records were identified through other sources, including internet searches, and manually checking reference lists cited in relevant reviews and websites. After removing 856 duplicates and excluding 792 records based on the title or abstract and two due to lack of availability in English, 655 full‐text reports were sought. Following full‐text review, 341 reports were excluded based on our exclusion criteria. A total of 314 studies were included in this review. Key information was extracted and tabulated individually for each treatment tested on each mouse model in each study.

Each of the following five criteria was required for a study to be included in this review: (1) use of at least one *MAPT* mouse model; (2) application of at least one treatment; (3) evaluation of mouse brain and/or spinal cord; (4) presentation of original, peer‐reviewed data; and (5) availability in English. Studies that met one or more of the following 16 criteria were excluded (Figure 1): (1) use of multi‐transgenic mouse models, unless the additional transgenes regulated expression of *MAPT*; (2) comorbid conditions; (3) knock‐in of a gene other than *MAPT*; (4) knock‐out of an endogenous gene other than *MAPT*; (5) injection of tau peptides, oligomers, or tau‐expressing virus as a means of initiating tau pathology; (6) injection of amyloid beta (Aβ) peptides; (7) solely ex vivo studies; (8) solely in vitro studies; (9) no testing of endpoints that aligned with our identified major endpoint categories; (10) use of tool strategies/treatments to address basic research rather than translational therapeutic development; (11) review articles; (12) irrelevant studies (e.g., those that did not evaluate a treatment or those exploring research questions unrelated to tauopathies); (13) only assessed mouse tissues outside of CNS; (14) not available in English; (15) induced pathology by non‐genetic methods, that is, chemically induced perturbations; or (16) retracted articles. Two studies were excluded because they did not test an endpoint that aligned with our determined categories; one only had glucose metabolism and related pathways as an endpoint and the other only evaluated mitochondrial function.^36^, ^37^ Three papers were removed from this review because they were retracted.^38^, ^39^, ^40^

Our literature search, though thorough, may have missed some important studies. The included manuscripts are meant to broadly represent their respective categories, rather than highlight the most seminal or influential works. Our intent is to capture key concepts within major topics, and we apologize in advance for any noteworthy works omitted here. This review was performed in accordance with the Preferred Reporting Items for Systematic Reviews and Meta‐Analysis extension for scoping reviews (PRISMA‐ScR) checklist.^41^, ^42^ A PRISMA flow chart summarizing the study selection process is shown in Figure 1. EndNote 20 was used for reference management.

### 2.3 Data extraction

Three reviewers (H.L., J.Z., T.L.M.) independently extracted data from each of the studies published through December 2020 and entered them into Table S1. This table was updated to include publications through June 10, 2024, by three reviewers (V.F.L., D.A.S., T.L.M.), who also reviewed data extraction for all the previous papers through December 2020 for consistency (Table S1; Supplemental References 1). Discrepancies in data extraction were discussed between V.F.L., D.A.S., and T.L.M. until a consensus was reached. If a consensus could not be reached, F.M.L. acted as arbitrator. Data from all studies were extracted by at least two reviewers. General study characteristics that were extracted and recorded in Table S1 included: first author, year of publication, Digital Object Identifier (DOI), PubMed ID, mouse model, mouse sex and zygosity, treatment agent, treatment synonyms, treatment route, therapeutic strategy, treatment start age, treatment end age, treatment duration, endpoints measured, and efficacy against tested endpoints. We designated therapeutic strategies based on the authors' stated intent rather than what may be a treatment's primary mechanism since some of them had pleiotropic effects. As a result, the same treatment may be categorized under multiple therapeutic strategies across publications. If more than one treatment was tested in a publication, then that study was entered into Table S1 more than once in a separate row. If a publication used multiple mouse models that met our inclusion criteria, then each model and its corresponding endpoint result were entered in the same row of Table S1, separated by commas, and considered a unique treatment evaluation in our analyses.

Therapeutic endpoints included measures of pathological tau, tau‐modifying enzyme modulation, treatment response biomarkers, neuron/brain volume loss, synaptic degeneration, synaptic dysfunction, immune response, autophagy/ubiquitin proteasome system (UPS) markers, cognitive deficits, behavioral and psychological symptoms of dementia (BPSD)/activities of daily living (ADLs), motor deficits, and survival. Table S2 lists the molecules measured and assays used on the *MAPT* mouse models to evaluate these endpoints. Extracted pathological tau data included measures of tau burden and tau PTMs and included the following endpoints: phosphorylation, glycosylation, conformational change/misfolding, acetylation, cleavage, insoluble/aggregated tau, tau seeding, tau inclusions/NFTs, total tau, and other tau. Total tau was defined as levels of tau in whole cell or soluble fractions of lysates from brain or spinal cord that were analyzed by western, ELISA, or immunofluorescence. Soluble, unfolded tau is not pathogenic, but we included it in the pathological tau summary category, as many studies that aimed to reduce tau burden measured total tau levels as an endpoint. Other tau was defined as any tau endpoint not included in the categories described earlier.

Treatment effects on the foregoing endpoints for each study are provided in Table S1 and were designated as having a statistically significant therapeutic effect (TE), no TE/detrimental/undesired effect (NE), or not tested/not quantified (NT). These designations were based on the comparison between the untreated and treated *MAPT* mouse groups regardless of whether the untreated *MAPT* mice exhibited a significant difference compared to the wild‐type (WT) untreated group. Endpoints lacking quantification or statistical analysis were marked as NT unless a systematic qualitative analysis was performed, clear visual differences were presented, or statistically significant effects were described in the text. If data were described in the manuscript text but not shown in a figure (e.g., when the text specifically states “data not shown”), the designation was based on the author's description. Total tau was marked as TE only if it was altered in whole cell lysates or soluble fractions. Other pathological tau endpoints were marked as TE if they changed in whole cell lysates, soluble, and/or sarkosyl insoluble fractions. The effects of each treatment strategy on our included therapeutic endpoints were plotted visually as heatmaps along with a subset of other key data from Table S1.

For acute treatments, the treatment end age was defined as the age at sacrifice or the age at testing if the mice were not euthanized for final experiments (e.g., if mice were only used for behavioral testing and no euthanization date was provided) and the treatment duration was marked as acute. For chronic treatments, the treatment end age was defined as the age at the last treatment, and the treatment duration was calculated as the difference between the treatment end and start age if it was not provided by the study authors.

ClinicalTrials.gov was searched to determine whether the treatments described in the included studies have been used in clinical trials for tauopathy. We searched for the conditions/diseases “tauopathies” and for the intervention/treatment named in the treatment column of Table S1, which was obtained from the cited publication. We did not perform an exhaustive search of all possible synonyms for a treatment. The “Clinical Trials ‘Tauopathies’” column of Table S1 indicates whether the treatment has been tested in a human tauopathy study, including primary tauopathies and AD.

The *MAPT* mouse models discussed in this review are listed in Table S3, along with their *MAPT* mutation, tau isoform, promoter, and background strain. Information about the disease course of these mice is also provided, including the earliest timepoints at which tau hyperphosphorylation, NFT/inclusion formation, neuronal loss, synaptic loss, long‐term potentiation/long‐term depression alterations, and cognitive impairment were reported to occur. Since it was not feasible to perform an exhaustive search of publications on each of these mouse models, the information in Table S3 was obtained from the AlzForum research models database^34^ or summarized from the primary paper and/or subsequent papers from the same research laboratory initially describing the mouse line (Table S3; Supplemental References 2).

### 2.4 Data analysis

Analysis of the data and figure generation was conducted in R version 4.4.1.^43^

## 3 RESULTS

### 3.1 Therapeutic studies in tauopathy mouse models: selection and data extraction

This review focuses on scoping therapeutic strategies for tauopathies studied in *MAPT* mouse models, excluding mouse models with other disease‐driving genetic or chemically induced modifications. To identify such preclinical studies, our search terms included mouse models with a *MAPT* knock‐in or transgene, many of which also had one or more *MAPT* mutations leading to tau pathology; mouse models such as the 3xTg mice, which also have mutations that promote amyloid pathology, were excluded.^44^ The 23 unique *MAPT* mouse models used in our search were found on the AlzForum research model database^34^ and were used as keywords to systematically query PubMed and Embase for preclinical studies evaluating potential tauopathy therapeutics. Although keywords from these mouse models guided our initial literature search, 10 of the 23 mouse models from AlzForum did not appear in the search results, and 14 additional tauopathy mouse models were identified. A complete list of the tauopathy mouse models included in this review is provided in Table S3.

Our methodical literature search for therapeutic studies in *MAPT* mouse models identified 2283 records from PubMed and Embase databases and 22 records from other sources such as internet searches and reference list checks, for a total of 2305 articles (Figure 1). After removing 856 duplicate records, we excluded 792 studies based on their titles or abstracts and two due to lack of availability in English. The full text of the remaining 655 articles was reviewed, and 341 of these articles were excluded using our predefined criteria. Ultimately, we included 314 studies in this review (Figure 1; Table S1). Some studies evaluated multiple treatments or a single treatment in multiple mouse models, and each of these was recorded as a unique evaluation, resulting in a total of 409 unique treatment evaluations. Detailed information from each treatment evaluation was extracted and tabulated in Table S1, from which all remaining figures were derived. Key data from this table are shown as heatmaps in Figure 2 and Figure S1.

FIGURE 2Summary of endpoint evaluations and their therapeutic outcomes grouped by treatment strategy. The heatmap shows all 409 treatment evaluations included in this review grouped by therapeutic strategy and the effect observed on each of the 12 primary endpoints. The pathological tau endpoint is composed of 11 measures of tau burden and post‐translational modifications, and the effectsof each evaluation on these can be seen in Figure S1. In the endpoint columns, green indicates that a therapeutic effect was reported, pink indicates that the endpoint was assessed but there was no therapeutic effect, and gray indicates that the endpoint was not tested. The dark gray column provides the name/line of *MAPT* mouse model used for each evaluation (see Table S3 for a full description of each model). Treatments that have been or are currently being tested in clinical trials as determined by a search of clinicaltrials.gov are denoted in blue. Treatments that are not or have not been in clinical trials are denoted in orange. The last column provides the reference for each evaluation (Supplemental References 1). Abbreviations are defined in Table S4.

**Figure:**
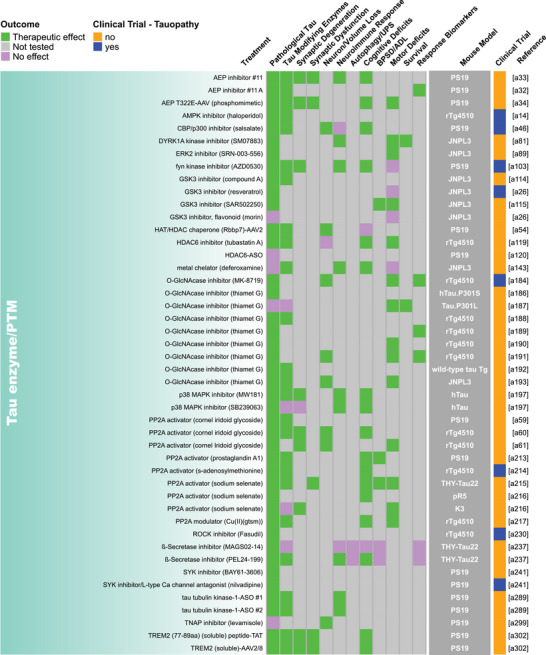

**Figure:**
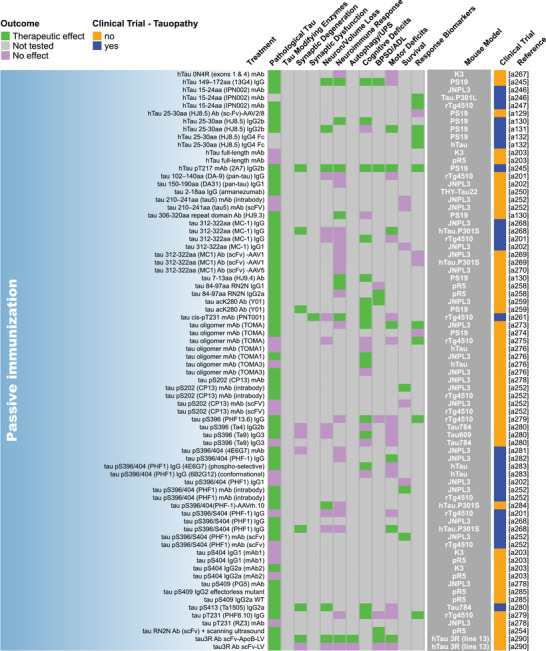

**Figure:**
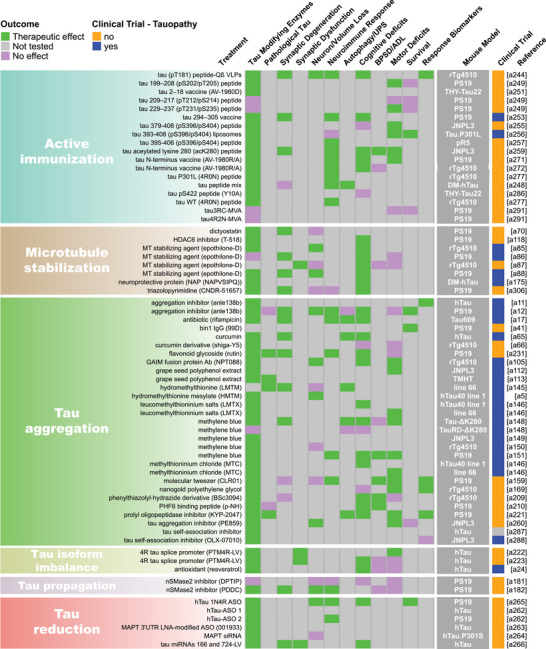

**Figure:**
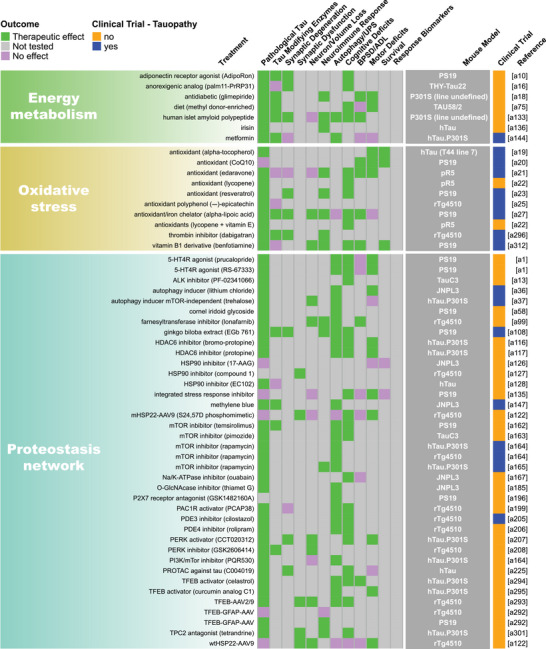

**Figure:**
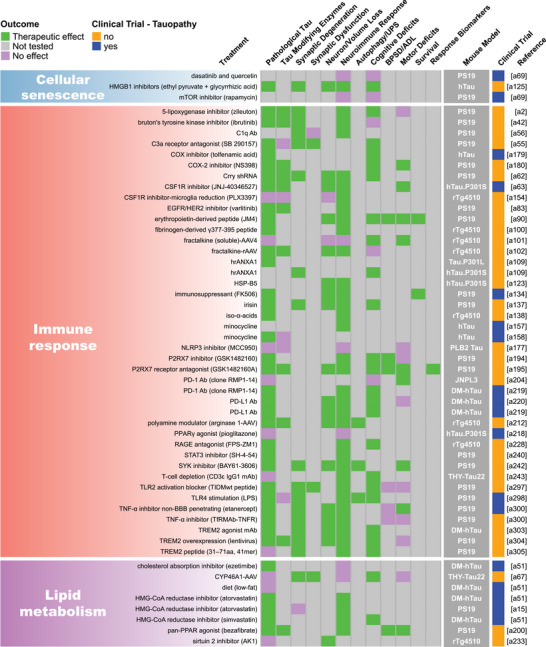

**Figure:**
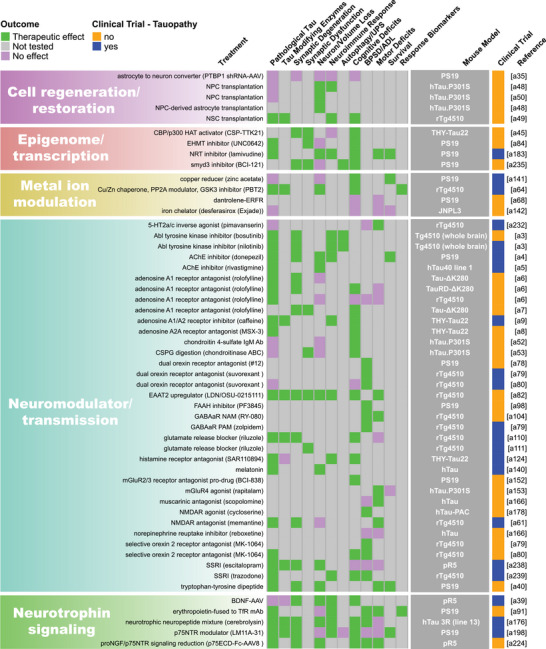

**Figure:**
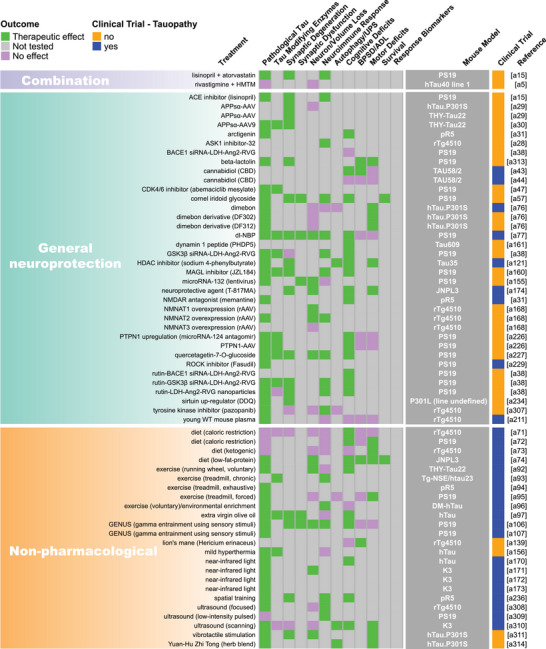

### 3.2 Tauopathy mouse models used in preclinical therapeutic research

**FIGURE 3:**
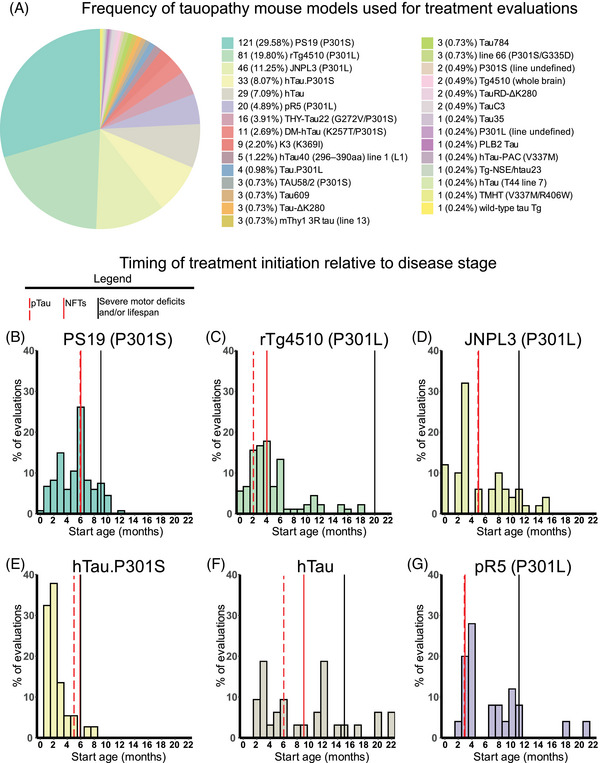
Relative frequency of mouse models and treatment start ages used in tauopathy therapeutic studies. (A) Pie chart showing number and percentage of total evaluations using each *MAPT* mouse model identified in our literature search among all 409 treatment evaluations. (B–G) Histograms showing relative frequency of treatment start ages used for therapeutic evaluations in each of the top six most common mouse lines. Solid red vertical lines indicate reported age of neurofibrillary tangle formation for each model. Dashed red vertical lines indicate reported age of onset of hyperphosphorylated tau (pTau) for each model. Solid black vertical lines indicate time to severe motor deficits that limit the lifespan, or the oldest age of mice used in the primary study when lifespan or time to severe deficits was undetermined, suggesting mice may live to that age or beyond. (B) PS19. (C) rTg4510. (D) JNPL3. (E) hTau.P301S. (F) hTau. (G) pR5.

Our search found 27 distinct *MAPT* mouse models used for the 409 treatment evaluations (Figure 3A; Table S3). Three evaluations used *MAPT* mice with P301S or P301L mutations but did not provide the specific mouse line, so they were not counted toward the total number of distinct mouse models (Figure 3A). Ten of the 23 transgenic/knock‐in/knock‐out *MAPT* mouse models found on AlzForum, specifically hTau‐A152T, hTau40‐AT, rTgTauEC, RW Tg, hTau (R406W) Tg, Tau264, Tau4RTg2652, Tau exon 10 knock‐out, Tau V337M, and *MAPT* knock‐in mice, did not appear in our search results, indicating that, to our knowledge, these models have not been used for published therapeutic research for tauopathies.

Of the 27 mouse models identified in our PubMed/Embase search, one was a *MAPT* knock‐in (PLB2 Tau), one was transgenic for WT human tau with the endogenous mouse *MAPT* knocked out (hTau), and 25 were transgenic. The transgenic mouse models featured various *MAPT* mutations, including lines with single mutations, such as PS19 (P301S) and JNPL3 (P301L), and others with multiple *MAPT* mutations, including Thy‐Tau22 (G272V, P301S). Most of the transgenic models expressed a single transgene; however, three were bi‐genic and employed the tetracycline‐off system, which includes an additional transgene to drive tau expression that can be suppressed by doxycycline. Six of the transgenic *MAPT* mouse models were used for over 80% of the 409 treatment evaluations, indicating the popularity of a few predominant mouse models for tauopathy therapeutic validation; these were as follows: PS19 (P301S) with 29.6% of all evaluations, rTg4510 (P301L) with 19.8%, JNPL3 (11.3%), hTau.P301S (8.1%), hTau (7.1%), and pR5 (4.9%) (Figure 3A). Five of the six most popular mouse models incorporated *MAPT* mutations at proline 301 (P301L or P301S), which have been associated with various forms of familial FTLD‐tau.^45^ The two most prevalent models, PS19 (P301S) and rTg4510 (P301L), both exhibit NFT‐like pathology, gliosis, neuronal loss, synapse loss, and cognitive impairment^46^, ^47^, ^48^, ^49^ (Table S3). The remaining 23 models (including the two that did not specify a mouse line) were used for approximately 20% of the evaluations, and individually, each of these lines was used in less than 4% of the total evaluations (Figure 3A). These *MAPT* mouse models included those expressing WT human tau,^35^, ^50^, ^51^, ^52^ truncated tau,^53^, ^54^ a tau aggregation promoting mutation (G335D),^55^ or tau with various FTLD‐tau‐associated *MAPT* mutations, including V337M^56^, ^57^, ^58^ and ΔK280,^59^, ^60^ and differ in age of pathology onset, spatiotemporal progression of tau pathology, and behavior (Table S3). The diversity of models available enables flexibility when targeting specific disease aspects or patient populations but may not capture upstream disease processes leading to sporadic tauopathies.

### 3.3 Timing of treatment initiation relative to tau pathology onset in MAPT mouse models

The pathological stage at which treatments are started can greatly influence their efficacy. Thus, we examined when treatment was initiated in relation to when tau pathology, particularly hyperphosphorylated tau and NFTs, was detected in specific *MAPT* mouse models. Treatment initiation times varied among the top six most utilized tauopathy mouse models, thereby reflecting a mix of approaches aimed at either preventing or addressing established tau pathology. Most treatments (64%) were initiated at or before the earliest reported occurrence of NFTs, which coincided with or followed tau hyperphosphorylation. Specifically, 71.6% of the treatment evaluations using PS19 mice started at a timepoint before NFTs are known to become detectable, 56.7% for rTg4510, 58.0% for JNPL3, 94.6% for hTau.P301S, 53.1% for hTau, and 24.0% for pR5 mice (Figure 3B–G). Fewer treatments (26%) were started after pathology progressed, specifically at least 2 months after first NFT detection, with 17.2% in PS19 mice, 27.8% for rTg4510, 40.0% for JNPL3, 2.7% for hTau.P301S, 46.9% for hTau, and 48.0% for pR5 mice. Notably, most (76%) treatments in pR5 mice were initiated after tau hyperphosphorylation and NFT development possibly due to the early onset of pathology (3 months), leaving a smaller window for pre‐pathology treatment (Figure 3G). The window for treatment initiation in the *MAPT* mouse models may also be influenced by lifespan or when severe symptoms develop as some models, including PS19 and hTau.P301S, which develop severe motor deficits by 9 and 5 to 6 months, respectively, and often become moribund soon after (Figure 3B,E).^46^, ^61^

### 3.4 Routes of treatment administration used in preclinical evaluation of tauopathy therapeutics

**FIGURE 4:**
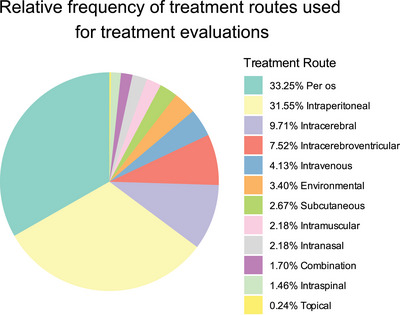
Relative frequency of treatment routes used in tauopathy therapeutic studies. Pie chart showing relative frequency of use of each treatment administration route identified in our literature search among all 409 treatment evaluations.

Across the 409 treatment evaluations, the most common administration route was per os (i.e., by mouth, including oral gavage and ad libitum drinking), accounting for 33.3% of evaluations. Intraperitoneal injection was also commonly used in these preclinical studies (31.6% of evaluations), though this route is of less clinical relevance.^62^, ^63^ Relatively invasive procedures that bypass the blood–brain barrier,^64^ including intracerebral (injection into brain tissue, 9.7%) and intracerebroventricular (injection/infusion into the cerebral ventricles, 7.5%) injections, were also popular and often used to deliver viral vector‐based gene therapies. Other treatment routes included intravenous (4.1%), environmental exposure (3.4%), subcutaneous (2.7%), intranasal (2.2%), and intramuscular (2.2%). Combination treatment routes, intraspinal injections, and topical administration were rare, together comprising less than 4% of the evaluations (Figure 4; Table S1).

### 3.5 Therapeutic strategies evaluated in tauopathy mouse models

**FIGURE 5:**
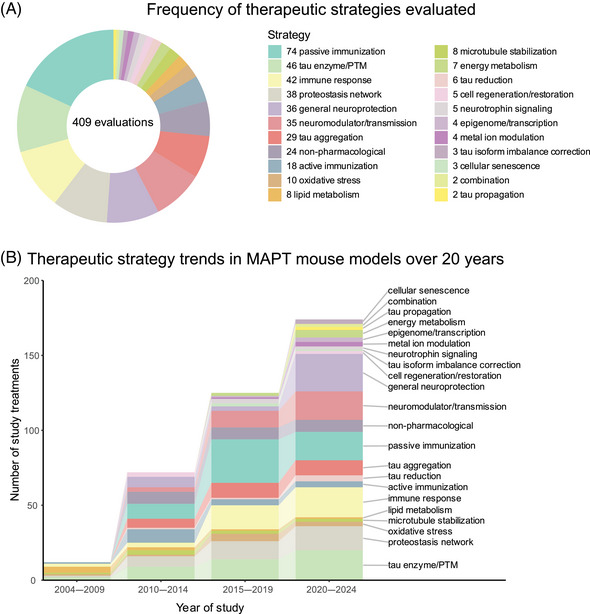
Trends in tauopathy therapeutic strategies evaluated in *MAPT* mouse models over 20 years of tauopathy studies. (A) Pie chart showing frequency of evaluation for each therapeutic strategy among the 409 therapeutic evaluations included in this review. (B) Alluvial plot showing frequency of publications assessing each therapeutic strategy over 20 years, divided into 5‐ to 6‐year bins. Publications evaluating more than one treatment were counted twice.

Our literature search revealed 22 distinct categories of therapeutic strategies for tauopathy across the 409 treatment evaluations (Figure 5A). Six involved direct targeting or modification of tau or its forms, including passive and active immunization against tau, tau aggregation inhibition, tau propagation inhibition, tau reduction via antisense oligonucleotides and similar approaches, or tau isoform imbalance correction. Of all 409 evaluations, the most frequently investigated therapeutic strategy was passive immunization using tau antibodies, with 74 evaluations (18.1% of total evaluations). A variety of tau antibodies were used, including those targeting the N‐terminus and mid‐region of tau, specific tau phosphorylation sites (e.g., pS202, pT217, pT231, pS396/404, pS396, pS404, pS409, or pS413) or oligomeric (TOMA), conformation‐specific (MC1), or acetylated forms of tau (Figure 2, Figure S1). This emphasis on tau immunotherapies reflects their dominance in current therapeutic development and aligns with recent reports showing that immunotherapies account for 36% of tau‐related projects in discovery and development in primarily industry settings.^33^ Modulation of tau‐related enzymes was the strategy used in 46 evaluations (11.2%) and included pharmacological inhibition or stimulation of enzymes that regulate tau PTMs, including those that regulate truncation (e.g., delta or beta secretases), phosphorylation (e.g., GSK3, Spleen Tyrosine Kinase [SYK], Fyn, mitogen‐activated protein kinase [MAPK], extracellular signal‐regulated kinase [ERK], dual specificity tyrosine‐phosphorylation‐regulated kinase 1a [DYRK1a], and PP2A), glycosylation (e.g., O‐GlcNAcase), or acetylation (e.g., HDAC6). Although inhibiting tau aggregation is the second most common therapeutic approach in development at primarily biotechnology and pharmaceutical companies (23%),^33^ this strategy was investigated in only 7% of the preclinical evaluations in *MAPT* mouse models (29 evaluations) identified in our literature search. The tau aggregation inhibitors included methylene blue and its derivatives, curcumin and a curcumin derivative, and grape seed polyphenol extract. Other treatment strategies directly targeting tau included inhibition of tau propagation (nSMase2 inhibitors, 2 evaluations); active immunization using tau peptides (N‐terminal, mid‐region, and C‐terminal peptides), some of which were phosphorylated [pS202/pT205, pT212/pS214, pT231/pS235, pS396/pS404, pS422] or acetylated [acK280] (18 evaluations, 4.4%); reduction of tau levels using siRNA or antisense oligonucleotides (six evaluations); or correction of tau isoform imbalance (three evaluations) (Figure 5A).

The other therapeutic strategies targeted processes that indirectly affect tau pathology or aimed to provide neuroprotection. Of these, the most common categories were immune response modulation (42 evaluations, 10.3%), proteostasis network enhancement (38 evaluations, 9.3%), general neuroprotection (36 evaluations, 8.8%), and neuromodulation/transmission modulation (35 evaluations, 8.6%) (Figure 5A). Immune response modulators included non‐steroidal anti‐inflammatory drugs (NSAIDs) and treatments targeting the complement system, immune cell receptors (P2RX7, TREM2, RAGE, PPARγ, TLR4, PD‐1, CSF1R), inflammatory cytokines (TNFα), and chemokines (fractalkine) (Figure 2, Figure S1). Strategies aimed at enhancing proteostasis networks to eliminate pathological tau species included modulating autophagy, the ubiquitin‐proteosome system, the unfolded protein response, or heat‐shock proteins. A variety of therapeutic strategies were categorized as generally neuroprotective in that the study investigators aimed to maintain overall brain health and function using treatments with pleiotropic, undefined, or multitargeted mechanisms. Examples of treatments evaluating this strategy include natural products (e.g., plant‐derived arctigenin or whey‐derived beta‐lactolin), plasma transfusion from young healthy mice, and repurposed drugs (e.g., dimebon or fasudil). The neuromodulator/neurotransmission treatment strategies involved pharmacological targeting of neurotransmitter systems, including glutamate (most common), adenosine, acetylcholine, GABA, or serotonin, or of neuropeptides, such as orexin. Non‐pharmacological strategies (24 total evaluations, 5.9%) included exercise, dietary modifications, environmental enrichment, cognitive training, gamma oscillation entrainment, photobiomodulation, and opening of the blood–brain barrier (Figure 2, Figure S1). Finally, the therapeutic strategy categories with the fewest evaluations, each accounting for less than 3% of the total evaluations (Figure 5A), included targeting oxidative stress (e.g., antioxidants), lipid metabolism (e.g., statins and other cholesterol‐ and lipid‐lowering strategies), microtubule stabilization (e.g., epothilone D), energy metabolism, cell regeneration/restoration (e.g., neural and glial stem cell transplantation), neurotrophin/neurotrophin receptor signaling (e.g., p75^NTR^ modulation, BDNF delivery), epigenome/transcription (e.g., transcriptase or transferase inhibitors), metal ion modulation (e.g., iron or copper modulators), cellular senescence, or combination strategies (i.e., those using two or more treatments with different therapeutic strategies). In all, these strategies reflect diverse approaches, including directly targeting tau and targeting pathways or mechanisms that interact with tau.

Interest in different therapeutic strategies for tauopathy has evolved over the 20 years of preclinical research included in this review. The number of treatments studied in transgenic and knock‐in *MAPT* mouse models of tauopathy has increased steadily since 2004 as more of these mouse models have become available since early published descriptions in 1999 (Figure 5B; Table S3). Treatment studies targeting immune responses have increased greatly over the past 10 years, and in the last 5 years, treatments aimed at providing general neuroprotection have become more popular (Figure 5B). The latter strategy largely includes evaluations of repurposed drugs/compounds, including memantine, fasudil, abemaciclib mesylate, lisinopril, and cannabidiol. Interest in other therapeutic strategies has increased steadily over time, including regulating the proteostasis network, tau enzyme/PTMs, and neuromodulation/transmission. A large surge in passive immunization treatments in tauopathy models was seen from 2010–2014 to 2015–2019, which has fallen in recent years. Finally, combination therapies and therapeutic strategies aimed at reducing tau or its propagation, normalizing energy metabolism, and preventing cellular senescence in *MAPT* mouse models have only received attention in the past 5 years (Figure 5B).

### 3.6 Common endpoints used in therapeutic evaluations in tauopathy mouse models

**FIGURE 6:**
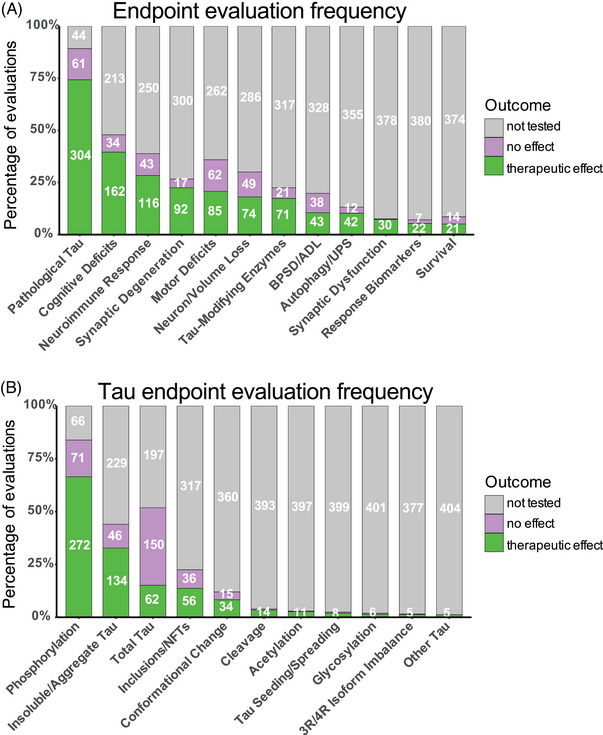
Endpoint assessment frequency in tauopathy mouse models. Stacked bar graph showing frequency of endpoint assessment among all 409 treatment evaluations included in this review and whether an effect was measured. Green indicates a therapeutic effect on specified endpoint was reported, pink indicates absence of therapeutic effect on specified endpoint was reported, gray indicates specified endpoint was not tested. (A) Main endpoints. (B) Pathological tau endpoints.

We identified 12 primary endpoints against which treatments were commonly evaluated in tauopathy mouse models. These endpoints include measures of pathological tau, tau‐modifying enzymes, brain volume/neuronal loss, synaptic degeneration, synaptic dysfunction, neuroimmune response, autophagy/UPS‐mediated degradation of tau, cognitive deficits, behavioral and psychological symptoms of dementia (BPSD)/ADL, motor deficits, survival, and treatment response biomarkers (Figure 6A). The assays and measures commonly used to evaluate each endpoint are summarized in Table S2. Pathological tau is a summary category that includes measures of tau burden and PTMs and encompasses 11 tau‐specific endpoints, which are phosphorylation, glycosylation, acetylation, cleavage, conformational change/misfolding, insoluble/aggregated, inclusions/NFTs, 3R/4R isoform imbalance, seeding/spreading, total soluble tau protein levels, and a miscellaneous “other tau” category, including changes in sumoylated, ubiquitinated, or nitrated tau, or tau mRNA (but not protein levels) (Figure 6B; Table S2). Although soluble, unfolded tau is not pathogenic, it was included in the pathological tau category as it was frequently used as an endpoint in studies aimed at reducing tau burden. Other less commonly used “non‐tau” endpoints included cell stress and damage (e.g., oxidative stress, DNA damage, ER stress, apoptosis, heat shock proteins, and chaperones); energy metabolism (e.g., mitochondrial function, insulin and glucose metabolism); lipid homeostasis; senescence and cell cycle regulation; epigenomic, transcriptomic, proteomic, and metabolomic changes; APP/Aβ/amyloid plaques; calcium, copper, and zinc homeostasis; axonal transport; neurotrophic factors and signaling; neurotransmitters and their receptors; and extracellular matrix integrity (perineuronal net degradation). These other “non‐tau” endpoints were noted in Table S1 but were not included in our analysis of the 12 commonly used endpoints below.

### 3.7 Endpoint assessment frequency in tauopathy mouse models

Pathological tau was the most frequently evaluated endpoint (365 evaluations, 89.2%), and positive therapeutic effects were reported in 304 of the 365 evaluations (83.3%). Notably, 44 of the total 409 evaluations (10.8%) in tauopathy mouse models did not assess pathological tau (Figures 2 and 6A). Among the 11 pathological tau endpoints, hyperphosphorylated tau (pTau) was the most frequently assessed (343 evaluations, 83.9%) at about 15 different phosphorylation sites (Table S2), with 272 of these evaluations reporting positive effects (79.3%, Figures 2 and 6B). Insoluble/aggregated tau was assessed in 180 evaluations (44.0%), inclusions/NFTs in 92 evaluations (22.5%), and tau misfolding/conformational change in 49 evaluations (12.0%). Total tau levels were examined in 212 evaluations (51.8%), with 29.2% of them showing a significant decrease after treatment, likely because in most cases total tau levels were assessed as a control for other tau changes and were not directly targeted. Other pathological tau‐specific endpoints that were infrequently measured included glycosylation, acetylation, nitration, sumoylation, ubiquitination, 3R/4R isoform imbalance, cleavage, and tau seeding/spreading (Figure 6B, Figure S1). These results indicate a reliance on the heterogenous pTau endpoint and underevaluation of other tau species that contribute to neurodegeneration.

Other commonly assessed “non‐tau” endpoints were cognitive deficits (196 evaluations, 47.9%), neuroimmune response (159 evaluations, 38.9%), and motor deficits (147 evaluations, 35.9%). Endpoints assessed in fewer than 30% of evaluations were neuronal loss/brain volume changes, synaptic dysfunction, autophagy/UPS‐mediated degradation of tau, BPSD/ADL, survival, treatment response biomarkers, and synaptic degeneration. The synaptic degeneration endpoint was assessed in 109 evaluations and included 95 evaluations for synaptic markers, 18 of which included measures of dendritic spine density, and 19 evaluations of degenerating/dystrophic neurites. Mouse survival was assessed only in 35 evaluations (8.6%). However, this measure is manageable primarily in short‐lived mouse models. For example, among the top six most frequently used mouse models, three progress to severe motor deficits and become moribund before 12 months of age (PS19, 9 m; JNPL3, 11 m; hTau.P301S, 5 to 6 m). Survival was used as an endpoint in 18 evaluations in PS19 mice, 13 in JNPL3 mice, and one in hTau.P301S mice (Figure 3B,D,E; Tables S1 and S3). The other three models do not have a documented time to severe motor deficits or a defined lifespan and likely live to or beyond the oldest age reported in their primary publications (rTg4510, > 20 m; hTau, > 15 m; pR5, > 11 m), making survival studies in these models difficult, and accordingly, survival was not assessed in these mouse models for the evaluations included in this review (Figure 3C,F,G; Tables S1 and S3).

### 3.8 Frequently evaluated therapeutic strategies that consistently yield therapeutic effects on key endpoints

**FIGURE 7:**
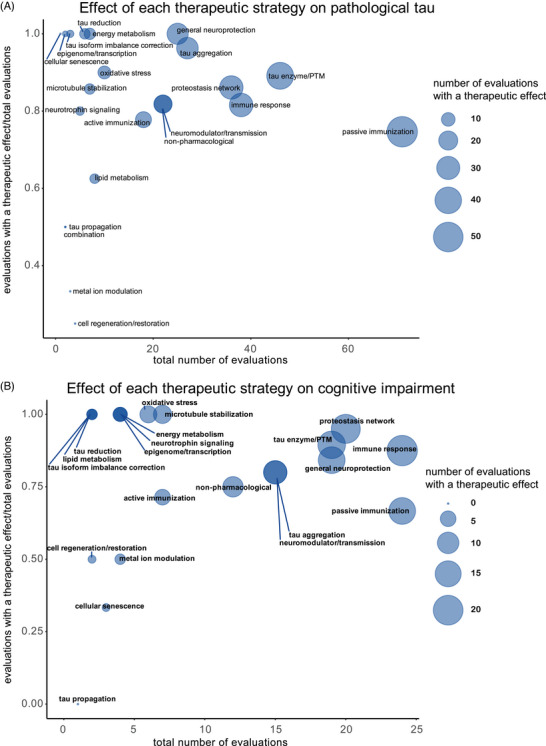
Therapeutic strategies and their reported effects on key endpoints in *MAPT* mouse models. Proportion of evaluations reporting therapeutic effects on the two most popular endpoints identified in this review: (A) pathological tau and (B) cognitive deficits. Each bubble represents a different therapeutic strategy. The *x*‐axis shows the total number of evaluations for each strategy, and the *y*‐axis indicates the proportion of evaluations reporting a therapeutic effect relative to the total number of evaluations. The bubble size corresponds to the total number of evaluations reporting a therapeutic effect.

To identify well‐studied treatment approaches with consistent and robust positive effects in tauopathy mouse models, we examined the frequency with which each treatment strategy was tested against the two most frequently measured endpoints – pathological tau and cognitive deficits – and the proportion of evaluations that elicited a therapeutic effect on each endpoint (Figure 7). Treatment strategies that repeatedly demonstrate therapeutic effects stand out as those of interest for further research; however, their efficacy requires validation through additional studies or systematic review and meta‐analysis of treatment‐specific literature.

Passive immunization was the most frequently evaluated strategy (71 evaluations) for reducing pathological tau, with most studies (75%) reporting a therapeutic effect (Figure 7A). This strategy was also frequently evaluated (against cognitive deficits), with a weak majority (67%) showing a therapeutic effect (Figure 7B). Several other strategies, including those targeting tau enzymes/PTMs, proteostasis network, immune response, tau aggregation, and general neuroprotection, were frequently evaluated against both pathological tau (25 to 46 evaluations) and cognitive deficits (15 to 24 evaluations), and a high proportion of them (>80%) showed therapeutic effects (Figure 7). Neuromodulation/transmission strategies were frequently evaluated against cognitive deficits (15 evaluations), with a high proportion of evaluations (80%) showing therapeutic effects (Figure 7B), aligning with their aim to ameliorate cognitive deficits without directly targeting tau pathology. Although neuromodulation/transmission strategies were infrequently tested against pathological tau (22 evaluations), when they were, many of the evaluations (82%) showed a therapeutic effect (Figure 7A). Therapeutic strategies with few (<10) but effective (>80% with therapeutic effects) evaluations against both pathological tau and cognitive deficits include those targeting tau reduction via antisense oligonucleotides and similar approaches, energy metabolism, neurotrophin/neurotrophin receptor signaling modulation, epigenome/transcription, oxidative stress, and microtubule stabilization (Figure 7). These less frequently evaluated but notable strategies may warrant further investigation.

### 3.9 Treatments with therapeutic effects across multiple endpoints

We aimed to identify individual treatments or groups of related treatments that have been well characterized (i.e., evaluated multiple times, in multiple *MAPT* mouse models, and/or across many of the 22 endpoints examined in this review) and that have demonstrated therapeutic effects against many of the 22 endpoints. To do this, we grouped treatments by target, such as neurotransmitter receptors, tau phospho‐sites, or tau modulatory enzymes. This grouping resulted in single treatments (e.g., epothilone‐D or LM11A‐31), groups of related treatments (e.g., methylene blue and its derivatives), and broader groups of multiple treatments (e.g., the antioxidant group that includes resveratrol, lycopene, vitamin E, coenzyme Q10, and epicatechin). We tallied the number of evaluations for each treatment/treatment group, as well as the number of endpoints, therapeutic effects, and different mouse models used for these evaluations. Each endpoint was counted once per treatment/treatment group, regardless of the number of times it was measured (Table 1). Antibodies targeting tau phosphorylated at S396/S404 (13 evaluations), O‐GlcNAcase inhibitors (thiamet‐G and its derivative MK8719; 10 evaluations), and antioxidants (10 evaluations) were evaluated the most frequently, tested across —four or five different *MAPT* mouse models, and characterized across multiple endpoints (11, 10, and 13 different endpoints, respectively) with many therapeutic effects (on 9, 10, and 12 endpoints, respectively) (Table 1; Figure 2, Figure S1). The tau aggregation inhibitor methylene blue and its derivatives were also well characterized, with nine evaluations across seven different mouse models and testing against 14 of 22 endpoints, showing therapeutic effects on 13. Methylene blue was assessed on six of the 11 pathological tau endpoints and demonstrated a therapeutic effect on five of them (Figure S1). Tau reduction, via antisense oligonucleotides and similar approaches, was also a notable strategy as it had six evaluations in two *MAPT* mouse models and was evaluated across 13 of 22 endpoints with therapeutic effects on all 13. Tau reduction approaches were evaluated across six tau pathology endpoints and positively affected six. The p75 neurotrophin receptor (p75^NTR^) modulator, LM11A‐31, was tested in only one *MAPT* mouse model; however, it was evaluated across the greatest number of endpoints (17 out of 22 total) with positive effects against 12 of them, indicating broad characterization and beneficial effects across multiple disease processes. LM11A‐31 was also assessed against the greatest number of pathological tau endpoints (8 out of 11) and demonstrated a positive effect on seven of the eight, with soluble total tau as the only endpoint not affected (Table 1, Figure 2, and Figure S1). Treatments that have positive effects across multiple primary endpoints and/or pathological tau‐related endpoints may be necessary to effectively address multifaceted diseases like tauopathies and may have heightened potential for clinical translation.

**TABLE 1:** Summary of endpoint evaluations across treatment groups.

Treatment	Strategy	Endpoints measured (count)	Endpoints with a therapeutic effect (count)	Tau endpoints measured (count)	Tau endpoints with a therapeutic effect (count)
Methylene blue/methylene blue derivative	Tau aggregation	14	13	6	5
Tau knockdown	Tau reduction	13	13	7	7
p75^NTR^ modulator (LM11A‐31)	Neurotrophin signaling	17	12	8	7
Antioxidant	Oxidative stress	13	12	4	4
EAAT2 upregulator (LDN/OSU‐0215111)	Neuromodulator/transmission	11	11	4	4
Tau pT217 antibody	Passive immunization	12	11	5	4
Asparagine endopeptidase (AEP) inhibitor	Tau enzyme/PTM	11	11	4	4
Quercetagetin‐7‐O‐glucoside	General neuroprotection	10	10	5	5
Adenosine receptor antagonist	Neuromodulator/transmission	14	10	6	5
hTau25‐30aa antibody	Passive immunization	10	10	5	5
TFEB activator/upregulator	Proteostasis network	11	10	4	3
Aggregation inhibitor (anle138b)	Tau aggregation	14	10	5	4
O‐GlcNAcase inhibitor	Tau enzyme/PTM	10	10	5	5
P2X7 receptor antagonist (GSK1482160)	Immune response	10	9	3	2
Exercise	Non‐pharmacological	11	9	4	4
Tau pS396/404 antibody	Passive immunization	11	9	5	4
PP2A activator/modulator	Tau enzyme/PTM	10	9	4	3
Cornel Iridoid glycoside	Tau enzyme/PTM	10	9	5	4
Tau (pT181) peptide‐Qβ VLPs	Active immunization	9	8	4	3
Human islet amyloid polypeptide (h‐IAPP)	Energy metabolism	10	8	3	2
Cornel Iridoid glycoside	General neuroprotection	8	8	3	3
Erythropoietin	Immune response	9	8	3	2
Fractalkine	Immune response	9	8	4	3
Polyamine modulator (arginase 1‐AAV)	Immune response	9	8	5	4
Epothilone‐D	Microtubule stabilization	10	8	4	4
Neurotrophic neuropeptide mixture (cerebrolysin)	Neurotrophin signaling	9	8	2	2
Tau MC‐1 antibody (conformation specific)	Passive immunization	12	8	5	5
Tau3R antibody	Passive immunization	9	8	3	2
PERK activator (CCT020312)	Proteostasis network	8	8	4	4
Farnesyltransferase inhibitor (lonafarnib)	Proteostasis network	9	8	5	4
mTOR inhibitor	Proteostasis network	8	8	5	5
Molecular tweezer (CLR01)	Tau aggregation	11	8	5	4
Prolyl oligopeptidase inhibitor (KYP‐2047)	Tau aggregation	8	8	3	3

*Note*: The table lists the number of endpoints each treatment/treatment group was tested against and the number of endpoints with reported therapeutic effects. Treatments are grouped by target and include single treatments, groups of closely related treatments (e.g., methylene blue and its derivatives), or broader categories with multiple treatments (e.g., antioxidants such as resveratrol, lycopene, vitamin E, coenzyme Q10, and epicatechin). Each endpoint was counted once per treatment group, regardless of the number of evaluations. Abbreviations are defined in Table S4.

Abbreviations: AAV, adeno‐associated virus; AEP, asparagine endopeptidase; EAAT2, excitatory amino acid transporter 2; PP2A, protein phosphatase 2A; PTM, post‐translational modification; TFEB, transcription factor EB; VLP, virus‐like particle.

## 4 DISCUSSION

Despite major research efforts, no disease‐modifying therapeutics are available for primary tauopathies. *MAPT* mouse models of tauopathy have been essential for testing potential therapeutic strategies and garnered many positive results, but translating these results to effective human treatments remains a significant challenge. We compiled data from 20 years of preclinical research in tauopathy mouse models to determine the scope and patterns of therapeutic approaches, *MAPT* mouse models used, and the endpoints against which these treatments were measured. Given that our search started with the year that the first of the 27 single transgene/knock‐in/knock‐out *MAPT* mouse models was developed, this review is a nearly comprehensive compilation of therapeutic studies in tauopathy mice. This analysis clarified and reinforced known challenges in tauopathy research that hinder clinical translation, offering insights for refining preclinical approaches.

The complex process of tau pathogenesis and its related neurodegeneration presents multiple and diverse targets for therapeutic intervention, and many of these targets have been addressed in *MAPT* mouse models and human tauopathy trials. Of the 22 therapeutic strategies evaluated in *MAPT* mice, only cell regeneration/restoration and tau propagation have yet to be directly tested in clinical trials (Figure 2; Table S1). However, immunotherapies may interfere with tau propagation by targeting extracellular tau, and these are the most prevalent treatments in discovery and development for tauopathies.^33^, ^65^ Of the individual treatments evaluated in *MAPT* mouse models, 92 have been or are currently being evaluated in clinical trials (Figure 2), including those for primary tauopathies and AD. A total of 38 tauopathy therapeutics are in active clinical trials or have been associated with an investigational new drug/clinical trial application out of 171 in development primarily at biotech and pharmaceutical companies.^33^ The large number of potential tauopathy‐related targets and the diversity of therapeutic strategies provide opportunities to explore multiple disease mechanisms but may pose challenges for ensuring rigorous preclinical testing for a particular target/strategy and prioritizing treatments for clinical advancement.

A major challenge in developing therapeutics for tauopathies, and neurogenerative diseases collectively, is the lack of translation of promising preclinical treatments into successful human clinical trials.^26^, ^66^, ^67^ Passive immunization using antibodies against tau epitopes is the most widely studied therapeutic strategy in *MAPT* mouse models (Figure 5A), and several antibodies within this category have been tested in or are in clinical trials for tauopathies. Antibodies targeting the N‐terminus of tau (tau 25–30 aa) were evaluated five times across two different *MAPT* mouse models; six of 12 primary endpoints were assessed, and therapeutic effects were observed for all six (Figure 2). However, the tau N‐terminus (tau 25–30 aa) antibody, tilavonemab, did not demonstrate efficacy in phase 2 clinical trials for PSP and AD.^68^, ^69^ An antibody targeting a different epitope of the tau N‐terminus (15–24 aa) was assessed in three evaluations and showed positive effects in the two primary endpoints tested in three different *MAPT* mouse models, with treatment starting before or at the time of reported tau pathology in the respective models (Figures 2 and 3B). In contrast, gosuranumab (a tau 15–24 aa antibody) significantly lowered N‐terminal tau in the CSF of PSP patients compared to placebo, but this did not result in clinical efficacy in a phase 2 trial.^70^ Antibodies against tau pS396/S404^71^, ^72^, ^73^, ^74^, ^75^, ^76^, ^77^, ^78^ were assessed in 13 evaluations and have shown a high degree of success against cognitive and tau‐related endpoints in four *MAPT* mouse models, but clinical trials in primary tauopathy patients have not been reported.^26^, ^66^, ^79^, ^80^ However, an antibody against this target was tested in one AD clinical trial and later discontinued.^79^, ^80^, ^81^, ^82^ The second most frequently assessed therapeutic strategy in tauopathy mouse models is modulating tau‐related enzymes to alter pathological tau PTMs. The O‐GlcNAcase inhibitor, thiamet‐G, and its derivative MK8719^83^, ^84^, ^85^, ^86^, ^87^, ^88^, ^89^, ^90^, ^91^, ^92^ were among the most evaluated treatments in this category (nine of 46 evaluations in five different *MAPT* mouse models) with positive effects on all endpoints tested (six of 12 endpoints) (Figure 5A). Small‐molecule O‐GlcNAcase inhibitors have recently entered phase 2 trials in PSP patients.^93^, ^94^ Other tau enzyme/PTM modulation strategies included in this review and evaluated in primary tauopathy trials were lithium (a GSK3β inhibitor) and salsalate (a tau acetylation inhibitor). The phase 1 trial for lithium was halted due to patient falls, a known side effect.^66^ Salsalate was evaluated once in PS19 tauopathy mice with treatment starting after pTau emerges, and therapeutic effects were demonstrated on four endpoints (five out of 12 primary endpoints were tested) (Figure 2). However, salsalate did not affect exploratory clinical and neuropsychological outcomes in a phase 1 PSP trial.^95^ Finally, the tau aggregation inhibitor methylene blue and its derivatives^96^, ^97^, ^98^, ^99^, ^100^, ^101^, ^102^, ^103^ were frequently evaluated (nine times) in seven *MAPT* mouse models and showed the greatest number of therapeutic effects across many varied endpoints. However, TauRx's tau aggregation inhibitor, TRx0237, a methylene blue derivative, did not achieve clinical efficacy in several randomized phase 3 trials (one frontotemporal dementia trial and two AD trials), although subsequent post hoc analyses of study subgroups appear more promising.^104^, ^105^, ^106^, ^107^, ^108^, ^109^, ^110^ These examples show that although some frequently evaluated treatments that showed positive effects in multiple *MAPT* mouse models have proceeded to clinical testing for primary tauopathies, the outcomes of the clinical trials have shown limited success or are pending. This discrepancy raises the question: How can preclinical testing in *MAPT* mouse models be refined to better predict clinical trial success?

Mouse‐to‐human translation could be hindered by discrepancies in the timing of treatment initiation relative to pathology onset between mouse models and tauopathy patients. Currently, treatment initiation is limited to later and symptomatic disease stages in tauopathy patients by the lack of well‐established early diagnostic or biomarker strategies. However, most studies using the six most prevalent tauopathy mouse models began treatment early – before or at the time tau pathology was first documented – revealing a treatment's potential to prevent tauopathy rather than to modify or slow ongoing, detectable pathology. In tauopathy mouse models, the treatment start age should align with the therapeutic goal – whether to prevent neurodegeneration, slow or reverse established pathology, or provide symptom relief.^111^ Ideally, clinical treatments would be initiated early in the disease course, prior to the accumulation of neuropathology and neuron and synapse loss, which may be difficult to halt or reverse. However, the challenge of diagnosing, enrolling, and assessing treatment outcomes in preclinical patients (i.e., cognitively normal patients with biomarker changes or genetic mutations predictive of future symptom development^67^) has slowed the launch of clinical trials for preventive therapeutics.^112^ Nevertheless, trials that focus on preclinical AD patients or those with mild cognitive impairment are more common.^113^, ^114^, ^115^ In AD, changes in Aβ and tau are detectable in biofluids over a decade prior to clinical presentation.^116^, ^117^, ^118^, ^119^ Fluid biomarkers, such as pTau181, pTau217, and pTau231, support trials in preclinical AD patients by enabling cost‐effective screening and assessment prior to symptom development.^113^, ^114^, ^115^, ^120^ Despite ongoing research into fluid biomarkers for primary tauopathies, none have yet demonstrated the accuracy and utility seen with biomarkers used for AD.^33^, ^67^, ^121^, ^122^ Interestingly, translatable tau treatment response biomarkers are rarely investigated in preclinical therapeutic studies of tauopathy (29 evaluations, 7.1%), although establishing such markers could aid in clinical translation. Given these challenges, it is unsurprising that therapies validated for prevention in tauopathy mouse models often fail to translate to human patients with existing pathology. Future trials may benefit from focusing on therapeutics that can reverse or slow decline in mouse models when treatment administration is initiated in the presence of well‐established, existing pathology. Alternatively, therapeutics tested in mouse studies with early treatment initiation – before pathology and symptom development – could be assessed in comparable patient populations.

Assessing numerous and varied disease‐relevant endpoints may also greatly facilitate mouse‐to‐human translation. Over 75% of the therapeutic strategies included in this review assessed pathological tau, with tau phosphorylation, as the most frequently assessed endpoint in this category. However, more than 10 different phosphorylation sites for tau were examined inconsistently (Table S2), complicating direct comparisons between evaluations. While looking at varied endpoints can be beneficial, standardizing key endpoints across studies would improve comparability. Other pathological tau endpoints were less commonly or rarely assessed in tauopathy mouse models, including other tau PTMs, insoluble/aggregated tau, misfolding, and NFTs, providing an incomplete view of a treatment's potential. The ability to affect a broad and diverse range of pathological endpoints in one or more preclinical models indicates a robust effect and thus might better predict success in human trials. Functional endpoints relevant to tauopathies, like cognitive deficits, motor deficits, and BPSD/ADL, were assessed only in 20% to 50% of evaluations, and neuron/brain volume loss and synaptic dysfunction were measured in only 10% to 30% of evaluations. Interestingly, synaptic markers and dendritic spine density were measured in only 22% of the treatment evaluations, despite findings that loss of synaptic or spine integrity is perhaps the highest pathological correlate of cognitive dysfunction in tauopathies and related neurodegenerative disorders.^16^, ^123^, ^124^, ^125^, ^126^, ^127^, ^128^ Prioritizing measures of synapse and dendritic spine resilience in preclinical tauopathy studies may be beneficial for clinical translation. Evaluating relevant functional and cellular endpoints alongside diverse pathological tau endpoints may also provide a clearer view of a therapeutic's potential. Furthermore, due to the complex pathophysiology of tauopathies, it may be necessary to target multiple pathological processes using multifunctional treatments or a combination of treatment strategies. Assessing therapeutics for a range of endpoints may help researchers to identify where one therapeutic is lacking, allowing identification of optimal drug combinations.

The diversity of tauopathies and inherent limitations associated with tauopathy mouse models present significant challenges for translational research. Tauopathies are genetically and pathologically heterogeneous and are characterized by disease‐specific tau conformations that may differ from those found in specific transgenic *MAPT* mouse models.^21^, ^26^, ^33^, ^129^, ^130^, ^131^ Thus, matching mouse models to intended patient populations is essential for translational research.^7^ Additionally, while the mouse models discussed here exhibit useful disease‐relevant phenotypes, most rely on overexpression of a single tau isoform under the control of a heterologous promoter, potentially contributing to disease‐irrelevant phenotypes, loss of isoform diversity, and unnatural spatiotemporal expression of tau.^132^ Together, these issues may limit the generalizability of results obtained from treatment testing performed in a single mouse model. Innovative clinical trial designs such as basket trials, where a single intervention is tested across patients with different tauopathies, may benefit the early clinical development of tau therapeutics when specific patient subsets are too small for robust conclusions.^66^, ^67^, ^133^, ^134^ Preclinical trials may benefit from a similar strategy, as testing treatments across multiple mouse models with distinct strengths and limitations may help identify robust treatments that are more likely to succeed in humans. Our review revealed that 43 of the treatments/treatment groups were assessed in multiple *MAPT* mouse models, suggesting that some investigators have taken this approach (Figure 2). However, the remaining 200 treatments/treatment groups were tested in a single mouse model, leaving room for additional preclinical studies to better assess translational potential (Figure 2). The continued evolution of tau mouse models will likely enhance clinical relevance and help bridge the translational gap in therapeutic development.^132^

Given that human tauopathy patients and several tauopathy mouse models exhibit sex‐specific differences in disease phenotypes and treatment response, evaluating treatments in both sexes could provide a more complete and accurate assessment of efficacy. In humans, FTLD has a higher incidence among European males than females.^135^ Women have more severe language impairments and fewer behavioral symptoms compared to men with certain forms of FTLD.^136^, ^137^, ^138^ At diagnosis, men and women with certain forms of FTLD exhibit a similar level of executive and behavioral impairment, but women exhibit greater brain atrophy, indicating greater executive and behavioral reserve.^138^ However, once women with familial forms of FTLD become symptomatic, they experience faster cognitive decline compared to men.^139^ Sex differences also occur in tauopathy mouse models.^140^ Female rTg4510 mice show increased tau phosphorylation and more severe impairment of spatial learning and memory compared to age‐matched males despite having similar transgene expression levels,^141^ female JNPL3 mice show higher levels of insoluble tau and elevated JNK activation,^142^, ^143^ and male PS19 mice exhibit greater motor deficits and phosphorylated tau and have lower survival rates compared to females. Sex‐related differences in cognitive deficits have also been reported for PS19 mice, although the direction of these differences varies across studies.^144^, ^145^ Some of these sex‐related differences may be biologically relevant, and others may result from differences in transgene expression levels. For example, the PrP promoter drives higher transgene expression in females compared to males in some transgenic mouse lines.^146^ Despite these reported sex differences, the majority of preclinical treatment studies only investigate one sex. Of the 409 evaluations included in this review, 141 used both male and female mice, while 172 used only one sex (95 male and 77 female); the sex of the mouse was not provided for the remaining evaluations. Single‐sex or pooled‐sex studies could obfuscate sex differences in treatment response. Sex‐specific treatment effects have been observed in hTau mice treated with the hormone irisin,^147^ in hTau.P301S (i.e., Tg2541) mice treated with colony‐stimulating factor‐1 receptor (CSF1R) inhibitors,^148^ and in clinical trials using davunetide for human PSP and mild cognitive impairment, where post hoc analyses revealed sex‐specific drug effects^149^, ^150^ that were not detected in pooled‐sex analyses.^151^, ^152^ Using male and female mouse models and applying sex‐stratified analyses in clinical trials for tauopathies, as is increasingly recommended for AD research,^153^, ^154^ may improve translation.

Other barriers to translation include treatment administration routes that are uncommonly used in humans and limited pharmacokinetic data for clinically relevant routes. The most frequently used treatment administration route in *MAPT* mouse models is oral administration (per os; 33% of evaluations), which is also the most common treatment route in humans, as it is used for about 47% of the drugs in clinical trials for tauopathies^33^, ^63^ and is the most amenable to clinical translation, especially for small molecules. Intraperitoneal (IP) injections, a route rarely used in humans, was the second most common method of administration. While IP injections are valuable for proof‐of‐concept and target engagement studies, additional formulation, pharmacokinetic, and safety studies are necessary to adapt the treatment to clinically relevant routes.^62^, ^155^, ^156^ Intracerebral and intracerebroventricular injections were also common but remain impractical for routine clinical use due to their invasiveness. Many gene therapy treatments, delivered via viral vectors such as adeno‐associated virus and lentivirus, were administered through intracerebral or intracerebroventricular injection (Table S1). Although early clinical trials raised safety concerns that stalled the use of viral vectors, advances in vector engineering have since led to the approval of 13 gene therapy products.^157^ Most of these approved vectors are delivered intravenously or intramuscularly in clinical settings,^157^ so adapting tauopathy treatments to these more practical administration routes would be a priority before clinical translation. Intravenous, subcutaneous, and intramuscular injections were less frequently used in the studies included here but are frequently used in humans, with the former two often used for delivery of antibodies.^33^, ^158^ Finally, intraspinal injections were used in only ∼1.5% of the evaluations in *MAPT* mouse models, but intrathecal injections are frequently used to deliver anti‐sense oligonucleotides, a strategy that has gained popularity recently.^30^ Each of these routes may be amenable to clinical translation.

### 4.1 Limitations

Our review focused solely on *MAPT* mouse models of tauopathy, excluding research conducted in multitransgenic AD models, models that have tau pathology resulting from mutations in non‐*MAPT* genes, mouse models with comorbid pathology, models using tau protein injections, and non‐mouse models. Therefore, tau‐targeting drugs or neurodegeneration‐targeting drugs developed in these excluded tauopathy models are not covered. Due to the pleiotropic effects of many drugs, categorizing therapeutics into a single strategy is challenging. We based our categorization on the authors’ stated intent, resulting in some drugs being classified differently across evaluations. As such, our analysis is influenced by the investigators’ research objectives rather than an objective assessment of drug mechanisms. While this review does not comprehensively analyze the concordance between animal studies and clinical trials, systematic reviews comparing the two could identify gaps and areas for improvement to enhance translatability.

## 5 CONCLUSIONS

While many therapeutic interventions validated in tauopathy mouse models have appeared promising, translation into effective human treatments is not guaranteed. Timing therapeutic interventions appropriately, refining model selection, assessing an adequate variety of molecular, cellular, and functional endpoints, and exploring combination therapies may help to improve translational relevance. Prioritizing clinical development of therapeutics that have been broadly characterized across a variety of endpoints may also be advantageous.

## AUTHOR CONTRIBUTIONS

Vanessa F. Langness: Validation, Investigation, Project administration, Data curation, Writing—original draft, Writing—review & editing, Methodology. Danielle A. Simmons: Validation, Investigation, Supervision, Project administration, Writing—original draft, Writing—review & editing. Tyne L.M. McHugh: Validation, Investigation, Writing—review & editing. Robert R. Butler III: Methodology, Formal analysis, Resources. James Zhou and Harry Liu: Investigation. Tao Yang: Methodology. Lisa M. Ellerby and Frank M. Longo: Supervision, Project administration, Funding acquisition, Writing—review & editing, Resources.

## CONFLICT OF INTEREST STATEMENT

F.M.L. is listed as an inventor on patents relating to a compound, LM11A‐31, discussed in this report, that is assigned to the University of North Carolina, University of California, and the Department of Veterans Affairs at San Francisco. F.M.L. is entitled to royalties distributed by UC and the VA per their standard agreements. F.M.L. is a founder, equity holder, board member, and paid consultant for PharmatrophiX Inc., a company focused on the development of small‐molecule ligands for neurotrophin receptors that has licensed several of these patents. V.F.L., D.A.S., R.R.B., T.L.M.M., J.Z., H.L., L.M.E., and T.Y. declare no competing interests. Author disclosures are available in the Supporting Information.

## Supporting information



Supporting Information

Supporting Information

Supporting Information

Supporting Information

Supporting Information

Supporting Information

Supporting Information

Supporting Information

## Data Availability

All data generated or analyzed during this study are included in this published article and its supplementary information files. The code used for statistical analysis and figure generation is available at https://github.com/Longo‐Lab/tau_review
