# Supplementary material for: Twenty years of therapeutic development in tauopathy mouse models: a scoping review
Source: Alzheimers Dement. 2025 Aug 18;21(8):e70578. doi: 10.1002/alz.70578 (PMC12360913; doi:10.1002/alz.70578)
Supplement: Supplementary file 1 — Supporting Information [file ALZ-21-e70578-s003.docx]

**Supplemental References 1:** References for Table S1.

[a1] Jiang S, Sydney EJ, Runyan AM, Serpe R, Srikanth M, Figueroa HY, et al. 5-HT4 receptor agonists treatment reduces tau pathology and behavioral deficit in the PS19 mouse model of tauopathy. Front Cell Neurosci. 2024;18:1338502.

[a2] Giannopoulos PF, Chiu J, Pratico D. Antileukotriene therapy by reducing tau phosphorylation improves synaptic integrity and cognition of P301S transgenic mice. Aging Cell. 2018;17(3):e12759.

[a3] Hebron ML, Javidnia M, Moussa CE. Tau clearance improves astrocytic function and brain glutamate-glutamine cycle. J Neurol Sci. 2018;391:90-9.

[a4] Yoshiyama Y, Kojima A, Ishikawa C, Arai K. Anti-inflammatory action of donepezil ameliorates tau pathology, synaptic loss, and neurodegeneration in a tauopathy mouse model. J Alzheimers Dis. 2010;22(1):295-306.

[a5] Zadrozny M, Drapich P, Gasiorowska-Bien A, Niewiadomski W, Harrington CR, Wischik CM, et al. Neuroprotection of Cholinergic Neurons with a Tau Aggregation Inhibitor and Rivastigmine in an Alzheimer's-like Tauopathy Mouse Model. Cells. 2024;13(7).

[a6] Anglada-Huguet M, Endepols H, Sydow A, Hilgers R, Neumaier B, Drzezga A, et al. Reversal of Tau-Dependent Cognitive Decay by Blocking Adenosine A1 Receptors: Comparison of Transgenic Mouse Models with Different Levels of Tauopathy. Int J Mol Sci. 2023;24(11).

[a7] Dennissen FJ, Anglada-Huguet M, Sydow A, Mandelkow E, Mandelkow EM. Adenosine A1 receptor antagonist rolofylline alleviates axonopathy caused by human Tau DeltaK280. Proc Natl Acad Sci U S A. 2016;113(41):11597-602.

[a8] Laurent C, Burnouf S, Ferry B, Batalha VL, Coelho JE, Baqi Y, et al. A2A adenosine receptor deletion is protective in a mouse model of Tauopathy. Mol Psychiatry. 2016;21(1):97-107.

[a9] Laurent C, Eddarkaoui S, Derisbourg M, Leboucher A, Demeyer D, Carrier S, et al. Beneficial effects of caffeine in a transgenic model of Alzheimer's disease-like tau pathology. Neurobiol Aging. 2014;35(9):2079-90.

[a10] Wang C, Chang Y, Zhu J, Wu Y, Jiang X, Zheng S, et al. AdipoRon mitigates tau pathology and restores mitochondrial dynamics via AMPK-related pathway in a mouse model of Alzheimer's disease. Exp Neurol. 2023;363:114355.

[a11] Brendel M, Deussing M, Blume T, Kaiser L, Probst F, Overhoff F, et al. Late-stage Anle138b treatment ameliorates tau pathology and metabolic decline in a mouse model of human Alzheimer's disease tau. Alzheimers Res Ther. 2019;11(1):67.

[a12] Wagner J, Krauss S, Shi S, Ryazanov S, Steffen J, Miklitz C, et al. Reducing tau aggregates with anle138b delays disease progression in a mouse model of tauopathies. Acta Neuropathol. 2015;130(5):619-31.

[a13] Park J, Choi H, Kim YD, Kim SH, Kim Y, Gwon Y, et al. Aberrant role of ALK in tau proteinopathy through autophagosomal dysregulation. Mol Psychiatry. 2021;26(10):5542-56.

[a14] Koppel J, Jimenez H, Adrien L, Greenwald BS, Marambaud P, Cinamon E, et al. Haloperidol inactivates AMPK and reduces tau phosphorylation in a tau mouse model of Alzheimer's disease. Alzheimers Dement (N Y). 2016;2(2):121-30.

[a15] Collu R, Giunti E, Daley S, Chen M, Xia W. Angiotensin-converting enzyme inhibitors and statins therapies-induced changes in omics profiles in humans and transgenic tau mice. Biomed Pharmacother. 2023;168:115756.

[a16] Popelova A, Prazienkova V, Neprasova B, Kasperova BJ, Hruba L, Holubova M, et al. Novel Lipidized Analog of Prolactin-Releasing Peptide Improves Memory Impairment and Attenuates Hyperphosphorylation of Tau Protein in a Mouse Model of Tauopathy. J Alzheimers Dis. 2018;62(4):1725-36.

[a17] Umeda T, Ono K, Sakai A, Yamashita M, Mizuguchi M, Klein WL, et al. Rifampicin is a candidate preventive medicine against amyloid-beta and tau oligomers. Brain. 2016;139(Pt 5):1568-86.

[a18] Zaki MO, El-Desouky S, Elsherbiny DA, Salama M, Azab SS. Glimepiride mitigates tauopathy and neuroinflammation in P301S transgenic mice: role of AKT/GSK3beta signaling. Inflammopharmacology. 2022;30(5):1871-90.

[a19] Nakashima H, Ishihara T, Yokota O, Terada S, Trojanowski JQ, Lee VM, et al. Effects of alpha-tocopherol on an animal model of tauopathies. Free Radic Biol Med. 2004;37(2):176-86.

[a20] Elipenahli C, Stack C, Jainuddin S, Gerges M, Yang L, Starkov A, et al. Behavioral improvement after chronic administration of coenzyme Q10 in P301S transgenic mice. J Alzheimers Dis. 2012;28(1):173-82.

[a21] Kelliny S, Xiong J, Bobrovskaya L, Zhou XF. Preclinical validation of a novel oral Edaravone formulation for treatment of frontotemporal dementia. Neurotox Res. 2021;39(6):1689-707.

[a22] Yu L, Wang W, Pang W, Xiao Z, Jiang Y, Hong Y. Dietary Lycopene Supplementation Improves Cognitive Performances in Tau Transgenic Mice Expressing P301L Mutation via Inhibiting Oxidative Stress and Tau Hyperphosphorylation. J Alzheimers Dis. 2017;57(2):475-82.

[a23] Sun XY, Dong QX, Zhu J, Sun X, Zhang LF, Qiu M, et al. Resveratrol Rescues Tau-Induced Cognitive Deficits and Neuropathology in a Mouse Model of Tauopathy. Curr Alzheimer Res. 2019;16(8):710-22.

[a24] Qian S, Gu J, Dai W, Jin N, Chu D, Huang Q, et al. Sirt1 enhances tau exon 10 inclusion and improves spatial memory of Htau mice. Aging (Albany NY). 2018;10(9):2498-510.

[a25] Hole KL, Staniaszek LE, Menon Balan G, Mason JM, Brown JT, Williams RJ. Oral (-)-Epicatechin Inhibits Progressive Tau Pathology in rTg4510 Mice Independent of Direct Actions at GSK3beta. Front Neurosci. 2021;15:697319.

[a26] Yu KC, Kwan P, Cheung SKK, Ho A, Baum L. Effects of Resveratrol and Morin on Insoluble Tau in Tau Transgenic Mice. Transl Neurosci. 2018;9(1):54-60.

[a27] Zhang YH, Wang DW, Xu SF, Zhang S, Fan YG, Yang YY, et al. alpha-Lipoic acid improves abnormal behavior by mitigation of oxidative stress, inflammation, ferroptosis, and tauopathy in P301S Tau transgenic mice. Redox Biol. 2018b;14:535-48.

[a28] Jones JH, Xin Z, Himmelbauer M, Dechantsreiter M, Enyedy I, Hedde J, et al. Discovery of Potent, Selective, and Brain-Penetrant Apoptosis Signal-Regulating Kinase 1 (ASK1) Inhibitors that Modulate Brain Inflammation In Vivo. J Med Chem. 2021;64(20):15402-19.

[a29] Bold CS, Baltissen D, Ludewig S, Back MK, Just J, Kilian L, et al. APPsalpha Rescues Tau-Induced Synaptic Pathology. J Neurosci. 2022;42(29):5782-802.

[a30] Baltissen D, Bold CS, Rehra L, Banicevic M, Fricke J, Just J, et al. APPsalpha rescues CDK5 and GSK3beta dysregulation and restores normal spine density in Tau transgenic mice. Front Cell Neurosci. 2023;17:1106176.

[a31] Yang C, Dan D, Xu J, Qiu C, He K, Zhang C-E, et al. Arctigenin attenuated spatial memory impairment in pR5 mice by regulating mitochondrial energy metabolism. J Pharm Pharmacol. 2024;76(2):154-61.

[a32] Qian Z, Li B, Meng X, Liao J, Wang G, Li Y, et al. Inhibition of asparagine endopeptidase (AEP) effectively treats sporadic Alzheimer's disease in mice. Neuropsychopharmacology. 2023.

[a33] Zhang Z, Obianyo O, Dall E, Du Y, Fu H, Liu X, et al. Inhibition of delta-secretase improves cognitive functions in mouse models of Alzheimer's disease. Nat Commun. 2017;8:14740.

[a34] Wang ZH, Wu W, Kang SS, Liu X, Wu Z, Peng J, et al. BDNF inhibits neurodegenerative disease-associated asparaginyl endopeptidase activity via phosphorylation by AKT. JCI Insight. 2018b;3(16).

[a35] Guo T, Pan X, Jiang G, Zhang D, Qi J, Shao L, et al. Downregulating PTBP1 fails to convert astrocytes into hippocampal neurons and to alleviate symptoms in Alzheimer's mouse models. J Neurosci. 2022;42(38):7309-17.

[a36] Shimada K, Motoi Y, Ishiguro K, Kambe T, Matsumoto SE, Itaya M, et al. Long-term oral lithium treatment attenuates motor disturbance in tauopathy model mice: implications of autophagy promotion. Neurobiol Dis. 2012;46(1):101-8.

[a37] Schaeffer V, Lavenir I, Ozcelik S, Tolnay M, Winkler DT, Goedert M. Stimulation of autophagy reduces neurodegeneration in a mouse model of human tauopathy. Brain. 2012;135(Pt 7):2169-77.

[a38] Zhang L, Hou S, Movahedi F, Li Z, Li L, Hu J, et al. Amyloid-β/Tau burden and neuroinflammation dual-targeted nanomedicines synergistically restore memory and recognition of Alzheimer’s disease mice. Nano Today. 2023;49.

[a39] Jiao SS, Shen LL, Zhu C, Bu XL, Liu YH, Liu CH, et al. Brain-derived neurotrophic factor protects against tau-related neurodegeneration of Alzheimer's disease. Transl Psychiatry. 2016;6(10):e907.

[a40] Ano Y, Takaichi Y, Ohya R, Uchida K, Nakayama H, Takashima A. Tryptophan-tyrosine dipeptide improves tau-related symptoms in tauopathy mice. Nutr Neurosci. 2022:1-12.

[a41] Thomas S, Hoxha K, Tran A, Prendergast GC. Bin1 antibody lowers the expression of phosphorylated Tau in Alzheimer's disease. J Cell Biochem. 2019;120(10):18320-31.

[a42] Lee HJ, Jeon SG, Kim J, Kang RJ, Kim SM, Han KM, et al. Ibrutinib modulates Abeta/tau pathology, neuroinflammation, and cognitive function in mouse models of Alzheimer's disease. Aging Cell. 2021;20(3):e13332.

[a43] Kreilaus F, Przybyla M, Ittner L, Karl T. Cannabidiol (CBD) treatment improves spatial memory in 14-month-old female TAU58/2 transgenic mice. Behav Brain Res. 2022;425:113812.

[a44] Watt G, Chesworth R, Przybyla M, Ittner A, Garner B, Ittner LM, et al. Chronic cannabidiol (CBD) treatment did not exhibit beneficial effects in 4-month-old male TAU58/2 transgenic mice. Pharmacol Biochem Behav. 2020;196:172970.

[a45] Chatterjee S, Cassel R, Schneider-Anthony A, Merienne K, Cosquer B, Tzeplaeff L, et al. Reinstating plasticity and memory in a tauopathy mouse model with an acetyltransferase activator. EMBO Mol Med. 2018;10(11):e8587.

[a46] Min SW, Chen X, Tracy TE, Li Y, Zhou Y, Wang C, et al. Critical role of acetylation in tau-mediated neurodegeneration and cognitive deficits. Nat Med. 2015;21(10):1154-62.

[a47] Lee HJ, Hoe HS. Inhibition of CDK4/6 regulates AD pathology, neuroinflammation and cognitive function through DYRK1A/STAT3 signaling. Pharmacol Res. 2023;190:106725.

[a48] Hampton DW, Webber DJ, Bilican B, Goedert M, Spillantini MG, Chandran S. Cell-mediated neuroprotection in a mouse model of human tauopathy. J Neurosci. 2010;30(30):9973-83.

[a49] Zhang HA, Yuan CX, Liu KF, Yang QF, Zhao J, Li H, et al. Neural stem cell transplantation alleviates functional cognitive deficits in a mouse model of tauopathy. Neural Regen Res. 2022;17(1):152-62.

[a50] Spillantini MG, Iovino M, Vuono R. Release of growth factors by neuronal precursor cells as a treatment for diseases with tau pathology. Arch Ital Biol. 2011;149(2):215-23.

[a51] Boimel M, Grigoriadis N, Lourbopoulos A, Touloumi O, Rosenmann D, Abramsky O, et al. Statins reduce the neurofibrillary tangle burden in a mouse model of tauopathy. J Neuropathol Exp Neurol. 2009;68(3):314-25.

[a52] Yang S, Hilton S, Alves JN, Saksida LM, Bussey T, Matthews RT, et al. Antibody recognizing 4-sulfated chondroitin sulfate proteoglycans restores memory in tauopathy-induced neurodegeneration. Neurobiol Aging. 2017;59:197-209.

[a53] Yang S, Cacquevel M, Saksida LM, Bussey TJ, Schneider BL, Aebischer P, et al. Perineuronal net digestion with chondroitinase restores memory in mice with tau pathology. Exp Neurol. 2015;265:48-58.

[a54] Dave N, Vural AS, Piras IS, Winslow W, Surendra L, Winstone JK, et al. Identification of retinoblastoma binding protein 7 (Rbbp7) as a mediator against tau acetylation and subsequent neuronal loss in Alzheimer's disease and related tauopathies. Acta Neuropathol. 2021;142(2):279-94.

[a55] Yao Y, Chang Y, Li S, Zhu J, Wu Y, Jiang X, et al. Complement C3a receptor antagonist alleviates tau pathology and ameliorates cognitive deficits in P301S mice. Brain Res Bull. 2023;200:110685.

[a56] Dejanovic B, Huntley MA, De Maziere A, Meilandt WJ, Wu T, Srinivasan K, et al. Changes in the Synaptic Proteome in Tauopathy and Rescue of Tau-Induced Synapse Loss by C1q Antibodies. Neuron. 2018;100(6):1322-36 e7.

[a57] Ma D, Huang R, Guo K, Zhao Z, Wei W, Gu L, et al. Cornel Iridoid Glycoside Protects Against STAT1-Dependent Synapse and Memory Deficits by Increasing N-Methyl-D-aspartate Receptor Expression in a Tau Transgenic Mice. Front Aging Neurosci. 2021;13:671206.

[a58] Yang CC, Zheng CC, Luo Y, Guo KW, Gao D, Zhang L, et al. Cornel Iridoid Glycoside and Its Effective Component Regulate ATPase Vps4A/JNK to Alleviate Autophagy Deficit with Autophagosome Accumulation. Am J Chin Med. 2022;50(6):1599-615.

[a59] Guo K, Yang C, Zhang L. The Reduction of Tau Hyperphosphorylation by Cornel Iridoid Glycosides Is Mediated by Their Influence on Calpain Activity. Evid Based Complement Alternat Med. 2022;2022:9213046.

[a60] Ma D, Luo Y, Huang R, Zhao Z, Wang Q, Li L, et al. Cornel Iridoid Glycoside Suppresses Tau Hyperphosphorylation and Aggregation in a Mouse Model of Tauopathy through Increasing Activity of PP2A. Curr Alzheimer Res. 2019;16(14):1316-31.

[a61] Ma DL, Luo Y, Huang R, Zhao ZR, Zhang L, Li YL, et al. Cornel Iridoid Glycoside Suppresses Hyperactivity Phenotype in rTg4510 Mice through Reducing Tau Pathology and Improving Synaptic Dysfunction. Curr Med Sci. 2020;40(6):1031-9.

[a62] Zhu XC, Liu L, Dai WZ, Ma T. Crry silencing alleviates Alzheimer's disease injury by regulating neuroinflammatory cytokines and the complement system. Neural Regen Res. 2022;17(8):1841-9.

[a63] Mancuso R, Fryatt G, Cleal M, Obst J, Pipi E, Monzon-Sandoval J, et al. CSF1R inhibitor JNJ-40346527 attenuates microglial proliferation and neurodegeneration in P301S mice. Brain. 2019;142(10):3243-64.

[a64] Sedjahtera A, Gunawan L, Bray L, Hung LW, Parsons J, Okamura N, et al. Targeting metals rescues the phenotype in an animal model of tauopathy. Metallomics. 2018;10(9):1339-47.

[a65] Ma QL, Zuo X, Yang F, Ubeda OJ, Gant DJ, Alaverdyan M, et al. Curcumin suppresses soluble tau dimers and corrects molecular chaperone, synaptic, and behavioral deficits in aged human tau transgenic mice. J Biol Chem. 2013;288(6):4056-65.

[a66] Yanagisawa D, Hamezah HS, Durani LW, Taguchi H, Tooyama I. Study of tau pathology in male rTg4510 mice fed with a curcumin derivative Shiga-Y5. PLoS One. 2018;13(12):e0208440.

[a67] Burlot MA, Braudeau J, Michaelsen-Preusse K, Potier B, Ayciriex S, Varin J, et al. Cholesterol 24-hydroxylase defect is implicated in memory impairments associated with Alzheimer-like Tau pathology. Hum Mol Genet. 2015;24(21):5965-76.

[a68] Vera R, Hong N, Jiang B, Liang G, Eckenhoff MF, Kincaid HJ, et al. Effects of Intranasal Dantrolene Nanoparticles on Brain Concentration and Behavior in PS19 Tau Transgenic Mice. J Alzheimers Dis. 2024;98(2):549-62.

[a69] Riordan R, Rong W, Yu Z, Ross G, Valerio J, Dimas-Munoz J, et al. Effect of Nrf2 loss on senescence and cognition of tau-based P301S mice. Geroscience. 2023.

[a70] Makani V, Zhang B, Han H, Yao Y, Lassalas P, Lou K, et al. Evaluation of the brain-penetrant microtubule-stabilizing agent, dictyostatin, in the PS19 tau transgenic mouse model of tauopathy. Acta Neuropathol Commun. 2016;4(1):106.

[a71] Brownlow ML, Joly-Amado A, Azam S, Elza M, Selenica ML, Pappas C, et al. Partial rescue of memory deficits induced by calorie restriction in a mouse model of tau deposition. Behav Brain Res. 2014;271:79-88.

[a72] Cogut V, McNeely TL, Bussian TJ, Graves SI, Baker DJ. Caloric Restriction Improves Spatial Learning Deficits in Tau Mice. J Alzheimers Dis. 2024;98(3):925-40.

[a73] Brownlow ML, Benner L, D'Agostino D, Gordon MN, Morgan D. Ketogenic diet improves motor performance but not cognition in two mouse models of Alzheimer's pathology. PLoS One. 2013;8(9):e75713.

[a74] Buccarello L, Grignaschi G, Di Giancamillo A, Domeneghini C, Melcangi RC, Borsello T. Neuroprotective effects of low fat-protein diet in the P301L mouse model of tauopathy. Neuroscience. 2017;354:208-20.

[a75] van Hummel A, Taleski G, Sontag JM, Feiten AF, Ke YD, Ittner LM, et al. Methyl donor supplementation reduces phospho-Tau, Fyn and demethylated protein phosphatase 2A levels and mitigates learning and motor deficits in a mouse model of tauopathy. Neuropathology and Applied Neurobiology. 2023;49(4).

[a76] Peters OM, Connor-Robson N, Sokolov VB, Aksinenko AY, Kukharsky MS, Bachurin SO, et al. Chronic administration of dimebon ameliorates pathology in TauP301S transgenic mice. J Alzheimers Dis. 2013;33(4):1041-9.

[a77] Chang Y, Yao Y, Ma R, Wang Z, Hu J, Wu Y, et al. Dl-3-n-Butylphthalide Reduces Cognitive Deficits and Alleviates Neuropathology in P301S Tau Transgenic Mice. Front Neurosci. 2021;15:620176.

[a78] Kam K, Vetter K, Tejiram RA, Pettibone WD, Shim K, Audrain M, et al. Effect of aging and a dual orexin receptor antagonist on sleep architecture and NREM oscillations including a REM Behavior Disorder phenotype in the PS19 mouse model of tauopathy. J Neurosci. 2023.

[a79] Keenan RJ, Daykin H, Chu J, Cornthwaite-Duncan L, Allocca G, Hoyer D, et al. Differential sleep/wake response and sex differences following acute suvorexant, MK-1064 and zolpidem administration in the rTg4510 mouse model of tauopathy. Br J Pharmacol. 2022;179(13):3403-17.

[a80] Keenan RJ, Daykin H, Metha J, Cornthwaite-Duncan L, Wright DK, Clarke K, et al. Orexin 2 receptor antagonism sex-dependently improves sleep/wakefulness and cognitive performance in tau transgenic mice. Br J Pharmacol. 2024;181(1):87-106.

[a81] Melchior B, Mittapalli GK, Lai C, Duong-Polk K, Stewart J, Guner B, et al. Tau pathology reduction with SM07883, a novel, potent, and selective oral DYRK1A inhibitor: A potential therapeutic for Alzheimer's disease. Aging Cell. 2019;18(5):e13000.

[a82] Foster JB, Lashley R, Zhao F, Wang X, Kung N, Askwith CC, et al. Enhancement of tripartite synapses as a potential therapeutic strategy for Alzheimer's disease: a preclinical study in rTg4510 mice. Alzheimers Res Ther. 2019;11(1):75.

[a83] Kim J, Kim SJ, Jeong HR, Park JH, Moon M, Hoe HS. Inhibiting EGFR/HER-2 ameliorates neuroinflammatory responses and the early stage of tau pathology through DYRK1A. Front Immunol. 2022;13:903309.

[a84] Wang W, Cao Q, Tan T, Yang F, Williams JB, Yan Z. Epigenetic treatment of behavioral and physiological deficits in a tauopathy mouse model. Aging Cell. 2021;20(10):e13456.

[a85] Barten DM, Fanara P, Andorfer C, Hoque N, Wong PY, Husted KH, et al. Hyperdynamic microtubules, cognitive deficits, and pathology are improved in tau transgenic mice with low doses of the microtubule-stabilizing agent BMS-241027. J Neurosci. 2012;32(21):7137-45.

[a86] Brunden KR, Zhang B, Carroll J, Yao Y, Potuzak JS, Hogan AM, et al. Epothilone D improves microtubule density, axonal integrity, and cognition in a transgenic mouse model of tauopathy. J Neurosci. 2010;30(41):13861-6.

[a87] Robles-Gómez Á A, Ordaz B, Lorea-Hernández JJ, Peña-Ortega F. Deleterious and protective effects of epothilone-D alone and in the context of amyloid β- and tau-induced alterations. Front Mol Neurosci. 2023;16:1198299.

[a88] Zhang B, Carroll J, Trojanowski JQ, Yao Y, Iba M, Potuzak JS, et al. The microtubule-stabilizing agent, epothilone D, reduces axonal dysfunction, neurotoxicity, cognitive deficits, and Alzheimer-like pathology in an interventional study with aged tau transgenic mice. J Neurosci. 2012;32(11):3601-11.

[a89] Le Corre S, Klafki HW, Plesnila N, Hubinger G, Obermeier A, Sahagun H, et al. An inhibitor of tau hyperphosphorylation prevents severe motor impairments in tau transgenic mice. Proc Natl Acad Sci U S A. 2006;103(25):9673-8.

[a90] Choi YB, Dunn-Meynell AA, Marchese M, Blumberg BM, Gaindh D, Dowling PC, et al. Erythropoietin-derived peptide treatment reduced neurological deficit and neuropathological changes in a mouse model of tauopathy. Alzheimers Res Ther. 2021;13(1):32.

[a91] Yang J, Ou W, Jagadeesan N, Simanauskaite J, Sun J, Castellanos D, et al. The Effects of a Blood-Brain Barrier Penetrating Erythropoietin in a Mouse Model of Tauopathy. Pharmaceuticals (Basel). 2023;16(4).

[a92] Belarbi K, Burnouf S, Fernandez-Gomez FJ, Laurent C, Lestavel S, Figeac M, et al. Beneficial effects of exercise in a transgenic mouse model of Alzheimer's disease-like Tau pathology. Neurobiol Dis. 2011;43(2):486-94.

[a93] Leem YH, Lee YI, Son HJ, Lee SH. Chronic exercise ameliorates the neuroinflammation in mice carrying NSE/htau23. Biochem Biophys Res Commun. 2011;406(3):359-65.

[a94] He K, Nie L, Yang C, Liu Z, Huang X, Li S, et al. Exhaustive exercise decreases tau phosphorylation and modifies biological processes associated with the protein translation and electron transport chain in P301L tau transgenic mice. Exp Gerontol. 2024;187:112375.

[a95] Ohia-Nwoko O, Montazari S, Lau YS, Eriksen JL. Long-term treadmill exercise attenuates tau pathology in P301S tau transgenic mice. Mol Neurodegener. 2014;9(1):54.

[a96] Lahiani-Cohen I, Lourbopoulos A, Haber E, Rozenstein-Tsalkovich L, Abramsky O, Grigoriadis N, et al. Moderate environmental enrichment mitigates tauopathy in a neurofibrillary tangle mouse model. J Neuropathol Exp Neurol. 2011;70(7):610-21.

[a97] Lauretti E, Nenov M, Dincer O, Iuliano L, Pratico D. Extra virgin olive oil improves synaptic activity, short-term plasticity, memory, and neuropathology in a tauopathy model. Aging Cell. 2020;19(1):e13076.

[a98] Martin SC, Joyce KK, Harper KM, Harp SJ, Cohen TJ, Moy SS, et al. Evaluating Fatty Acid Amide Hydrolase as a Suitable Target for Sleep Promotion in a Transgenic TauP301S Mouse Model of Neurodegeneration. Pharmaceuticals (Basel). 2024;17(3).

[a99] Hernandez I, Luna G, Rauch JN, Reis SA, Giroux M, Karch CM, et al. A farnesyltransferase inhibitor activates lysosomes and reduces tau pathology in mice with tauopathy. Sci Transl Med. 2019;11(485).

[a100] Glat M, Skaat H, Menkes-Caspi N, Margel S, Stern EA. Age-dependent effects of microglial inhibition in vivo on Alzheimer's disease neuropathology using bioactive-conjugated iron oxide nanoparticles. J Nanobiotechnology. 2013;11(1):32.

[a101] Finneran DJ, Morgan D, Gordon MN, Nash KR. CNS-Wide over Expression of Fractalkine Improves Cognitive Functioning in a Tauopathy Model. J Neuroimmune Pharmacol. 2019;14(2):312-25.

[a102] Nash KR, Lee DC, Hunt JB, Jr., Morganti JM, Selenica ML, Moran P, et al. Fractalkine overexpression suppresses tau pathology in a mouse model of tauopathy. Neurobiol Aging. 2013;34(6):1540-8.

[a103] Tang SJ, Fesharaki-Zadeh A, Takahashi H, Nies SH, Smith LM, Luo A, et al. Fyn kinase inhibition reduces protein aggregation, increases synapse density and improves memory in transgenic and traumatic Tauopathy. Acta Neuropathol Commun. 2020;8(1):96.

[a104] Xu NZ, Ernst M, Treven M, Cerne R, Wakulchik M, Li X, et al. Negative allosteric modulation of alpha 5-containing GABA(A) receptors engenders antidepressant-like effects and selectively prevents age-associated hyperactivity in tau-depositing mice. Psychopharmacology (Berl). 2018;235(4):1151-61.

[a105] Levenson JM, Schroeter S, Carroll JC, Cullen V, Asp E, Proschitsky M, et al. NPT088 reduces both amyloid-beta and tau pathologies in transgenic mice. Alzheimers Dement (N Y). 2016;2(3):141-55.

[a106] Adaikkan C, Middleton SJ, Marco A, Pao PC, Mathys H, Kim DN, et al. Gamma Entrainment Binds Higher-Order Brain Regions and Offers Neuroprotection. Neuron. 2019;102(5):929-43 e8.

[a107] Martorell AJ, Paulson AL, Suk HJ, Abdurrob F, Drummond GT, Guan W, et al. Multi-sensory Gamma Stimulation Ameliorates Alzheimer's-Associated Pathology and Improves Cognition. Cell. 2019;177(2):256-71 e22.

[a108] Qin Y, Zhang Y, Tomic I, Hao W, Menger MD, Liu C, et al. Ginkgo biloba Extract EGb 761 and Its Specific Components Elicit Protective Protein Clearance Through the Autophagy-Lysosomal Pathway in Tau-Transgenic Mice and Cultured Neurons. J Alzheimers Dis. 2018;65(1):243-63.

[a109] Ries M, Watts H, Mota BC, Lopez MY, Donat CK, Baxan N, et al. Annexin A1 restores cerebrovascular integrity concomitant with reduced amyloid-beta and tau pathology. Brain. 2021;144(5):1526-41.

[a110] Hunsberger HC, Weitzner DS, Rudy CC, Hickman JE, Libell EM, Speer RR, et al. Riluzole rescues glutamate alterations, cognitive deficits, and tau pathology associated with P301L tau expression. J Neurochem. 2015;135(2):381-94.

[a111] Hunsberger HC, Hickman JE, Reed MN. Riluzole rescues alterations in rapid glutamate transients in the hippocampus of rTg4510 mice. Metab Brain Dis. 2016;31(3):711-5.

[a112] Santa-Maria I, Diaz-Ruiz C, Ksiezak-Reding H, Chen A, Ho L, Wang J, et al. GSPE interferes with tau aggregation in vivo: implication for treating tauopathy. Neurobiol Aging. 2012;33(9):2072-81.

[a113] Wang J, Santa-Maria I, Ho L, Ksiezak-Reding H, Ono K, Teplow DB, et al. Grape derived polyphenols attenuate tau neuropathology in a mouse model of Alzheimer's disease. J Alzheimers Dis. 2010;22(2):653-61.

[a114] Uno Y, Iwashita H, Tsukamoto T, Uchiyama N, Kawamoto T, Kori M, et al. Efficacy of a novel, orally active GSK-3 inhibitor 6-Methyl-N-[3-[[3-(1-methylethoxy)propyl]carbamoyl]-1H-pyrazol-4-yl]pyridine-3-carboxamide in tau transgenic mice. Brain Res. 2009;1296:148-63.

[a115] Griebel G, Stemmelin J, Lopez-Grancha M, Boulay D, Boquet G, Slowinski F, et al. The selective GSK3 inhibitor, SAR502250, displays neuroprotective activity and attenuates behavioral impairments in models of neuropsychiatric symptoms of Alzheimer's disease in rodents. Sci Rep. 2019;9(1):18045.

[a116] Sreenivasmurthy SG, Iyaswamy A, Krishnamoorthi S, Reddi RN, Kammala AK, Vasudevan K, et al. Bromo-protopine, a novel protopine derivative, alleviates tau pathology by activating chaperone-mediated autophagy for Alzheimer's disease therapy. Front Mol Biosci. 2022a;9:1030534.

[a117] Sreenivasmurthy SG, Iyaswamy A, Krishnamoorthi S, Senapati S, Malampati S, Zhu Z, et al. Protopine promotes the proteasomal degradation of pathological tau in Alzheimer's disease models via HDAC6 inhibition. Phytomedicine. 2022b;96:153887.

[a118] Onishi T, Maeda R, Terada M, Sato S, Fujii T, Ito M, et al. A novel orally active HDAC6 inhibitor T-518 shows a therapeutic potential for Alzheimer's disease and tauopathy in mice. Sci Rep. 2021;11(1):15423.

[a119] Selenica ML, Benner L, Housley SB, Manchec B, Lee DC, Nash KR, et al. Histone deacetylase 6 inhibition improves memory and reduces total tau levels in a mouse model of tau deposition. Alzheimers Res Ther. 2014a;6(1):12.

[a120] Valencia A, Bieber VLR, Bajrami B, Marsh G, Hamann S, Wei R, et al. Antisense Oligonucleotide-Mediated Reduction of HDAC6 Does Not Reduce Tau Pathology in P301S Tau Transgenic Mice. Front Neurol. 2021;12:624051.

[a121] Bondulich MK, Guo T, Meehan C, Manion J, Rodriguez Martin T, Mitchell JC, et al. Tauopathy induced by low level expression of a human brain-derived tau fragment in mice is rescued by phenylbutyrate. Brain. 2016;139(Pt 8):2290-306.

[a122] Rodriguez Ospina S, Blazier DM, Criado-Marrero M, Gould LA, Gebru NT, Beaulieu-Abdelahad D, et al. Small Heat Shock Protein 22 Improves Cognition and Learning in the Tauopathic Brain. Int J Mol Sci. 2022;23(2).

[a123] Hampton DW, Amor S, Story D, Torvell M, Bsibsi M, van Noort JM, et al. HspB5 Activates a Neuroprotective Glial Cell Response in Experimental Tauopathy. Front Neurosci. 2020;14:574.

[a124] Delay-Goyet P, Blanchard V, Schussler N, Lopez-Grancha M, Menager J, Mary V, et al. SAR110894, a potent histamine H3-receptor antagonist, displays disease-modifying activity in a transgenic mouse model of tauopathy. Alzheimers Dement (N Y). 2016;2(4):267-80.

[a125] Gaikwad S, Puangmalai N, Bittar A, Montalbano M, Garcia S, McAllen S, et al. Tau oligomer induced HMGB1 release contributes to cellular senescence and neuropathology linked to Alzheimer's disease and frontotemporal dementia. Cell Rep. 2021;36(3):109419.

[a126] Ho SW, Tsui YT, Wong TT, Cheung SK, Goggins WB, Yi LM, et al. Effects of 17-allylamino-17-demethoxygeldanamycin (17-AAG) in transgenic mouse models of frontotemporal lobar degeneration and Alzheimer's disease. Transl Neurodegener. 2013;2(1):24.

[a127] Thirstrup K, Sotty F, Montezinho LC, Badolo L, Thougaard A, Kristjansson M, et al. Linking HSP90 target occupancy to HSP70 induction and efficacy in mouse brain. Pharmacol Res. 2016;104:197-205.

[a128] Dickey CA, Kamal A, Lundgren K, Klosak N, Bailey RM, Dunmore J, et al. The high-affinity HSP90-CHIP complex recognizes and selectively degrades phosphorylated tau client proteins. J Clin Invest. 2007;117(3):648-58.

[a129] Ising C, Gallardo G, Leyns CEG, Wong CH, Jiang H, Stewart F, et al. AAV-mediated expression of anti-tau scFvs decreases tau accumulation in a mouse model of tauopathy. J Exp Med. 2017;214(5):1227-38.

[a130] Yanamandra K, Kfoury N, Jiang H, Mahan TE, Ma S, Maloney SE, et al. Anti-tau antibodies that block tau aggregate seeding in vitro markedly decrease pathology and improve cognition in vivo. Neuron. 2013;80(2):402-14.

[a131] Yanamandra K, Jiang H, Mahan TE, Maloney SE, Wozniak DF, Diamond MI, et al. Anti-tau antibody reduces insoluble tau and decreases brain atrophy. Ann Clin Transl Neurol. 2015;2(3):278-88.

[a132] Yanamandra K, Patel TK, Jiang H, Schindler S, Ulrich JD, Boxer AL, et al. Anti-tau antibody administration increases plasma tau in transgenic mice and patients with tauopathy. Sci Transl Med. 2017;9(386):eaal2029.

[a133] Yang YY, Ren YT, Jia MY, Bai CY, Liang XT, Gao HL, et al. The human islet amyloid polypeptide reduces hippocampal tauopathy and behavioral impairments in P301S mice without inducing neurotoxicity or seeding amyloid aggregation. Exp Neurol. 2023b;362:114346.

[a134] Yoshiyama Y, Higuchi M, Zhang B, Huang SM, Iwata N, Saido TC, et al. Synapse loss and microglial activation precede tangles in a P301S tauopathy mouse model. Neuron. 2007;53(3):337-51.

[a135] Briggs DI, Defensor E, Memar Ardestani P, Yi B, Halpain M, Seabrook G, et al. Role of Endoplasmic Reticulum Stress in Learning and Memory Impairment and Alzheimer's Disease-Like Neuropathology in the PS19 and APP(Swe) Mouse Models of Tauopathy and Amyloidosis. eNeuro. 2017;4(4).

[a136] Bretland KA, Lin L, Bretland KM, Smith MA, Fleming SM, Dengler-Crish CM. Irisin treatment lowers levels of phosphorylated tau in the hippocampus of pre-symptomatic female but not male htau mice. Neuropathol Appl Neurobiol. 2021;47(7):967-78.

[a137] Wang C, Wang X, Sun S, Chang Y, Lian P, Guo H, et al. Irisin inhibits microglial senescence via TFAM-mediated mitochondrial metabolism in a mouse model of tauopathy. Immun Ageing. 2024;21(1):30.

[a138] Ano Y, Takaichi Y, Uchida K, Kondo K, Nakayama H, Takashima A. Iso-alpha-Acids, the Bitter Components of Beer, Suppress Microglial Inflammation in rTg4510 Tauopathy. Molecules. 2018;23(12).

[a139] Rodriguez MN, Lippi SLP. Lion's Mane (Hericium erinaceus) Exerts Anxiolytic Effects in the rTg4510 Tau Mouse Model. Behav Sci (Basel). 2022;12(7).

[a140] Chen D, Lan G, Li R, Mei Y, Shui X, Gu X, et al. Melatonin ameliorates tau-related pathology via the miR-504-3p and CDK5 axis in Alzheimer's disease. Transl Neurodegener. 2022;11(1):27.

[a141] Harris CJ, Gray NE, Caruso M, Hunter M, Ralle M, Quinn JF. Copper Modulation and Memory Impairment due to Hippocampal Tau Pathology. J Alzheimers Dis. 2020;78(1):49-60.

[a142] Kwan P, Ho A, Baum L. Effects of Deferasirox in Alzheimer's Disease and Tauopathy Animal Models. Biomolecules. 2022;12(3).

[a143] Fine JM, Baillargeon AM, Renner DB, Hoerster NS, Tokarev J, Colton S, et al. Intranasal deferoxamine improves performance in radial arm water maze, stabilizes HIF-1alpha, and phosphorylates GSK3beta in P301L tau transgenic mice. Exp Brain Res. 2012;219(3):381-90.

[a144] Barini E, Antico O, Zhao Y, Asta F, Tucci V, Catelani T, et al. Metformin promotes tau aggregation and exacerbates abnormal behavior in a mouse model of tauopathy. Mol Neurodegener. 2016;11(1):16.

[a145] Schwab K, Melis V, Harrington CR, Wischik CM, Magbagbeolu M, Theuring F, et al. Proteomic Analysis of Hydromethylthionine in the Line 66 Model of Frontotemporal Dementia Demonstrates Actions on Tau-Dependent and Tau-Independent Networks. Cells. 2021;10(8).

[a146] Melis V, Magbagbeolu M, Rickard JE, Horsley D, Davidson K, Harrington KA, et al. Effects of oxidized and reduced forms of methylthioninium in two transgenic mouse tauopathy models. Behav Pharmacol. 2015;26(4):353-68.

[a147] Congdon EE, Wu JW, Myeku N, Figueroa YH, Herman M, Marinec PS, et al. Methylthioninium chloride (methylene blue) induces autophagy and attenuates tauopathy in vitro and in vivo. Autophagy. 2012;8(4):609-22.

[a148] Hochgrafe K, Sydow A, Matenia D, Cadinu D, Konen S, Petrova O, et al. Preventive methylene blue treatment preserves cognition in mice expressing full-length pro-aggregant human Tau. Acta Neuropathol Commun. 2015;3:25.

[a149] Hosokawa M, Arai T, Masuda-Suzukake M, Nonaka T, Yamashita M, Akiyama H, et al. Methylene blue reduced abnormal tau accumulation in P301L tau transgenic mice. PLoS One. 2012;7(12):e52389.

[a150] Spires-Jones TL, Friedman T, Pitstick R, Polydoro M, Roe A, Carlson GA, et al. Methylene blue does not reverse existing neurofibrillary tangle pathology in the rTg4510 mouse model of tauopathy. Neurosci Lett. 2014;562:63-8.

[a151] Stack C, Jainuddin S, Elipenahli C, Gerges M, Starkova N, Starkov AA, et al. Methylene blue upregulates Nrf2/ARE genes and prevents tau-related neurotoxicity. Hum Mol Genet. 2014;23(14):3716-32.

[a152] Perez-Garcia G, Bicak M, Haure-Mirande JV, Perez GM, Otero-Pagan A, Gama Sosa MA, et al. BCI-838, an orally active mGluR2/3 receptor antagonist pro-drug, rescues learning behavior deficits in the PS19 MAPT(P301S) mouse model of tauopathy. Neurosci Lett. 2023;797:137080.

[a153] Avdeeva NV. Novel mGluR4 agonist Rapitalam ameliorates motor dysfunction in mice with tau-associated neurodegeneration. Research Results in Pharmacology. 2020;6(2):9-17.

[a154] Bennett RE, Bryant A, Hu M, Robbins AB, Hopp SC, Hyman BT. Partial reduction of microglia does not affect tau pathology in aged mice. J Neuroinflammation. 2018;15(1):311.

[a155] El Fatimy R, Li S, Chen Z, Mushannen T, Gongala S, Wei Z, et al. MicroRNA-132 provides neuroprotection for tauopathies via multiple signaling pathways. Acta Neuropathol. 2018;136(4):537-55.

[a156] Guisle I, Canet G, Petry S, Fereydouni-Forouzandeh P, Morin F, Kerauden R, et al. Sauna-like conditions or menthol treatment reduce tau phosphorylation through mild hyperthermia. Neurobiol Aging. 2022;113:118-30.

[a157] Garwood CJ, Cooper JD, Hanger DP, Noble W. Anti-inflammatory impact of minocycline in a mouse model of tauopathy. Front Psychiatry. 2010;1:136.

[a158] Noble W, Garwood C, Stephenson J, Kinsey AM, Hanger DP, Anderton BH. Minocycline reduces the development of abnormal tau species in models of Alzheimer's disease. FASEB J. 2009;23(3):739-50.

[a159] Di J, Siddique I, Li Z, Malki G, Hornung S, Dutta S, et al. The molecular tweezer CLR01 improves behavioral deficits and reduces tau pathology in P301S-tau transgenic mice. Alzheimers Res Ther. 2021;13(1):6.

[a160] Hashem J, Hu M, Zhang J, Gao F, Chen C. Inhibition of 2-Arachidonoylglycerol Metabolism Alleviates Neuropathology and Improves Cognitive Function in a Tau Mouse Model of Alzheimer's Disease. Mol Neurobiol. 2021;58(8):4122-33.

[a161] Chang C-J, Taoufiq Z, Yamada H, Takei K, Tomiyama T, Umeda T, et al. The microtubule-dynamin binding inhibitor peptide PHDP5 rescues spatial learning and memory deficits in Alzheimer's disease model mice. Brain Res. 2024;1838(148987):148987.

[a162] Jiang T, Yu JT, Zhu XC, Zhang QQ, Cao L, Wang HF, et al. Temsirolimus attenuates tauopathy in vitro and in vivo by targeting tau hyperphosphorylation and autophagic clearance. Neuropharmacology. 2014;85:121-30.

[a163] Kim YD, Jeong EI, Nah J, Yoo SM, Lee WJ, Kim Y, et al. Pimozide reduces toxic forms of tau in TauC3 mice via 5' adenosine monophosphate-activated protein kinase-mediated autophagy. J Neurochem. 2017;142(5):734-46.

[a164] Morawe MP, Liao F, Amberg W, van Bergeijk J, Chang R, Gulino M, et al. Pharmacological mTOR-inhibition facilitates clearance of AD-related tau aggregates in the mouse brain. Eur J Pharmacol. 2022;934:175301.

[a165] Ozcelik S, Fraser G, Castets P, Schaeffer V, Skachokova Z, Breu K, et al. Rapamycin attenuates the progression of tau pathology in P301S tau transgenic mice. PLoS One. 2013;8(5):e62459.

[a166] Jimenez H, Carrion J, Adrien L, Wolin A, Eun J, Cinamon E, et al. The Impact of Muscarinic Antagonism on Psychosis-Relevant Behaviors and Striatal [(11)C] Raclopride Binding in Tau Mouse Models of Alzheimer's Disease. Biomedicines. 2023;11(8).

[a167] Song HL, Demirev AV, Kim NY, Kim DH, Yoon SY. Ouabain activates transcription factor EB and exerts neuroprotection in models of Alzheimer's disease. Mol Cell Neurosci. 2019;95:13-24.

[a168] Ljungberg MC, Ali YO, Zhu J, Wu CS, Oka K, Zhai RG, et al. CREB-activity and nmnat2 transcription are down-regulated prior to neurodegeneration, while NMNAT2 over-expression is neuroprotective, in a mouse model of human tauopathy. Hum Mol Genet. 2012;21(2):251-67.

[a169] Vimal SK, Zuo H, Wang Z, Wang H, Long Z, Bhattacharyya S. Self-Therapeutic Nanoparticle That Alters Tau Protein and Ameliorates Tauopathy Toward a Functional Nanomedicine to Tackle Alzheimer's. Small. 2020;16(16):e1906861.

[a170] Comerota MM, Tumurbaatar B, Krishnan B, Kayed R, Taglialatela G. Near Infrared Light Treatment Reduces Synaptic Levels of Toxic Tau Oligomers in Two Transgenic Mouse Models of Human Tauopathies. Mol Neurobiol. 2019;56(5):3341-55.

[a171] Purushothuman S, Nandasena C, Johnstone DM, Stone J, Mitrofanis J. The impact of near-infrared light on dopaminergic cell survival in a transgenic mouse model of parkinsonism. Brain Res. 2013;1535:61-70.

[a172] Purushothuman S, Johnstone DM, Nandasena C, Mitrofanis J, Stone J. Photobiomodulation with near infrared light mitigates Alzheimer's disease-related pathology in cerebral cortex - evidence from two transgenic mouse models. Alzheimers Res Ther. 2014;6(1):2.

[a173] Purushothuman S, Johnstone DM, Nandasena C, van Eersel J, Ittner LM, Mitrofanis J, et al. Near infrared light mitigates cerebellar pathology in transgenic mouse models of dementia. Neurosci Lett. 2015;591:155-9.

[a174] Fukushima T, Nakamura A, Iwakami N, Nakada Y, Hattori H, Hoki S, et al. T-817MA, a neuroprotective agent, attenuates the motor and cognitive impairments associated with neuronal degeneration in P301L tau transgenic mice. Biochem Biophys Res Commun. 2011;407(4):730-4.

[a175] Shiryaev N, Jouroukhin Y, Giladi E, Polyzoidou E, Grigoriadis NC, Rosenmann H, et al. NAP protects memory, increases soluble tau and reduces tau hyperphosphorylation in a tauopathy model. Neurobiol Dis. 2009;34(2):381-8.

[a176] Rockenstein E, Ubhi K, Mante M, Florio J, Adame A, Winter S, et al. Neuroprotective effects of Cerebrolysin in triple repeat Tau transgenic model of Pick's disease and fronto-temporal tauopathies. BMC Neurosci. 2015;16(1):85.

[a177] Hull C, Dekeryte R, Buchanan H, Kamli-Salino S, Robertson A, Delibegovic M, et al. NLRP3 inflammasome inhibition with MCC950 improves insulin sensitivity and inflammation in a mouse model of frontotemporal dementia. Neuropharmacology. 2020;180:108305.

[a178] Warmus BA, Sekar DR, McCutchen E, Schellenberg GD, Roberts RC, McMahon LL, et al. Tau-mediated NMDA receptor impairment underlies dysfunction of a selectively vulnerable network in a mouse model of frontotemporal dementia. J Neurosci. 2014;34(49):16482-95.

[a179] Chang JK, Leso A, Subaiea GM, Lahouel A, Masoud A, Mushtaq F, et al. Tolfenamic Acid: A Modifier of the Tau Protein and its Role in Cognition and Tauopathy. Curr Alzheimer Res. 2018;15(7):655-63.

[a180] Wang Y, Guan PP, Yu X, Guo YS, Zhang YJ, Wang ZY, et al. COX-2 metabolic products, the prostaglandin I(2) and F(2alpha), mediate the effects of TNF-alpha and Zn(2+) in stimulating the phosphorylation of Tau. Oncotarget. 2017;8(59):99296-311.

[a181] Huang M, Tallon C, Zhu X, Huizar KDJ, Picciolini S, Thomas AG, et al. Microglial-Targeted nSMase2 Inhibitor Fails to Reduce Tau Propagation in PS19 Mice. Pharmaceutics. 2023;15(9).

[a182] Tallon C, Bell BJ, Malvankar MM, Deme P, Nogueras-Ortiz C, Eren E, et al. Inhibiting tau-induced elevated nSMase2 activity and ceramides is therapeutic in an Alzheimer's disease mouse model. Transl Neurodegener. 2023;12(1):56.

[a183] Vallés-Saiz L, Ávila J, Hernández F. Lamivudine (3TC), a Nucleoside Reverse Transcriptase Inhibitor, Prevents the Neuropathological Alterations Present in Mutant Tau Transgenic Mice. Int J Mol Sci. 2023;24(13).

[a184] Wang X, Li W, Marcus J, Pearson M, Song L, Smith K, et al. MK-8719, a Novel and Selective O-GlcNAcase Inhibitor That Reduces the Formation of Pathological Tau and Ameliorates Neurodegeneration in a Mouse Model of Tauopathy. J Pharmacol Exp Ther. 2020a;374(2):252-63.

[a185] Zhu Y, Shan X, Safarpour F, Erro Go N, Li N, Shan A, et al. Pharmacological Inhibition of O-GlcNAcase Enhances Autophagy in Brain through an mTOR-Independent Pathway. ACS Chem Neurosci. 2018;9(6):1366-79.

[a186] Bijttebier S, Rodrigues Martins D, Mertens L, Grauwen K, Bruinzeel W, Willems R, et al. IP-LC-MSMS Enables Identification of Three Tau O-GlcNAcylation Sites as O-GlcNAcase Inhibition Pharmacodynamic Readout in Transgenic Mice Overexpressing Human Tau. J Proteome Res. 2023;22(4):1309-21.

[a187] Borghgraef P, Menuet C, Theunis C, Louis JV, Devijver H, Maurin H, et al. Increasing brain protein O-GlcNAc-ylation mitigates breathing defects and mortality of Tau.P301L mice. PLoS One. 2013;8(12):e84442.

[a188] Graham DL, Gray AJ, Joyce JA, Yu D, O'Moore J, Carlson GA, et al. Increased O-GlcNAcylation reduces pathological tau without affecting its normal phosphorylation in a mouse model of tauopathy. Neuropharmacology. 2014;79:307-13.

[a189] Hastings NB, Wang X, Song L, Butts BD, Grotz D, Hargreaves R, et al. Inhibition of O-GlcNAcase leads to elevation of O-GlcNAc tau and reduction of tauopathy and cerebrospinal fluid tau in rTg4510 mice. Mol Neurodegener. 2017;12(1):39.

[a190] Rostgaard N, Jul PH, Garmer M, Volbracht C. Increasing O-GlcNAcylation Attenuates tau Hyperphosphorylation and Behavioral Impairment in rTg4510 Tauopathy Mice. J Integr Neurosci. 2023;22(5):135.

[a191] Wang X, Smith K, Pearson M, Hughes A, Cosden ML, Marcus J, et al. Early intervention of tau pathology prevents behavioral changes in the rTg4510 mouse model of tauopathy. PLoS One. 2018a;13(4):e0195486.

[a192] Yu Y, Zhang L, Li X, Run X, Liang Z, Li Y, et al. Differential effects of an O-GlcNAcase inhibitor on tau phosphorylation. PLoS One. 2012;7(4):e35277.

[a193] Yuzwa SA, Shan X, Macauley MS, Clark T, Skorobogatko Y, Vosseller K, et al. Increasing O-GlcNAc slows neurodegeneration and stabilizes tau against aggregation. Nat Chem Biol. 2012;8(4):393-9.

[a194] Ruan Z, Delpech JC, Venkatesan Kalavai S, Van Enoo AA, Hu J, Ikezu S, et al. P2RX7 inhibitor suppresses exosome secretion and disease phenotype in P301S tau transgenic mice. Mol Neurodegener. 2020;15(1):47.

[a195] Di Lauro C, Bianchi C, Sebastian-Serrano A, Soria-Tobar L, Alvarez-Castelao B, Nicke A, et al. P2X7 receptor blockade reduces tau induced toxicity, therapeutic implications in tauopathies. Prog Neurobiol. 2022;208:102173.

[a196] Bianchi C, Alvarez-Castelao B, Sebastián-Serrano Á, Di Lauro C, Soria-Tobar L, Nicke A, et al. P2X7 receptor inhibition ameliorates ubiquitin–proteasome system dysfunction associated with Alzheimer’s disease. Alzheimer's Research and Therapy. 2023;15(1).

[a197] Maphis N, Jiang S, Xu G, Kokiko-Cochran ON, Roy SM, Van Eldik LJ, et al. Selective suppression of the alpha isoform of p38 MAPK rescues late-stage tau pathology. Alzheimers Res Ther. 2016;8(1):54.

[a198] Yang T, Liu H, Tran KC, Leng A, Massa SM, Longo FM. Small-molecule modulation of the p75 neurotrophin receptor inhibits a wide range of tau molecular pathologies and their sequelae in P301S tauopathy mice. Acta Neuropathol Commun. 2020;8(1):156.

[a199] Schaler AW, Runyan AM, Clelland CL, Sydney EJ, Fowler SL, Figueroa HY, et al. PAC1 receptor-mediated clearance of tau in postsynaptic compartments attenuates tau pathology in mouse brain. Sci Transl Med. 2021;13(595).

[a200] Dumont M, Stack C, Elipenahli C, Jainuddin S, Gerges M, Starkova N, et al. Bezafibrate administration improves behavioral deficits and tau pathology in P301S mice. Hum Mol Genet. 2012;21(23):5091-105.

[a201] Schroeder SK, Joly-Amado A, Gordon MN, Morgan D. Tau-Directed Immunotherapy: A Promising Strategy for Treating Alzheimer's Disease and Other Tauopathies. J Neuroimmune Pharmacol. 2016;11(1):9-25.

[a202] d'Abramo C, Acker CM, Jimenez HT, Davies P. Tau passive immunotherapy in mutant P301L mice: antibody affinity versus specificity. PLoS One. 2013;8(4):e62402.

[a203] Ittner A, Bertz J, Suh LS, Stevens CH, Gotz J, Ittner LM. Tau-targeting passive immunization modulates aspects of pathology in tau transgenic mice. J Neurochem. 2015;132(1):135-45.

[a204] Lin Y, Rajamohamedsait HB, Sandusky-Beltran LA, Gamallo-Lana B, Mar A, Sigurdsson EM. Chronic PD-1 Checkpoint Blockade Does Not Affect Cognition or Promote Tau Clearance in a Tauopathy Mouse Model. Front Aging Neurosci. 2019;11:377.

[a205] Schaler AW, Myeku N. Cilostazol, a phosphodiesterase 3 inhibitor, activates proteasome-mediated proteolysis and attenuates tauopathy and cognitive decline. Transl Res. 2018;193:31-41.

[a206] Myeku N, Clelland CL, Emrani S, Kukushkin NV, Yu WH, Goldberg AL, et al. Tau-driven 26S proteasome impairment and cognitive dysfunction can be prevented early in disease by activating cAMP-PKA signaling. Nat Med. 2016;22(1):46-53.

[a207] Bruch J, Xu H, Rosler TW, De Andrade A, Kuhn PH, Lichtenthaler SF, et al. PERK activation mitigates tau pathology in vitro and in vivo. EMBO Mol Med. 2017;9(3):371-84.

[a208] Radford H, Moreno JA, Verity N, Halliday M, Mallucci GR. PERK inhibition prevents tau-mediated neurodegeneration in a mouse model of frontotemporal dementia. Acta Neuropathol. 2015;130(5):633-42.

[a209] Anglada-Huguet M, Rodrigues S, Hochgrafe K, Mandelkow E, Mandelkow EM. Inhibition of Tau aggregation with BSc3094 reduces Tau and decreases cognitive deficits in rTg4510 mice. Alzheimers Dement (N Y). 2021;7(1):e12170.

[a210] Zhang X, Zhang X, Zhong M, Zhao P, Guo C, Li Y, et al. Selection of a d-Enantiomeric Peptide Specifically Binding to PHF6 for Inhibiting Tau Aggregation in Transgenic Mice. ACS Chem Neurosci. 2020b;11(24):4240-53.

[a211] Hernandez CM, Barkey RE, Craven KM, Pedemonte KA, Alisantosa B, Sanchez JO, et al. Transfusion with Blood Plasma from Young Mice Affects rTg4510 Transgenic Tau Mice Modeling of Alzheimer's Disease. Brain Sci. 2023;13(6).

[a212] Hunt JB, Jr., Nash KR, Placides D, Moran P, Selenica ML, Abuqalbeen F, et al. Sustained Arginase 1 Expression Modulates Pathological Tau Deposits in a Mouse Model of Tauopathy. J Neurosci. 2015;35(44):14842-60.

[a213] Xu GB, Guan PP, Wang P. Prostaglandin A1 Decreases the Phosphorylation of Tau by Activating Protein Phosphatase 2A via a Michael Addition Mechanism at Cysteine 377. Mol Neurobiol. 2021;58(3):1114-27.

[a214] Beauchamp LC, Liu XM, Sedjahtera A, Bogeski M, Vella LJ, Bush AI, et al. S-Adenosylmethionine Rescues Cognitive Deficits in the rTg4510 Animal Model by Stabilizing Protein Phosphatase 2A and Reducing Phosphorylated Tau. J Alzheimers Dis. 2020;77(4):1705-15.

[a215] Ahmed T, Van der Jeugd A, Caillierez R, Buee L, Blum D, D'Hooge R, et al. Chronic Sodium Selenate Treatment Restores Deficits in Cognition and Synaptic Plasticity in a Murine Model of Tauopathy. Front Mol Neurosci. 2020;13:570223.

[a216] van Eersel J, Ke YD, Liu X, Delerue F, Kril JJ, Gotz J, et al. Sodium selenate mitigates tau pathology, neurodegeneration, and functional deficits in Alzheimer's disease models. Proc Natl Acad Sci U S A. 2010;107(31):13888-93.

[a217] McKenzie-Nickson S, Chan J, Perez K, Hung LW, Cheng L, Sedjahtera A, et al. Modulating Protein Phosphatase 2A Rescues Disease Phenotype in Neurodegenerative Tauopathies. ACS Chem Neurosci. 2018;9(11):2731-40.

[a218] Kunze LH, Ruch F, Biechele G, Eckenweber F, Wind-Mark K, Dinkel L, et al. Long-Term Pioglitazone Treatment Has No Significant Impact on Microglial Activation and Tau Pathology in P301S Mice. Int J Mol Sci. 2023;24(12).

[a219] Rosenzweig N, Dvir-Szternfeld R, Tsitsou-Kampeli A, Keren-Shaul H, Ben-Yehuda H, Weill-Raynal P, et al. PD-1/PD-L1 checkpoint blockade harnesses monocyte-derived macrophages to combat cognitive impairment in a tauopathy mouse model. Nat Commun. 2019;10(1):465.

[a220] Ben-Yehuda H, Arad M, Peralta Ramos JM, Sharon E, Castellani G, Ferrera S, et al. Key role of the CCR2-CCL2 axis in disease modification in a mouse model of tauopathy. Mol Neurodegener. 2021;16(1):39.

[a221] Etelainen TS, Silva MC, Uhari-Vaananen JK, De Lorenzo F, Jantti MH, Cui H, et al. A prolyl oligopeptidase inhibitor reduces tau pathology in cellular models and in mice with tauopathy. Sci Transl Med. 2023;15(691):eabq2915.

[a222] Damianich A, Facal CL, Muniz JA, Mininni C, Soiza-Reilly M, Ponce De Leon M, et al. Tau mis-splicing correlates with motor impairments and striatal dysfunction in a model of tauopathy. Brain. 2021;144(8):2302-9.

[a223] Espindola SL, Damianich A, Alvarez RJ, Sartor M, Belforte JE, Ferrario JE, et al. Modulation of Tau Isoforms Imbalance Precludes Tau Pathology and Cognitive Decline in a Mouse Model of Tauopathy. Cell Rep. 2018;23(3):709-15.

[a224] Shen LL, Manucat-Tan NB, Gao SH, Li WW, Zeng F, Zhu C, et al. The ProNGF/p75NTR pathway induces tau pathology and is a therapeutic target for FTLD-tau. Mol Psychiatry. 2018;23(8):1813-24.

[a225] Wang W, Zhou Q, Jiang T, Li S, Ye J, Zheng J, et al. A novel small-molecule PROTAC selectively promotes tau clearance to improve cognitive functions in Alzheimer-like models. Theranostics. 2021;11(11):5279-95.

[a226] Hou TY, Zhou Y, Zhu LS, Wang X, Pang P, Wang DQ, et al. Correcting abnormalities in miR-124/PTPN1 signaling rescues tau pathology in Alzheimer's disease. J Neurochem. 2020;154(4):441-57.

[a227] Zhong S, Ye J, Deng Y, Zhang M, Zou M, Yao X, et al. Quercetagitrin Inhibits Tau Accumulation and Reverses Neuroinflammation and Cognitive Deficits in P301S-Tau Transgenic Mice. Molecules. 2023;28(9).

[a228] Kim Y, Park H, Kim Y, Kim SH, Lee JH, Yang H, et al. Pathogenic Role of RAGE in Tau Transmission and Memory Deficits. Biol Psychiatry. 2023;93(9):829-41.

[a229] Collu R, Yin Z, Giunti E, Daley S, Chen M, Morin P, et al. Effect of the ROCK inhibitor fasudil on the brain proteomic profile in the tau transgenic mouse model of Alzheimer's disease. Front Aging Neurosci. 2024;16:1323563.

[a230] Hamano T, Shirafuji N, Yen SH, Yoshida H, Kanaan NM, Hayashi K, et al. Rho-kinase ROCK inhibitors reduce oligomeric tau protein. Neurobiol Aging. 2020;89:41-54.

[a231] Sun XY, Li LJ, Dong QX, Zhu J, Huang YR, Hou SJ, et al. Rutin prevents tau pathology and neuroinflammation in a mouse model of Alzheimer's disease. J Neuroinflammation. 2021;18(1):131.

[a232] Jimenez H, Adrien L, Wolin A, Eun J, Chang EH, Burstein ES, et al. The impact of pimavanserin on psychotic phenotypes and tau phosphorylation in the P301L/COMT- and rTg(P301L)4510 mouse models of Alzheimer's disease. Alzheimers Dement (N Y). 2022;8(1):e12247.

[a233] Spires-Jones TL, Fox LM, Rozkalne A, Pitstick R, Carlson GA, Kazantsev AG. Inhibition of Sirtuin 2 with Sulfobenzoic Acid Derivative AK1 is Non-Toxic and Potentially Neuroprotective in a Mouse Model of Frontotemporal Dementia. Front Pharmacol. 2012;3:42.

[a234] Vijayan M, George M, Bunquin LE, Bose C, Reddy PH. Protective effects of a small-molecule inhibitor DDQ against tau-induced toxicities in a transgenic tau mouse model of Alzheimer's disease. Hum Mol Genet. 2022;31(7):1022-34.

[a235] Williams JB, Cao Q, Wang W, Lee YH, Qin L, Zhong P, et al. Inhibition of histone methyltransferase Smyd3 rescues NMDAR and cognitive deficits in a tauopathy mouse model. Nat Commun. 2023;14(1):91.

[a236] Ren QG, Gong WG, Zhou H, Shu H, Wang YJ, Zhang ZJ. Spatial Training Ameliorates Long-Term Alzheimer's Disease-Like Pathological Deficits by Reducing NLRP3 Inflammasomes in PR5 Mice. Neurotherapeutics. 2019;16(2):450-64.

[a237] Tautou M, Eddarkaoui S, Descamps F, Larchanche PE, El Bakali J, Goveas LM, et al. A β-Secretase Modulator Decreases Tau Pathology and Preserves Short-Term Memory in a Mouse Model of Neurofibrillary Degeneration. Front Pharmacol. 2021;12:679335.

[a238] Wang YJ, Gong WG, Ren QG, Zhang ZJ. Escitalopram Alleviates Alzheimer's Disease-Type Tau Pathologies in the Aged P301L Tau Transgenic Mice. J Alzheimers Dis. 2020b;77(2):807-19.

[a239] de Oliveira P, Cella C, Locker N, Ravindran KKG, Mendis A, Wafford K, et al. Improved Sleep, Memory, and Cellular Pathological Features of Tauopathy, Including the NLRP3 Inflammasome, after Chronic Administration of Trazodone in rTg4510 Mice. J Neurosci. 2022;42(16):3494-509.

[a240] Litvinchuk A, Wan YW, Swartzlander DB, Chen F, Cole A, Propson NE, et al. Complement C3aR Inactivation Attenuates Tau Pathology and Reverses an Immune Network Deregulated in Tauopathy Models and Alzheimer's Disease. Neuron. 2018;100(6):1337-53 e5.

[a241] Paris D, Ait-Ghezala G, Bachmeier C, Laco G, Beaulieu-Abdelahad D, Lin Y, et al. The spleen tyrosine kinase (Syk) regulates Alzheimer amyloid-beta production and Tau hyperphosphorylation. J Biol Chem. 2014;289(49):33927-44.

[a242] Schweig JE, Yao H, Coppola K, Jin C, Crawford F, Mullan M, et al. Spleen tyrosine kinase (SYK) blocks autophagic Tau degradation in vitro and in vivo. J Biol Chem. 2019;294(36):13378-95.

[a243] Laurent C, Dorothee G, Hunot S, Martin E, Monnet Y, Duchamp M, et al. Hippocampal T cell infiltration promotes neuroinflammation and cognitive decline in a mouse model of tauopathy. Brain. 2017;140(1):184-200.

[a244] Maphis NM, Peabody J, Crossey E, Jiang S, Jamaleddin Ahmad FA, Alvarez M, et al. Qβ Virus-like particle-based vaccine induces robust immunity and protects against tauopathy. NPJ Vaccines. 2019;4(1):26.

[a245] Zhang D, Zhang W, Ming C, Gao X, Yuan H, Lin X, et al. P-tau217 correlates with neurodegeneration in Alzheimer's disease, and targeting p-tau217 with immunotherapy ameliorates murine tauopathy. Neuron. 2024;112(10):1676-93.e12.

[a246] Bright J, Hussain S, Dang V, Wright S, Cooper B, Byun T, et al. Human secreted tau increases amyloid-beta production. Neurobiol Aging. 2015;36(2):693-709.

[a247] Sopko R, Golonzhka O, Arndt J, Quan C, Czerkowicz J, Cameron A, et al. Characterization of tau binding by gosuranemab. Neurobiol Dis. 2020;146:105120.

[a248] Boimel M, Grigoriadis N, Lourbopoulos A, Haber E, Abramsky O, Rosenmann H. Efficacy and safety of immunization with phosphorylated tau against neurofibrillary tangles in mice. Exp Neurol. 2010;224(2):472-85.

[a249] Richter M, Mewes A, Fritsch M, Krugel U, Hoffmann R, Singer D. Doubly Phosphorylated Peptide Vaccines to Protect Transgenic P301S Mice against Alzheimer's Disease Like Tau Aggregation. Vaccines (Basel). 2014;2(3):601-23.

[a250] Agadjanyan MG, Zagorski K, Petrushina I, Davtyan H, Kazarian K, Antonenko M, et al. Humanized monoclonal antibody armanezumab specific to N-terminus of pathological tau: characterization and therapeutic potency. Mol Neurodegener. 2017;12(1):33.

[a251] Davtyan H, Chen WW, Zagorski K, Davis J, Petrushina I, Kazarian K, et al. MultiTEP platform-based DNA epitope vaccine targeting N-terminus of tau induces strong immune responses and reduces tau pathology in THY-Tau22 mice. Vaccine. 2017;35(16):2015-24.

[a252] Goodwin MS, Sinyavskaya O, Burg F, O'Neal V, Ceballos-Diaz C, Cruz PE, et al. Anti-tau scFvs Targeted to the Cytoplasm or Secretory Pathway Variably Modify Pathology and Neurodegenerative Phenotypes. Mol Ther. 2021;29(2):859-72.

[a253] Ji M, Xie XX, Liu DQ, Yu XL, Zhang Y, Zhang LX, et al. Hepatitis B core VLP-based mis-disordered tau vaccine elicits strong immune response and alleviates cognitive deficits and neuropathology progression in Tau.P301S mouse model of Alzheimer's disease and frontotemporal dementia. Alzheimers Res Ther. 2018;10(1):55.

[a254] Nisbet RM, Van der Jeugd A, Leinenga G, Evans HT, Janowicz PW, Gotz J. Combined effects of scanning ultrasound and a tau-specific single chain antibody in a tau transgenic mouse model. Brain. 2017;140(5):1220-30.

[a255] Asuni AA, Boutajangout A, Quartermain D, Sigurdsson EM. Immunotherapy targeting pathological tau conformers in a tangle mouse model reduces brain pathology with associated functional improvements. J Neurosci. 2007;27(34):9115-29.

[a256] Theunis C, Crespo-Biel N, Gafner V, Pihlgren M, Lopez-Deber MP, Reis P, et al. Efficacy and safety of a liposome-based vaccine against protein Tau, assessed in tau.P301L mice that model tauopathy. PLoS One. 2013;8(8):e72301.

[a257] Bi M, Ittner A, Ke YD, Gotz J, Ittner LM. Tau-targeted immunization impedes progression of neurofibrillary histopathology in aged P301L tau transgenic mice. PLoS One. 2011;6(12):e26860.

[a258] Bajracharya R, Brici D, Bodea LG, Janowicz PW, Gotz J, Nisbet RM. Tau antibody isotype induces differential effects following passive immunisation of tau transgenic mice. Acta Neuropathol Commun. 2021;9(1):42.

[a259] Song HL, Kim NY, Park J, Kim MI, Jeon YN, Lee SJ, et al. Monoclonal antibody Y01 prevents tauopathy progression induced by lysine 280-acetylated tau in cell and mouse models. J Clin Invest. 2023;133(8).

[a260] Okuda M, Hijikuro I, Fujita Y, Wu X, Nakayama S, Sakata Y, et al. PE859, a novel tau aggregation inhibitor, reduces aggregated tau and prevents onset and progression of neural dysfunction in vivo. PLoS One. 2015;10(2):e0117511.

[a261] Foster K, Manca M, McClure K, Koivula P, Trojanowski JQ, Havas D, et al. Preclinical characterization and IND-enabling safety studies for PNT001, an antibody that recognizes cis-pT231 tau. Alzheimers Dement. 2023.

[a262] Vemula P, Schoch KM, Miller TM. Evaluating the efficacy of purchased antisense oligonucleotides to reduce mouse and human tau in vivo. Front Mol Neurosci. 2023;16:1320182.

[a263] Easton A, Jensen ML, Wang C, Hagedorn PH, Li Y, Weed M, et al. Identification and characterization of a MAPT-targeting locked nucleic acid antisense oligonucleotide therapeutic for tauopathies. Mol Ther Nucleic Acids. 2022;29:625-42.

[a264] Xu H, Rosler TW, Carlsson T, de Andrade A, Fiala O, Hollerhage M, et al. Tau silencing by siRNA in the P301S mouse model of tauopathy. Curr Gene Ther. 2014;14(5):343-51.

[a265] DeVos SL, Miller RL, Schoch KM, Holmes BB, Kebodeaux CS, Wegener AJ, et al. Tau reduction prevents neuronal loss and reverses pathological tau deposition and seeding in mice with tauopathy. Sci Transl Med. 2017;9(374).

[a266] Facal CL, Fernández Bessone I, Muñiz JA, Pereyra AE, Pedroncini O, Páez-Paz I, et al. Tau reduction with artificial microRNAs modulates neuronal physiology and improves tauopathy phenotypes in mice. Mol Ther. 2024;32(4):1080-95.

[a267] Bajracharya R, Cruz E, Gotz J, Nisbet RM. Ultrasound-mediated delivery of novel tau-specific monoclonal antibody enhances brain uptake but not therapeutic efficacy. J Control Release. 2022;349:634-48.

[a268] Chai X, Wu S, Murray TK, Kinley R, Cella CV, Sims H, et al. Passive immunization with anti-Tau antibodies in two transgenic models: reduction of Tau pathology and delay of disease progression. J Biol Chem. 2011;286(39):34457-67.

[a269] Vitale F, Ortolan J, Volpe BT, Marambaud P, Giliberto L, d'Abramo C. Intramuscular injection of vectorized-scFvMC1 reduces pathological tau in two different tau transgenic models. Acta Neuropathol Commun. 2020;8(1):126.

[a270] Vitale F, Giliberto L, Ruiz S, Steslow K, Marambaud P, d'Abramo C. Anti-tau conformational scFv MC1 antibody efficiently reduces pathological tau species in adult JNPL3 mice. Acta Neuropathol Commun. 2018;6(1):82.

[a271] Hovakimyan A, Antonyan T, Shabestari SK, Svystun O, Chailyan G, Coburn MA, et al. A MultiTEP platform-based epitope vaccine targeting the phosphatase activating domain (PAD) of tau: therapeutic efficacy in PS19 mice. Sci Rep. 2019;9(1):15455.

[a272] Joly-Amado A, Davtyan H, Serraneau K, Jules P, Zitnyar A, Pressman E, et al. Active immunization with tau epitope in a mouse model of tauopathy induced strong antibody response together with improvement in short memory and pSer396-tau pathology. Neurobiol Dis. 2020;134:104636.

[a273] Castillo-Carranza DL, Sengupta U, Guerrero-Munoz MJ, Lasagna-Reeves CA, Gerson JE, Singh G, et al. Passive immunization with Tau oligomer monoclonal antibody reverses tauopathy phenotypes without affecting hyperphosphorylated neurofibrillary tangles. J Neurosci. 2014;34(12):4260-72.

[a274] Hussong SA, Banh AQ, Van Skike CE, Dorigatti AO, Hernandez SF, Hart MJ, et al. Soluble pathogenic tau enters brain vascular endothelial cells and drives cellular senescence and brain microvascular dysfunction in a mouse model of tauopathy. Nat Commun. 2023;14(1):2367.

[a275] Schroeder S, Joly-Amado A, Soliman A, Sengupta U, Kayed R, Gordon MN, et al. Oligomeric tau-targeted immunotherapy in Tg4510 mice. Alzheimers Res Ther. 2017;9(1):46.

[a276] Bittar A, Al-Lahham R, Bhatt N, Moore K, Montalbano M, Jerez C, et al. Passive Immunotherapy Targeting Tau Oligomeric Strains Reverses Tauopathy Phenotypes in Aged Human-Tau Mice in a Mouse Model-Specific Manner. J Alzheimers Dis. 2022;90(3):1103-22.

[a277] Selenica ML, Davtyan H, Housley SB, Blair LJ, Gillies A, Nordhues BA, et al. Epitope analysis following active immunization with tau proteins reveals immunogens implicated in tau pathogenesis. J Neuroinflammation. 2014b;11(1):152.

[a278] d'Abramo C, Acker CM, Jimenez H, Davies P. Passive Immunization in JNPL3 Transgenic Mice Using an Array of Phospho-Tau Specific Antibodies. PLoS One. 2015;10(8):e0135774.

[a279] Sankaranarayanan S, Barten DM, Vana L, Devidze N, Yang L, Cadelina G, et al. Passive immunization with phospho-tau antibodies reduces tau pathology and functional deficits in two distinct mouse tauopathy models. PLoS One. 2015;10(5):e0125614.

[a280] Umeda T, Eguchi H, Kunori Y, Matsumoto Y, Taniguchi T, Mori H, et al. Passive immunotherapy of tauopathy targeting pSer413-tau: a pilot study in mice. Ann Clin Transl Neurol. 2015;2(3):241-55.

[a281] Wu Q, Bai Y, Li W, Congdon EE, Liu W, Lin Y, et al. Increased neuronal activity in motor cortex reveals prominent calcium dyshomeostasis in tauopathy mice. Neurobiol Dis. 2021;147:105165.

[a282] Boutajangout A, Ingadottir J, Davies P, Sigurdsson EM. Passive immunization targeting pathological phospho-tau protein in a mouse model reduces functional decline and clears tau aggregates from the brain. J Neurochem. 2011;118(4):658-67.

[a283] Congdon EE, Lin Y, Rajamohamedsait HB, Shamir DB, Krishnaswamy S, Rajamohamedsait WJ, et al. Affinity of Tau antibodies for solubilized pathological Tau species but not their immunogen or insoluble Tau aggregates predicts in vivo and ex vivo efficacy. Mol Neurodegener. 2016;11(1):62.

[a284] Liu W, Zhao L, Blackman B, Parmar M, Wong MY, Woo T, et al. Vectored Intracerebral Immunization with the Anti-Tau Monoclonal Antibody PHF1 Markedly Reduces Tau Pathology in Mutant Tau Transgenic Mice. J Neurosci. 2016;36(49):12425-35.

[a285] Lee SH, Le Pichon CE, Adolfsson O, Gafner V, Pihlgren M, Lin H, et al. Antibody-Mediated Targeting of Tau In Vivo Does Not Require Effector Function and Microglial Engagement. Cell Rep. 2016;16(6):1690-700.

[a286] Troquier L, Caillierez R, Burnouf S, Fernandez-Gomez FJ, Grosjean ME, Zommer N, et al. Targeting phospho-Ser422 by active Tau Immunotherapy in the THYTau22 mouse model: a suitable therapeutic approach. Curr Alzheimer Res. 2012;9(4):397-405.

[a287] Davidowitz EJ, Krishnamurthy PK, Lopez P, Jimenez H, Adrien L, Davies P, et al. In Vivo Validation of a Small Molecule Inhibitor of Tau Self-Association in htau Mice. J Alzheimers Dis. 2020;73(1):147-61.

[a288] Davidowitz EJ, Lopez P, Jimenez H, Adrien L, Davies P, Moe JG. Small molecule inhibitor of tau self-association in a mouse model of tauopathy: A preventive study in P301L tau JNPL3 mice. PLoS One. 2023;18(8):e0286523.

[a289] Yukawa K, Yamamoto-Mcguire S, Cafaro L, Hong C, Kamme F, Ikezu T, et al. Antisense oligonucleotide-based targeting of Tau-tubulin kinase 1 prevents hippocampal accumulation of phosphorylated tau in PS19 tauopathy mice. Acta Neuropathol Commun. 2023;11(1):166.

[a290] Spencer B, Bruschweiler S, Sealey-Cardona M, Rockenstein E, Adame A, Florio J, et al. Selective targeting of 3 repeat Tau with brain penetrating single chain antibodies for the treatment of neurodegenerative disorders. Acta Neuropathol. 2018;136(1):69-87.

[a291] Garcia-Arriaza J, Marin MQ, Merchan-Rubira J, Mascaraque SM, Medina M, Avila J, et al. Tauopathy Analysis in P301S Mouse Model of Alzheimer Disease Immunized With DNA and MVA Poxvirus-Based Vaccines Expressing Human Full-Length 4R2N or 3RC Tau Proteins. Vaccines (Basel). 2020;8(1).

[a292] Martini-Stoica H, Cole AL, Swartzlander DB, Chen F, Wan YW, Bajaj L, et al. TFEB enhances astroglial uptake of extracellular tau species and reduces tau spreading. J Exp Med. 2018;215(9):2355-77.

[a293] Polito VA, Li H, Martini-Stoica H, Wang B, Yang L, Xu Y, et al. Selective clearance of aberrant tau proteins and rescue of neurotoxicity by transcription factor EB. EMBO Mol Med. 2014;6(9):1142-60.

[a294] Yang C, Su C, Iyaswamy A, Krishnamoorthi SK, Zhu Z, Yang S, et al. Celastrol enhances transcription factor EB (TFEB)-mediated autophagy and mitigates Tau pathology: Implications for Alzheimer's disease therapy. Acta Pharm Sin B. 2022a;12(4):1707-22.

[a295] Song JX, Malampati S, Zeng Y, Durairajan SSK, Yang CB, Tong BC, et al. A small molecule transcription factor EB activator ameliorates beta-amyloid precursor protein and Tau pathology in Alzheimer's disease models. Aging Cell. 2020;19(2):e13069.

[a296] Iannucci J, Johnson SL, Majchrzak M, Barlock BJ, Akhlaghi F, Seeram NP, et al. Short-term treatment with dabigatran alters protein expression patterns in a late-stage tau-based Alzheimer's disease mouse model. Biochem Biophys Rep. 2020;24:100862.

[a297] Dutta D, Jana M, Paidi RK, Majumder M, Raha S, Dasarathy S, et al. Tau fibrils induce glial inflammation and neuropathology via TLR2 in Alzheimer’s disease-related mouse models. Journal of Clinical Investigation. 2023;133(18).

[a298] Qin Y, Liu Y, Hao W, Decker Y, Tomic I, Menger MD, et al. Stimulation of TLR4 Attenuates Alzheimer's Disease-Related Symptoms and Pathology in Tau-Transgenic Mice. J Immunol. 2016;197(8):3281-92.

[a299] Sebastian-Serrano A, Merchan-Rubira J, Di Lauro C, Bianchi C, Soria-Tobar L, Narisawa S, et al. TNAP upregulation is a critical factor in Tauopathies and its blockade ameliorates neurotoxicity and increases life-expectancy. Neurobiol Dis. 2022;165:105632.

[a300] Ou W, Yang J, Simanauskaite J, Choi M, Castellanos DM, Chang R, et al. Biologic TNF-alpha inhibitors reduce microgliosis, neuronal loss, and tau phosphorylation in a transgenic mouse model of tauopathy. J Neuroinflammation. 2021;18(1):312.

[a301] Tong BC, Huang AS, Wu AJ, Iyaswamy A, Ho OK, Kong AH, et al. Tetrandrine ameliorates cognitive deficits and mitigates tau aggregation in cell and animal models of tauopathies. J Biomed Sci. 2022;29(1):85.

[a302] Zhang X, Tang L, Yang J, Meng L, Chen J, Zhou L, et al. Soluble TREM2 ameliorates tau phosphorylation and cognitive deficits through activating transgelin-2 in Alzheimer's disease. Nat Commun. 2023;14(1):6670.

[a303] Fassler M, Benaim C, George J. TREM2 Agonism with a Monoclonal Antibody Attenuates Tau Pathology and Neurodegeneration. Cells. 2023;12(11).

[a304] Jiang T, Zhang YD, Chen Q, Gao Q, Zhu XC, Zhou JS, et al. TREM2 modifies microglial phenotype and provides neuroprotection in P301S tau transgenic mice. Neuropharmacology. 2016;105:196-206.

[a305] Zhong L, Sheng X, Wang W, Li Y, Zhuo R, Wang K, et al. TREM2 receptor protects against complement-mediated synaptic loss by binding to complement C1q during neurodegeneration. Immunity. 2023;56(8):1794-808.e8.

[a306] Zhang B, Yao Y, Cornec AS, Oukoloff K, James MJ, Koivula P, et al. A brain-penetrant triazolopyrimidine enhances microtubule-stability, reduces axonal dysfunction and decreases tau pathology in a mouse tauopathy model. Mol Neurodegener. 2018a;13(1):59.

[a307] Javidnia M, Hebron ML, Xin Y, Kinney NG, Moussa CE. Pazopanib Reduces Phosphorylated Tau Levels and Alters Astrocytes in a Mouse Model of Tauopathy. J Alzheimers Dis. 2017;60(2):461-81.

[a308] Karakatsani ME, Kugelman T, Ji R, Murillo M, Wang S, Niimi Y, et al. Unilateral Focused Ultrasound-Induced Blood-Brain Barrier Opening Reduces Phosphorylated Tau from The rTg4510 Mouse Model. Theranostics. 2019;9(18):5396-411.

[a309] Géraudie A, Riche M, Lestra T, Trotier A, Dupuis L, Mathon B, et al. Effects of Low-Intensity Pulsed Ultrasound-Induced Blood-Brain Barrier Opening in P301S Mice Modeling Alzheimer's Disease Tauopathies. Int J Mol Sci. 2023;24(15).

[a310] Pandit R, Leinenga G, Gotz J. Repeated ultrasound treatment of tau transgenic mice clears neuronal tau by autophagy and improves behavioral functions. Theranostics. 2019;9(13):3754-67.

[a311] Suk HJ, Buie N, Xu G, Banerjee A, Boyden ES, Tsai LH. Vibrotactile stimulation at gamma frequency mitigates pathology related to neurodegeneration and improves motor function. Front Aging Neurosci. 2023;15:1129510.

[a312] Tapias V, Jainuddin S, Ahuja M, Stack C, Elipenahli C, Vignisse J, et al. Benfotiamine treatment activates the Nrf2/ARE pathway and is neuroprotective in a transgenic mouse model of tauopathy. Hum Mol Genet. 2018;27(16):2874-92.

[a313] Ano Y, Ohya R, Takaichi Y, Washinuma T, Uchida K, Takashima A, et al. beta-Lactolin, a Whey-Derived Lacto-Tetrapeptide, Prevents Alzheimer's Disease Pathologies and Cognitive Decline. J Alzheimers Dis. 2020;73(4):1331-42.

[a314] Iyaswamy A, Krishnamoorthi SK, Liu YW, Song JX, Kammala AK, Sreenivasmurthy SG, et al. Yuan-Hu Zhi Tong Prescription Mitigates Tau Pathology and Alleviates Memory Deficiency in the Preclinical Models of Alzheimer's Disease. Front Pharmacol. 2020;11:584770.
