# Supplementary material for: Twenty years of therapeutic development in tauopathy mouse models: a scoping review
Source: Alzheimers Dement. 2025 Aug 18;21(8):e70578. doi: 10.1002/alz.70578 (PMC12360913; doi:10.1002/alz.70578)
Supplement: Supplementary file 2 — Supporting Information [file ALZ-21-e70578-s005.docx]

**Supplemental References 2:** References for Table S3.

[b1] Yoshiyama Y, Higuchi M, Zhang B, Huang SM, Iwata N, Saido TC, et al. Synapse loss and microglial activation precede tangles in a P301S tauopathy mouse model. Neuron. 2007;53(3):337-51.

[b2] Santacruz K, Lewis J, Spires T, Paulson J, Kotilinek L, Ingelsson M, et al. Tau suppression in a neurodegenerative mouse model improves memory function. Science. 2005;309(5733):476-81.

[b3] Ramsden M, Kotilinek L, Forster C, Paulson J, McGowan E, SantaCruz K, et al. Age-dependent neurofibrillary tangle formation, neuron loss, and memory impairment in a mouse model of human tauopathy (P301L). J Neurosci. 2005;25(46):10637-47.

[b4] Gamache J, Benzow K, Forster C, Kemper L, Hlynialuk C, Furrow E, et al. Factors other than hTau overexpression that contribute to tauopathy-like phenotype in rTg4510 mice. Nat Commun. 2019;10(1):2479.

[b5] Lewis J, McGowan E, Rockwood J, Melrose H, Nacharaju P, Van Slegtenhorst M, et al. Neurofibrillary tangles, amyotrophy and progressive motor disturbance in mice expressing mutant (P301L) tau protein. Nat Genet. 2000;25(4):402-5.

[b6] Allen B, Ingram E, Takao M, Smith MJ, Jakes R, Virdee K, et al. Abundant tau filaments and nonapoptotic neurodegeneration in transgenic mice expressing human P301S tau protein. J Neurosci. 2002;22(21):9340-51.

[b7] Andorfer C, Kress Y, Espinoza M, de Silva R, Tucker KL, Barde YA, et al. Hyperphosphorylation and aggregation of tau in mice expressing normal human tau isoforms. J Neurochem. 2003;86(3):582-90.

[b8] Gotz J, Chen F, Barmettler R, Nitsch RM. Tau filament formation in transgenic mice expressing P301L tau. J Biol Chem. 2001;276(1):529-34.

[b9] Pennanen L, Wolfer DP, Nitsch RM, Gotz J. Impaired spatial reference memory and increased exploratory behavior in P301L tau transgenic mice. Genes Brain Behav. 2006;5(5):369-79.

[b10] Schindowski K, Bretteville A, Leroy K, Begard S, Brion JP, Hamdane M, et al. Alzheimer's disease-like tau neuropathology leads to memory deficits and loss of functional synapses in a novel mutated tau transgenic mouse without any motor deficits. Am J Pathol. 2006;169(2):599-616.

[b11] Bondulich MK, Guo T, Meehan C, Manion J, Rodriguez Martin T, Mitchell JC, et al. Tauopathy induced by low level expression of a human brain-derived tau fragment in mice is rescued by phenylbutyrate. Brain. 2016;139(Pt 8):2290-306.

[b12] Umeda T, Yamashita T, Kimura T, Ohnishi K, Takuma H, Ozeki T, et al. Neurodegenerative disorder FTDP-17-related tau intron 10 +16C --> T mutation increases tau exon 10 splicing and causes tauopathy in transgenic mice. Am J Pathol. 2013;183(1):211-25.

[b13] Rosenmann H, Grigoriadis N, Eldar-Levy H, Avital A, Rozenstein L, Touloumi O, et al. A novel transgenic mouse expressing double mutant tau driven by its natural promoter exhibits tauopathy characteristics. Exp Neurol. 2008;212(1):71-84.

[b14] Ittner LM, Fath T, Ke YD, Bi M, van Eersel J, Li KM, et al. Parkinsonism and impaired axonal transport in a mouse model of frontotemporal dementia. Proc Natl Acad Sci U S A. 2008;105(41):15997-6002.

[b15] Rockenstein E, Overk CR, Ubhi K, Mante M, Patrick C, Adame A, et al. A novel triple repeat mutant tau transgenic model that mimics aspects of pick's disease and fronto-temporal tauopathies. PLoS One. 2015;10(3):e0121570.

[b16] Terwel D, Lasrado R, Snauwaert J, Vandeweert E, Van Haesendonck C, Borghgraef P, et al. Changed conformation of mutant Tau-P301L underlies the moribund tauopathy, absent in progressive, nonlethal axonopathy of Tau-4R/2N transgenic mice. J Biol Chem. 2005;280(5):3963-73.

[b17] Kim Y, Choi H, Lee W, Park H, Kam TI, Hong SH, et al. Caspase-cleaved tau exhibits rapid memory impairment associated with tau oligomers in a transgenic mouse model. Neurobiol Dis. 2016;87:19-28.

[b18] Tanemura K, Akagi T, Murayama M, Kikuchi N, Murayama O, Hashikawa T, et al. Formation of filamentous tau aggregations in transgenic mice expressing V337M human tau. Neurobiol Dis. 2001;8(6):1036-45.

[b19] Flunkert S, Hierzer M, Loffler T, Rabl R, Neddens J, Duller S, et al. Elevated levels of soluble total and hyperphosphorylated tau result in early behavioral deficits and distinct changes in brain pathology in a new tau transgenic mouse model. Neurodegener Dis. 2013;11(4):194-205.

[b20] Eckermann K, Mocanu MM, Khlistunova I, Biernat J, Nissen A, Hofmann A, et al. The beta-propensity of Tau determines aggregation and synaptic loss in inducible mouse models of tauopathy. J Biol Chem. 2007;282(43):31755-65.

[b21] Mocanu MM, Nissen A, Eckermann K, Khlistunova I, Biernat J, Drexler D, et al. The potential for beta-structure in the repeat domain of tau protein determines aggregation, synaptic decay, neuronal loss, and coassembly with endogenous Tau in inducible mouse models of tauopathy. J Neurosci. 2008;28(3):737-48.

[b22] Koss DJ, Robinson L, Drever BD, Plucinska K, Stoppelkamp S, Veselcic P, et al. Mutant Tau knock-in mice display frontotemporal dementia relevant behaviour and histopathology. Neurobiol Dis. 2016;91:105-23.

[b23] van Eersel J, Stevens CH, Przybyla M, Gladbach A, Stefanoska K, Chan CK, et al. Early-onset axonal pathology in a novel P301S-Tau transgenic mouse model of frontotemporal lobar degeneration. Neuropathol Appl Neurobiol. 2015;41(7):906-25.

[b24] Ishihara T, Hong M, Zhang B, Nakagawa Y, Lee MK, Trojanowski JQ, et al. Age-dependent emergence and progression of a tauopathy in transgenic mice overexpressing the shortest human tau isoform. Neuron. 1999;24(3):751-62.

[b25] Melis V, Zabke C, Stamer K, Magbagbeolu M, Schwab K, Marschall P, et al. Different pathways of molecular pathophysiology underlie cognitive and motor tauopathy phenotypes in transgenic models for Alzheimer's disease and frontotemporal lobar degeneration. Cell Mol Life Sci. 2015;72(11):2199-222.

[b26] McMillan P, Korvatska E, Poorkaj P, Evstafjeva Z, Robinson L, Greenup L, et al. Tau isoform regulation is region- and cell-specific in mouse brain. J Comp Neurol. 2008;511(6):788-803.

[b27] Warmus BA, Sekar DR, McCutchen E, Schellenberg GD, Roberts RC, McMahon LL, et al. Tau-mediated NMDA receptor impairment underlies dysfunction of a selectively vulnerable network in a mouse model of frontotemporal dementia. J Neurosci. 2014;34(49):16482-95.

[b28] Javidnia M, Hebron ML, Xin Y, Kinney NG, Moussa CE. Pazopanib Reduces Phosphorylated Tau Levels and Alters Astrocytes in a Mouse Model of Tauopathy. J Alzheimers Dis. 2017;60(2):461-81.

[b29] Hebron ML, Javidnia M, Moussa CE. Tau clearance improves astrocytic function and brain glutamate-glutamine cycle. J Neurol Sci. 2018;391:90-9.

[b30] Shim SB, Lim HJ, Chae KR, Kim CK, Hwang DY, Jee SW, et al. Tau overexpression in transgenic mice induces glycogen synthase kinase 3beta and beta-catenin phosphorylation. Neuroscience. 2007;146(2):730-40.

[b31] Boekhoorn K, Terwel D, Biemans B, Borghgraef P, Wiegert O, Ramakers GJ, de Vos K, Krugers H, Tomiyama T, Mori H, Joels M, van Leuven F, Lucassen PJ. Improved long-term potentiation and memory in young tau-P301L transgenic mice before onset of hyperphosphorylation and tauopathy. J Neurosci. 2006;26(13):3514-23.
