# Supplementary material for: Twenty years of therapeutic development in tauopathy mouse models: a scoping review
Source: Alzheimers Dement. 2025 Aug 18;21(8):e70578. doi: 10.1002/alz.70578 (PMC12360913; doi:10.1002/alz.70578)
Supplement: Supplementary file 4 — Supporting Information [file ALZ-21-e70578-s002.pdf]

Figure S1: Summary of pathological tau endpoint evaluations and their therapeutic outcomes grouped by treatment strategy

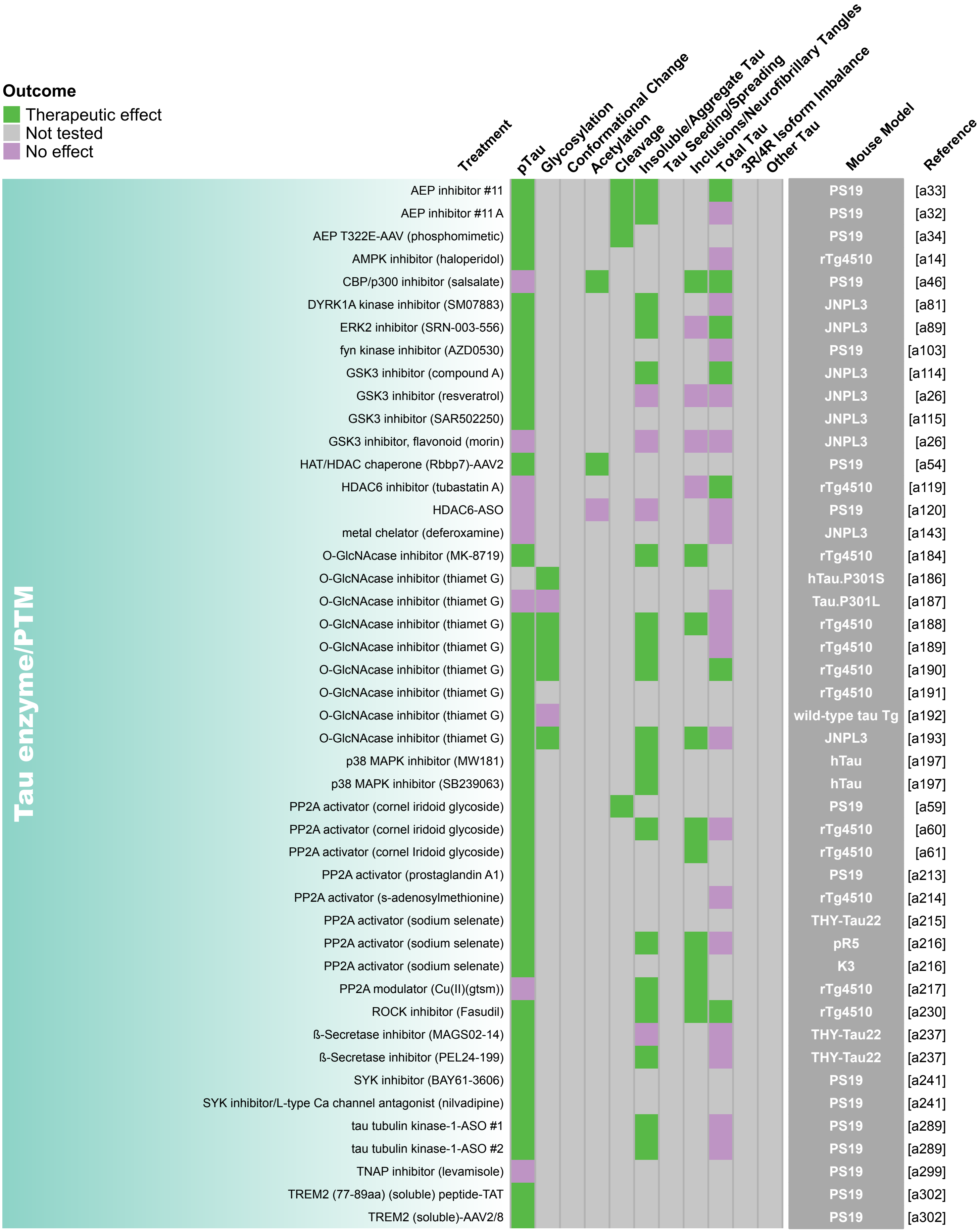

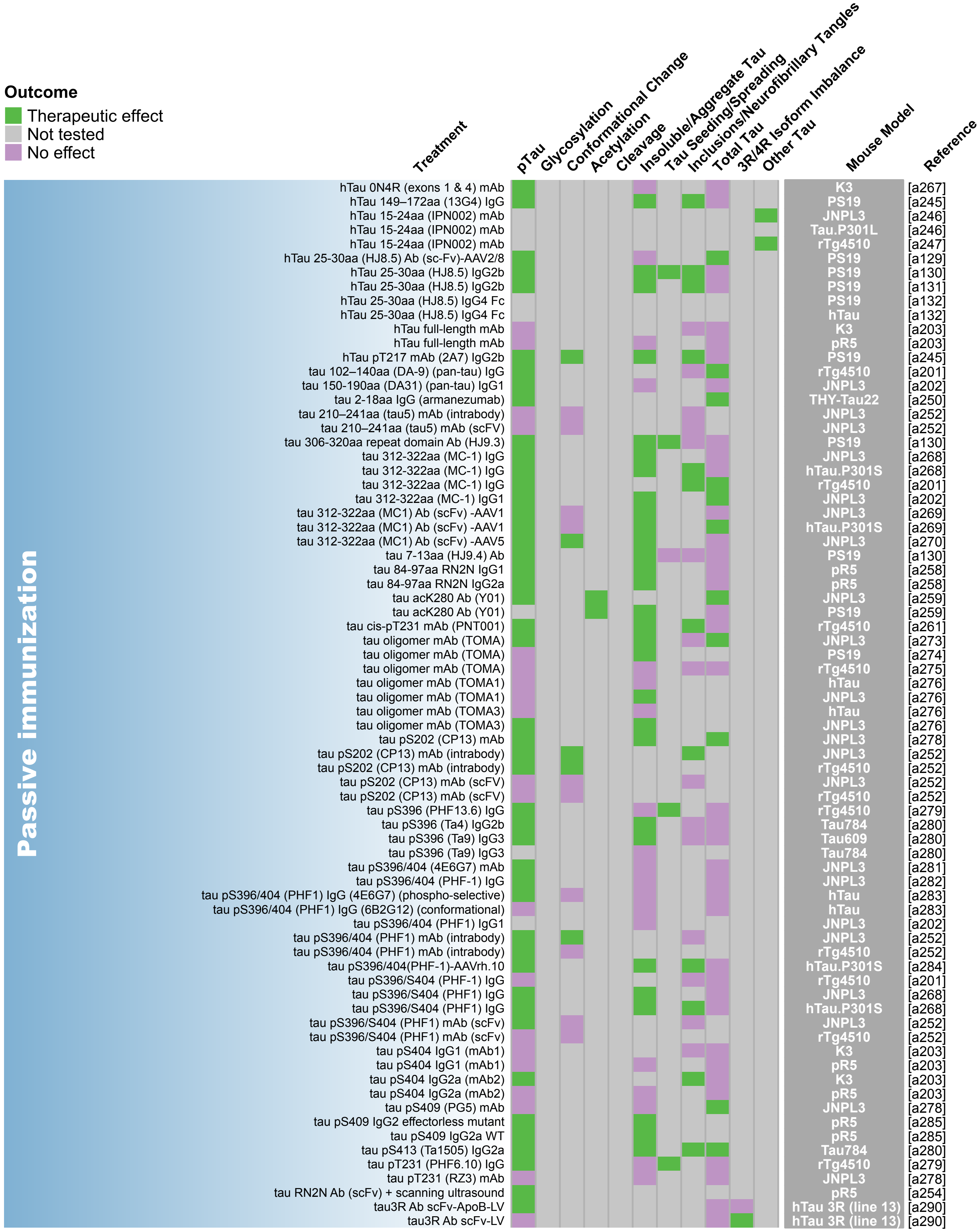
Outcome
Therapeutic effect
Not tested
No effect
Treatment
pTau
Glycosylation
Conformational Change
Acetylation
Cleavage
Insoluble/Aggregate Tau
Tau Seeding/Spreading
Inclusions/Neurofibrillary Tangles
Total Tau
3R/4R Isoform Imbalance
Other Tau
Mouse Model
Reference
Passive immunization
hTau 0N4R (exons 1 & 4) mAb K3 [a267]
hTau 149–172aa (13G4) IgG PS19 [a245]
hTau 15-24aa (IPN002) mAb JNPL3 [a246]
hTau 15-24aa (IPN002) mAb Tau.P301L [a246]
hTau 15-24aa (IPN002) mAb rTg4510 [a247]
hTau 25-30aa (HJ8.5) Ab (sc-Fv)-AAV2/8 PS19 [a129]
hTau 25-30aa (HJ8.5) IgG2b PS19 [a130]
hTau 25-30aa (HJ8.5) IgG2b PS19 [a131]
hTau 25-30aa (HJ8.5) IgG4 Fc PS19 [a132]
hTau 25-30aa (HJ8.5) IgG4 Fc hTau [a132]
hTau full-length mAb K3 [a203]
hTau full-length mAb pR5 [a203]
hTau pT217 mAb (2A7) IgG2b PS19 [a245]
tau 102–140aa (DA-9) (pan-tau) IgG rTg4510 [a201]
tau 150-190aa (DA31) (pan-tau) IgG1 JNPL3 [a202]
tau 2-18aa IgG (armanezumab) THY-Tau22 [a250]
tau 210–241aa (tau5) mAb (intrabody) JNPL3 [a252]
tau 210–241aa (tau5) mAb (scFV) JNPL3 [a252]
tau 306-320aa repeat domain Ab (HJ9.3) PS19 [a130]
tau 312-322aa (MC-1) IgG JNPL3 [a268]
tau 312-322aa (MC-1) IgG hTau.P301S [a268]
tau 312-322aa (MC-1) IgG rTg4510 [a201]
tau 312-322aa (MC-1) IgG1 JNPL3 [a202]
tau 312-322aa (MC1) Ab (scFv) -AAV1 JNPL3 [a269]
tau 312-322aa (MC1) Ab (scFv) -AAV1 hTau.P301S [a269]
tau 312-322aa (MC1) Ab (scFv) -AAV5 JNPL3 [a270]
tau 7-13aa (HJ9.4) Ab PS19 [a130]
tau 84-97aa RN2N IgG1 pR5 [a258]
tau 84-97aa RN2N IgG2a pR5 [a258]
tau acK280 Ab (Y01) JNPL3 [a259]
tau acK280 Ab (Y01) PS19 [a259]
tau cis-pT231 mAb (PNT001) rTg4510 [a261]
tau oligomer mAb (TOMA) JNPL3 [a273]
tau oligomer mAb (TOMA) PS19 [a274]
tau oligomer mAb (TOMA) rTg4510 [a275]
tau oligomer mAb (TOMA1) hTau [a276]
tau oligomer mAb (TOMA1) JNPL3 [a276]
tau oligomer mAb (TOMA3) hTau [a276]
tau oligomer mAb (TOMA3) JNPL3 [a276]
tau pS202 (CP13) mAb JNPL3 [a278]
tau pS202 (CP13) mAb (intrabody) JNPL3 [a252]
tau pS202 (CP13) mAb (intrabody) rTg4510 [a252]
tau pS202 (CP13) mAb (scFV) JNPL3 [a252]
tau pS202 (CP13) mAb (scFV) rTg4510 [a252]
tau pS396 (PHF13.6) IgG rTg4510 [a279]
tau pS396 (Ta4) IgG2b Tau784 [a280]
tau pS396 (Ta9) IgG3 Tau609 [a280]
tau pS396 (Ta9) IgG3 Tau784 [a280]
tau pS396/404 (4E6G7) mAb JNPL3 [a281]
tau pS396/404 (PHF-1) IgG JNPL3 [a282]
tau pS396/404 (PHF1) IgG (4E6G7) (phospho-selective) hTau [a283]
tau pS396/404 (PHF1) IgG (6B2G12) (conformational) hTau [a283]
tau pS396/404 (PHF1) IgG1 JNPL3 [a202]
tau pS396/404 (PHF1) mAb (intrabody) JNPL3 [a252]
tau pS396/404 (PHF1) mAb (intrabody) rTg4510 [a252]
tau pS396/404(PHF-1)-AAVrh.10 hTau.P301S [a284]
tau pS396/S404 (PHF-1) IgG rTg4510 [a201]
tau pS396/S404 (PHF1) IgG JNPL3 [a268]
tau pS396/S404 (PHF1) IgG hTau.P301S [a268]
tau pS396/S404 (PHF1) mAb (scFv) JNPL3 [a252]
tau pS396/S404 (PHF1) mAb (scFv) rTg4510 [a252]
tau pS404 IgG1 (mAb1) K3 [a203]
tau pS404 IgG1 (mAb1) pR5 [a203]
tau pS404 IgG2a (mAb2) K3 [a203]
tau pS404 IgG2a (mAb2) pR5 [a203]
tau pS409 (PG5) mAb JNPL3 [a278]
tau pS409 IgG2 effectorless mutant pR5 [a285]
tau pS409 IgG2a WT pR5 [a285]
tau pS413 (Ta1505) IgG2a Tau784 [a280]
tau pT231 (PHF6.10) IgG rTg4510 [a279]
tau pT231 (RZ3) mAb JNPL3 [a278]
tau RN2N Ab (scFv) + scanning ultrasound pR5 [a254]
tau3R Ab scFv-ApoB-LV hTau 3R (line 13) [a290]
tau3R Ab scFv-LV hTau 3R (line 13) [a290]

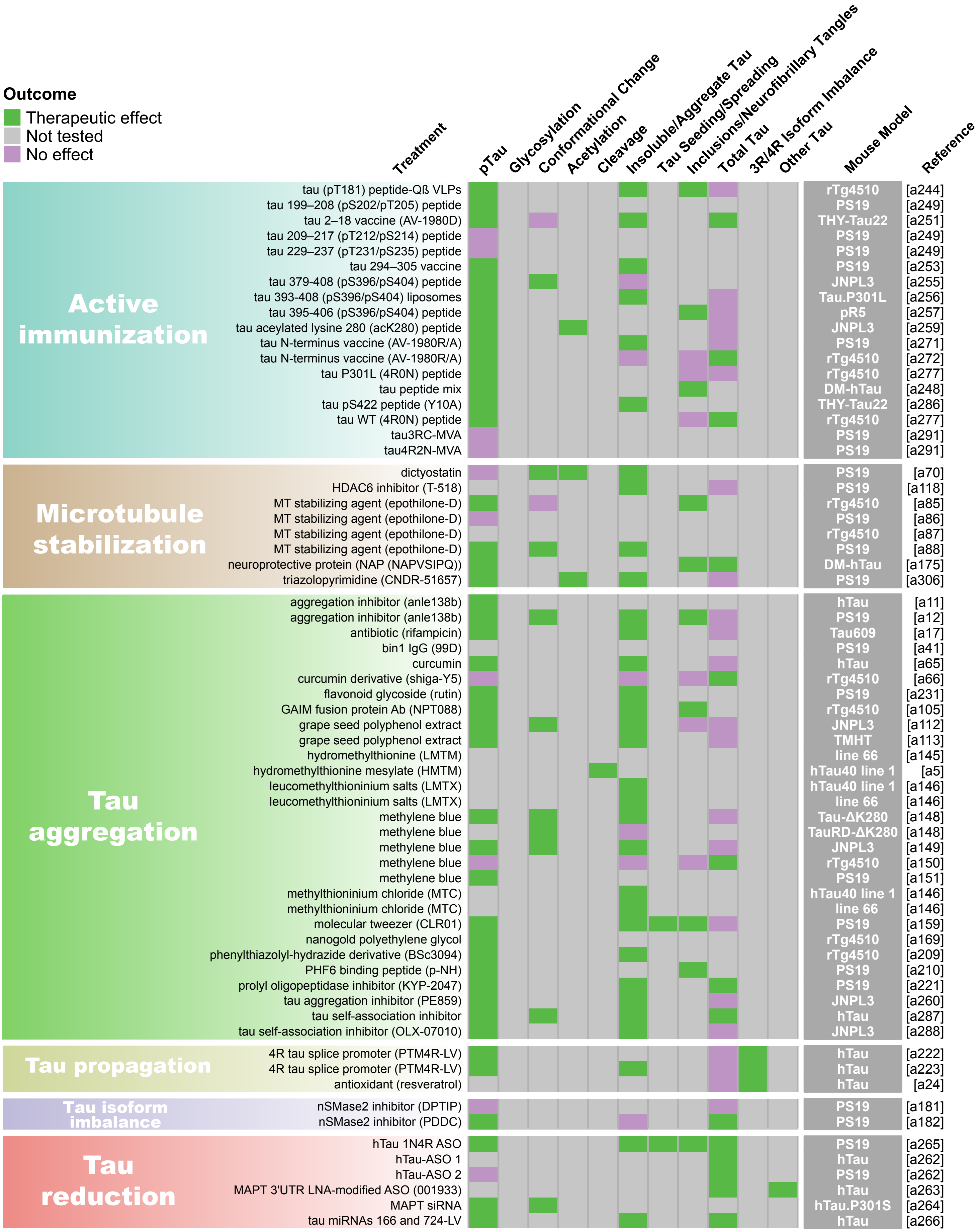
Outcome
Therapeutic effect
Not tested
No effect
Treatment
pTau
Glycosylation
Conformational Change
Acetylation
Cleavage
Insoluble/Aggregate Tau
Tau Seeding/Spreading
Inclusions/Neurofibrillary Tangles
Total Tau
3R/4R Isoform Imbalance
Other Tau
Mouse Model
Reference
Active immunization
tau (pT181) peptide-Qß VLPs rTg4510 [a244]
tau 199–208 (pS202/pT205) peptide PS19 [a249]
tau 2–18 vaccine (AV-1980D) THY-Tau22 [a251]
tau 209–217 (pT212/pS214) peptide PS19 [a249]
tau 229–237 (pT231/pS235) peptide PS19 [a249]
tau 294–305 vaccine PS19 [a253]
tau 379-408 (pS396/pS404) peptide JNPL3 [a255]
tau 393-408 (pS396/pS404) liposomes Tau.P301L [a256]
tau 395-406 (pS396/pS404) peptide pR5 [a257]
tau aceylated lysine 280 (acK280) peptide JNPL3 [a259]
tau N-terminus vaccine (AV-1980R/A) PS19 [a271]
tau N-terminus vaccine (AV-1980R/A) rTg4510 [a272]
tau P301L (4R0N) peptide rTg4510 [a277]
tau peptide mix DM-hTau [a248]
tau pS422 peptide (Y10A) THY-Tau22 [a286]
tau WT (4R0N) peptide rTg4510 [a277]
tau3RC-MVA PS19 [a291]
tau4R2N-MVA PS19 [a291]
Microtubule stabilization
dictyostatin PS19 [a70]
HDAC6 inhibitor (T-518) PS19 [a118]
MT stabilizing agent (epothilone-D) rTg4510 [a85]
MT stabilizing agent (epothilone-D) PS19 [a86]
MT stabilizing agent (epothilone-D) rTg4510 [a87]
MT stabilizing agent (epothilone-D) PS19 [a88]
neuroprotective protein (NAP (NAPVSIPQ)) DM-hTau [a175]
triazolopyrimidine (CNDR-51657) PS19 [a306]
Tau aggregation
aggregation inhibitor (anle138b) hTau [a11]
aggregation inhibitor (anle138b) PS19 [a12]
antibiotic (rifampicin) Tau609 [a17]
bin1 IgG (99D) PS19 [a41]
curcumin hTau [a65]
curcumin derivative (shiga-Y5) rTg4510 [a66]
flavonoid glycoside (rutin) PS19 [a231]
GAIM fusion protein Ab (NPT088) rTg4510 [a105]
grape seed polyphenol extract JNPL3 [a112]
grape seed polyphenol extract TMHT [a113]
hydromethylthionine (LMTM) line 66 [a145]
hydromethylthionine mesylate (HMTM) hTau40 line 1 [a5]
leucomethylthioninium salts (LMTX) hTau40 line 1 [a146]
leucomethylthioninium salts (LMTX) line 66 [a146]
methylene blue Tau-ΔK280 [a148]
methylene blue TauRD-ΔK280 [a148]
methylene blue JNPL3 [a149]
methylene blue rTg4510 [a150]
methylene blue PS19 [a151]
methylthioninium chloride (MTC) hTau40 line 1 [a146]
methylthioninium chloride (MTC) line 66 [a146]
molecular tweezer (CLR01) PS19 [a159]
nanogold polyethylene glycol rTg4510 [a169]
phenylthiazolyl-hydrazide derivative (BSc3094) rTg4510 [a209]
PHF6 binding peptide (p-NH) PS19 [a210]
prolyl oligopeptidase inhibitor (KYP-2047) PS19 [a221]
tau aggregation inhibitor (PE859) JNPL3 [a260]
tau self-association inhibitor hTau [a287]
tau self-association inhibitor (OLX-07010) JNPL3 [a288]
Tau propagation
4R tau splice promoter (PTM4R-LV) hTau [a222]
4R tau splice promoter (PTM4R-LV) hTau [a223]
antioxidant (resveratrol) hTau [a24]
Tau isoform imbalance
nSMase2 inhibitor (DPTIP) PS19 [a181]
nSMase2 inhibitor (PDDC) PS19 [a182]
Tau reduction
hTau 1N4R ASO PS19 [a265]
hTau-ASO 1 hTau [a262]
hTau-ASO 2 PS19 [a262]
MAPT 3'UTR LNA-modified ASO (001933) hTau [a263]
MAPT siRNA hTau.P301S [a264]
tau miRNAs 166 and 724-LV hTau [a266]

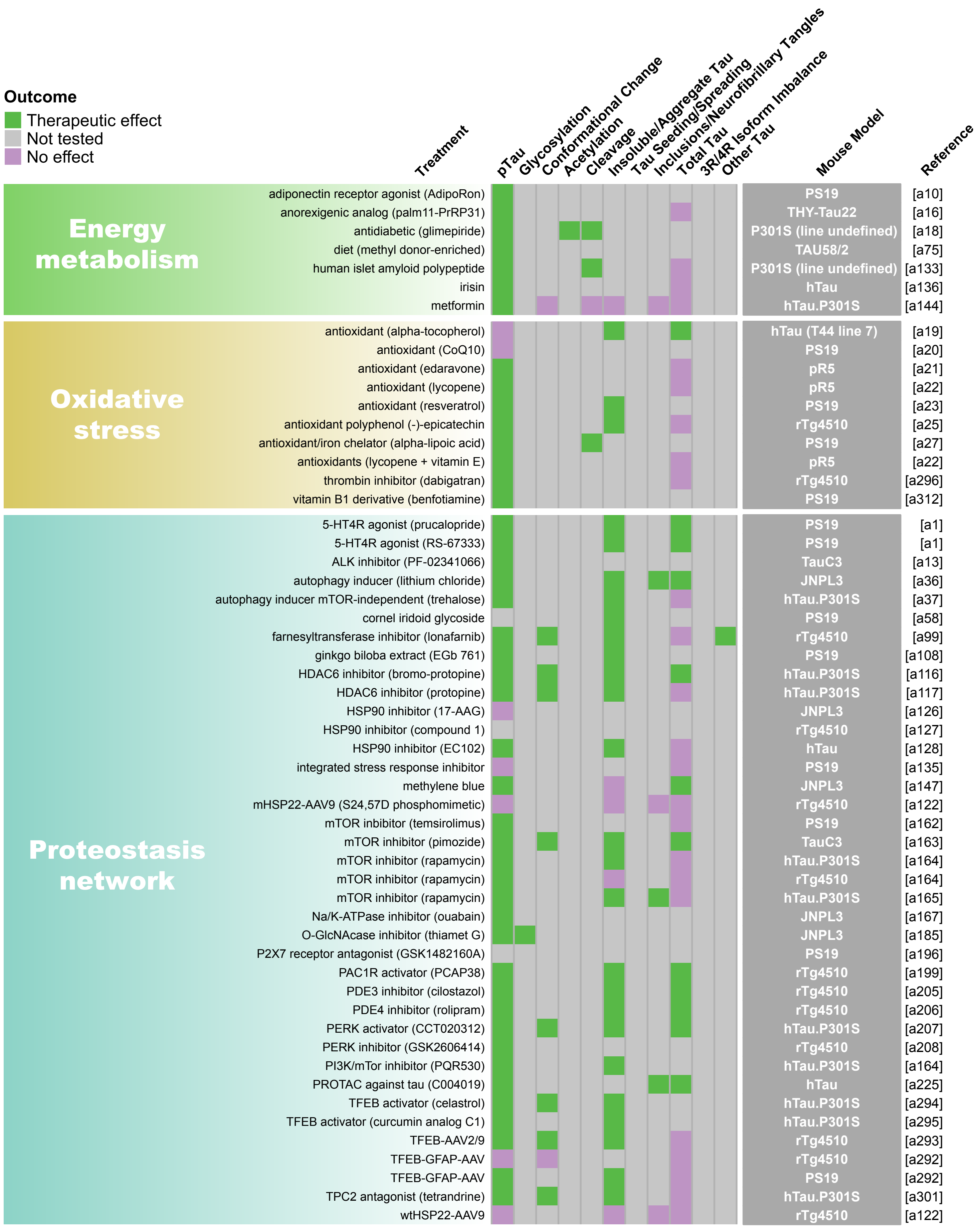

Outcome
Therapeutic effect
Not tested
No effect
Treatment
pTau
Glycosylation
Conformational Change
Acetylation
Cleavage
Insoluble/Aggregate Tau
Tau Seeding/Spreading
Inclusions/Neurofibrillary Tangles
Total Tau
3R/4R Isoform Imbalance
Other Tau
Mouse Model
Reference
Energy metabolism
adiponectin receptor agonist (AdipoRon) PS19 [a10]
anorexigenic analog (palm11-PrRP31) THY-Tau22 [a16]
antidiabetic (glimepiride) P301S (line undefined) [a18]
diet (methyl donor-enriched) TAU58/2 [a75]
human islet amyloid polypeptide P301S (line undefined) [a133]
irisin hTau [a136]
metformin hTau.P301S [a144]
Oxidative stress
antioxidant (alpha-tocopherol) hTau (T44 line 7) [a19]
antioxidant (CoQ10) PS19 [a20]
antioxidant (edaravone) pR5 [a21]
antioxidant (lycopene) pR5 [a22]
antioxidant (resveratrol) PS19 [a23]
antioxidant polyphenol (-)-epicatechin rTg4510 [a25]
antioxidant/iron chelator (alpha-lipoic acid) PS19 [a27]
antioxidants (lycopene + vitamin E) pR5 [a22]
thrombin inhibitor (dabigatran) rTg4510 [a296]
vitamin B1 derivative (benfotiamine) PS19 [a312]
Proteostasis network
5-HT4R agonist (prucalopride) PS19 [a1]
5-HT4R agonist (RS-67333) PS19 [a1]
ALK inhibitor (PF-02341066) TauC3 [a13]
autophagy inducer (lithium chloride) JNPL3 [a36]
autophagy inducer mTOR-independent (trehalose) hTau.P301S [a37]
cornel iridoid glycoside PS19 [a58]
farnesyltransferase inhibitor (lonafarnib) rTg4510 [a99]
ginkgo biloba extract (EGb 761) PS19 [a108]
HDAC6 inhibitor (bromo-protopine) hTau.P301S [a116]
HDAC6 inhibitor (protopine) hTau.P301S [a117]
HSP90 inhibitor (17-AAG) JNPL3 [a126]
HSP90 inhibitor (compound 1) rTg4510 [a127]
HSP90 inhibitor (EC102) hTau [a128]
integrated stress response inhibitor PS19 [a135]
methylene blue JNPL3 [a147]
mHSP22-AAV9 (S24,57D phosphomimetic) rTg4510 [a122]
mTOR inhibitor (temsirolimus) PS19 [a162]
mTOR inhibitor (pimozide) TauC3 [a163]
mTOR inhibitor (rapamycin) hTau.P301S [a164]
mTOR inhibitor (rapamycin) rTg4510 [a164]
mTOR inhibitor (rapamycin) hTau.P301S [a165]
Na/K-ATPase inhibitor (ouabain) JNPL3 [a167]
O-GlcNAcase inhibitor (thiamet G) JNPL3 [a185]
P2X7 receptor antagonist (GSK1482160A) PS19 [a196]
PAC1R activator (PCAP38) rTg4510 [a199]
PDE3 inhibitor (cilostazol) rTg4510 [a205]
PDE4 inhibitor (rolipram) rTg4510 [a206]
PERK activator (CCT020312) hTau.P301S [a207]
PERK inhibitor (GSK2606414) rTg4510 [a208]
PI3K/mTor inhibitor (PQR530) hTau.P301S [a164]
PROTAC against tau (C004019) hTau [a225]
TFEB activator (celastrol) hTau.P301S [a294]
TFEB activator (curcumin analog C1) hTau.P301S [a295]
TFEB-AAV2/9 rTg4510 [a293]
TFEB-GFAP-AAV rTg4510 [a292]
TFEB-GFAP-AAV PS19 [a292]
TPC2 antagonist (tetrandrine) hTau.P301S [a301]
wtHSP22-AAV9 rTg4510 [a122]

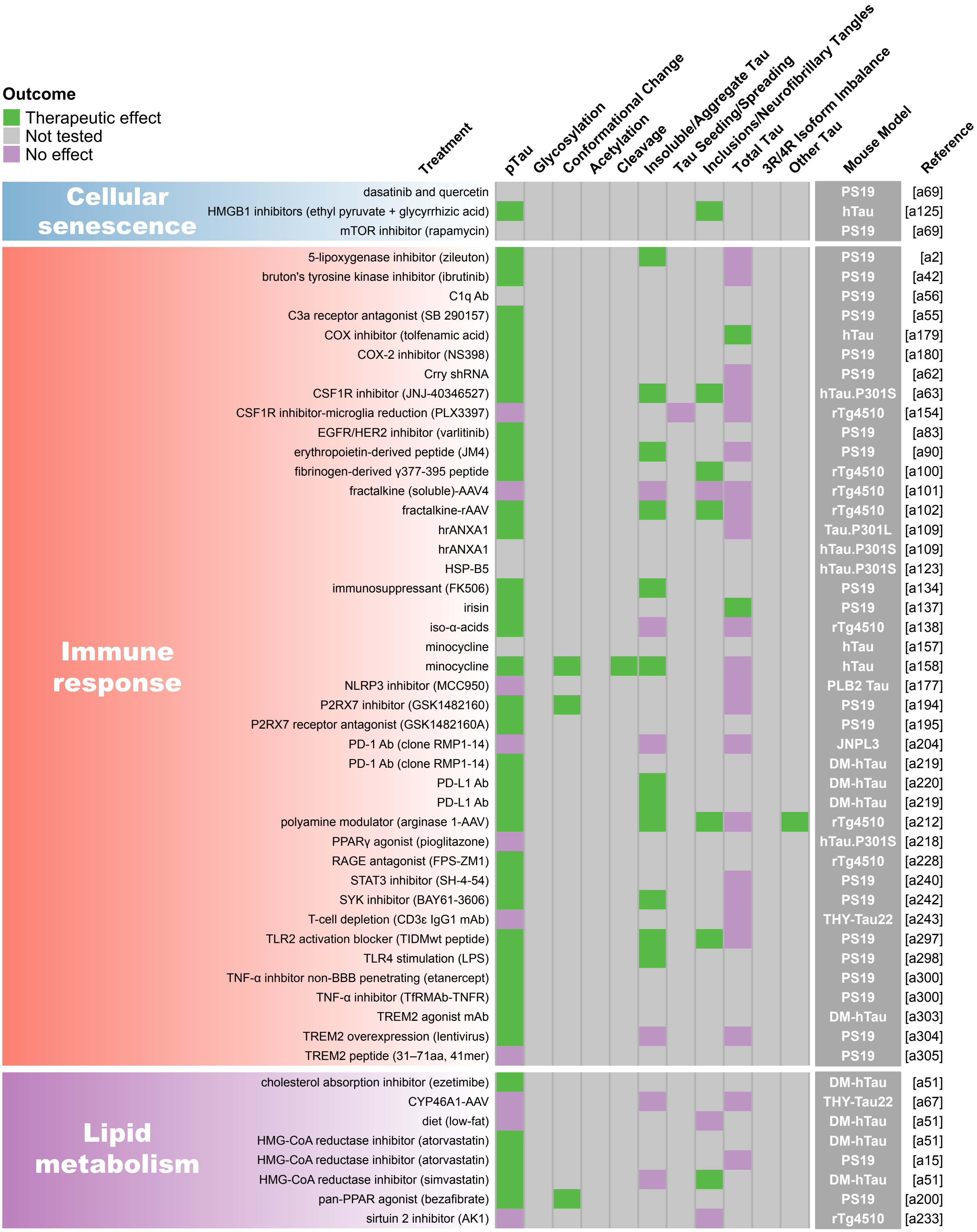

Outcome
Therapeutic effect
Not tested
No effect
Treatment
pTau
Glycosylation
Conformational Change
Acetylation
Cleavage
Insoluble/Aggregate Tau
Tau Seeding/Spreading
Inclusions/Neurofibrillary Tangles
Total Tau
3R/4R Isoform Imbalance
Other Tau
Mouse Model
Reference
Cellular senescence
dasatinib and quercetin
PS19
[a69]
HMGB1 inhibitors (ethyl pyruvate + glycyrrhizic acid)
hTau
[a125]
mTOR inhibitor (rapamycin)
PS19
[a69]
Immune response
5-lipoxygenase inhibitor (zileuton)
PS19
[a2]
bruton's tyrosine kinase inhibitor (ibrutinib)
PS19
[a42]
C1q Ab
PS19
[a56]
C3a receptor antagonist (SB 290157)
PS19
[a55]
COX inhibitor (tolfenamic acid)
hTau
[a179]
COX-2 inhibitor (NS398)
PS19
[a180]
Crry shRNA
PS19
[a62]
CSF1R inhibitor (JNJ-40346527)
hTau.P301S
[a63]
CSF1R inhibitor-microglia reduction (PLX3397)
rTg4510
[a154]
EGFR/HER2 inhibitor (varlitinib)
PS19
[a83]
erythropoietin-derived peptide (JM4)
PS19
[a90]
fibrinogen-derived γ377-395 peptide
rTg4510
[a100]
fractalkine (soluble)-AAV4
rTg4510
[a101]
fractalkine-rAAV
rTg4510
[a102]
hrANXA1
Tau.P301L
[a109]
hrANXA1
hTau.P301S
[a109]
HSP-B5
hTau.P301S
[a123]
immunosuppressant (FK506)
PS19
[a134]
irisin
PS19
[a137]
iso-α-acids
rTg4510
[a138]
minocycline
hTau
[a157]
minocycline
hTau
[a158]
NLRP3 inhibitor (MCC950)
PLB2 Tau
[a177]
P2RX7 inhibitor (GSK1482160)
PS19
[a194]
P2RX7 receptor antagonist (GSK1482160A)
PS19
[a195]
PD-1 Ab (clone RMP1-14)
JNPL3
[a204]
PD-1 Ab (clone RMP1-14)
DM-hTau
[a219]
PD-L1 Ab
DM-hTau
[a220]
PD-L1 Ab
DM-hTau
[a219]
polyamine modulator (arginase 1-AAV)
rTg4510
[a212]
PPARγ agonist (pioglitazone)
hTau.P301S
[a218]
RAGE antagonist (FPS-ZM1)
rTg4510
[a228]
STAT3 inhibitor (SH-4-54)
PS19
[a240]
SYK inhibitor (BAY61-3606)
PS19
[a242]
T-cell depletion (CD3ε IgG1 mAb)
THY-Tau22
[a243]
TLR2 activation blocker (TIDMwt peptide)
PS19
[a297]
TLR4 stimulation (LPS)
PS19
[a298]
TNF-α inhibitor non-BBB penetrating (etanercept)
PS19
[a300]
TNF-α inhibitor (TfRMAb-TNFR)
PS19
[a300]
TREM2 agonist mAb
DM-hTau
[a303]
TREM2 overexpression (lentivirus)
PS19
[a304]
TREM2 peptide (31–71aa, 41mer)
PS19
[a305]
Lipid metabolism
cholesterol absorption inhibitor (ezetimibe)
DM-hTau
[a51]
CYP46A1-AAV
THY-Tau22
[a67]
diet (low-fat)
DM-hTau
[a51]
HMG-CoA reductase inhibitor (atorvastatin)
DM-hTau
[a51]
HMG-CoA reductase inhibitor (atorvastatin)
PS19
[a15]
HMG-CoA reductase inhibitor (simvastatin)
DM-hTau
[a51]
pan-PPAR agonist (bezafibrate)
PS19
[a200]
sirtuin 2 inhibitor (AK1)
rTg4510
[a233]

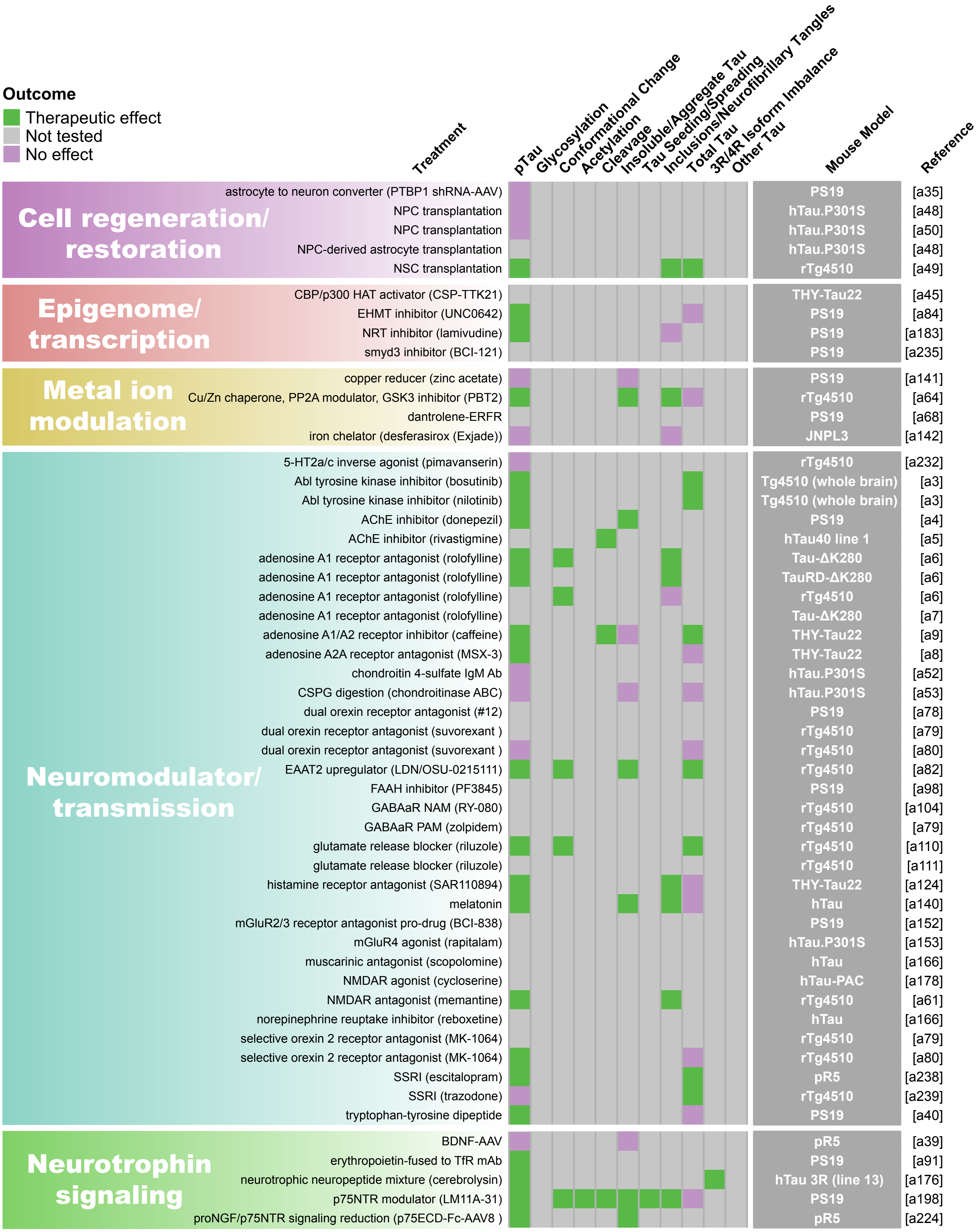
Outcome
Therapeutic effect
Not tested
No effect
Treatment
pTau
Glycosylation
Conformational Change
Acetylation
Cleavage
Insoluble/Aggregate Tau
Tau Seeding/Spreading
Inclusions/Neurofibrillary Tangles
Total Tau
3R/4R Isoform Imbalance
Other Tau
Mouse Model
Reference
Cell regeneration/ restoration
astrocyte to neuron converter (PTBP1 shRNA-AAV) PS19 [a35]
NPC transplantation hTau.P301S [a48]
NPC transplantation hTau.P301S [a50]
NPC-derived astrocyte transplantation hTau.P301S [a48]
NSC transplantation rTg4510 [a49]
Epigenome/ transcription
CBP/p300 HAT activator (CSP-TTK21) THY-Tau22 [a45]
EHMT inhibitor (UNC0642) PS19 [a84]
NRT inhibitor (lamivudine) PS19 [a183]
smyd3 inhibitor (BCI-121) PS19 [a235]
Metal ion modulation
copper reducer (zinc acetate) PS19 [a141]
Cu/Zn chaperone, PP2A modulator, GSK3 inhibitor (PBT2) rTg4510 [a64]
dantrolene-ERFR PS19 [a68]
iron chelator (desferasirox (Exjade)) JNPL3 [a142]
Neuromodulator/ transmission
5-HT2a/c inverse agonist (pimavanserin) rTg4510 [a232]
Abl tyrosine kinase inhibitor (bosutinib) Tg4510 (whole brain) [a3]
Abl tyrosine kinase inhibitor (nilotinib) Tg4510 (whole brain) [a3]
AChE inhibitor (donepezil) PS19 [a4]
AChE inhibitor (rivastigmine) hTau40 line 1 [a5]
adenosine A1 receptor antagonist (rolofylline) Tau-ΔK280 [a6]
adenosine A1 receptor antagonist (rolofylline) TauRD-ΔK280 [a6]
adenosine A1 receptor antagonist (rolofylline) rTg4510 [a6]
adenosine A1 receptor antagonist (rolofylline) Tau-ΔK280 [a7]
adenosine A1/A2 receptor inhibitor (caffeine) THY-Tau22 [a9]
adenosine A2A receptor antagonist (MSX-3) THY-Tau22 [a8]
chondroitin 4-sulfate IgM Ab hTau.P301S [a52]
CSPG digestion (chondroitinase ABC) hTau.P301S [a53]
dual orexin receptor antagonist (#12) PS19 [a78]
dual orexin receptor antagonist (suvorexant) rTg4510 [a79]
dual orexin receptor antagonist (suvorexant) rTg4510 [a80]
EAAT2 upregulator (LDN/OSU-0215111) rTg4510 [a82]
FAAH inhibitor (PF3845) PS19 [a98]
GABAaR NAM (RY-080) rTg4510 [a104]
GABAaR PAM (zolpidem) rTg4510 [a79]
glutamate release blocker (riluzole) rTg4510 [a110]
glutamate release blocker (riluzole) rTg4510 [a111]
histamine receptor antagonist (SAR110894) THY-Tau22 [a124]
melatonin hTau [a140]
mGluR2/3 receptor antagonist pro-drug (BCI-838) PS19 [a152]
mGluR4 agonist (rapitalam) hTau.P301S [a153]
muscarinic antagonist (scopolomine) hTau [a166]
NMDAR agonist (cycloserine) hTau-PAC [a178]
NMDAR antagonist (memantine) rTg4510 [a61]
norepinephrine reuptake inhibitor (reboxetine) hTau [a166]
selective orexin 2 receptor antagonist (MK-1064) rTg4510 [a79]
selective orexin 2 receptor antagonist (MK-1064) rTg4510 [a80]
SSRI (escitalopram) pR5 [a238]
SSRI (trazodone) rTg4510 [a239]
tryptophan-tyrosine dipeptide PS19 [a40]
Neurotrophin signaling
BDNF-AAV pR5 [a39]
erythropoietin-fused to TfR mAb PS19 [a91]
neurotrophic neuropeptide mixture (cerebrolysin) hTau 3R (line 13) [a176]
p75NTR modulator (LM11A-31) PS19 [a198]
proNGF/p75NTR signaling reduction (p75ECD-Fc-AAV8) pR5 [a224]

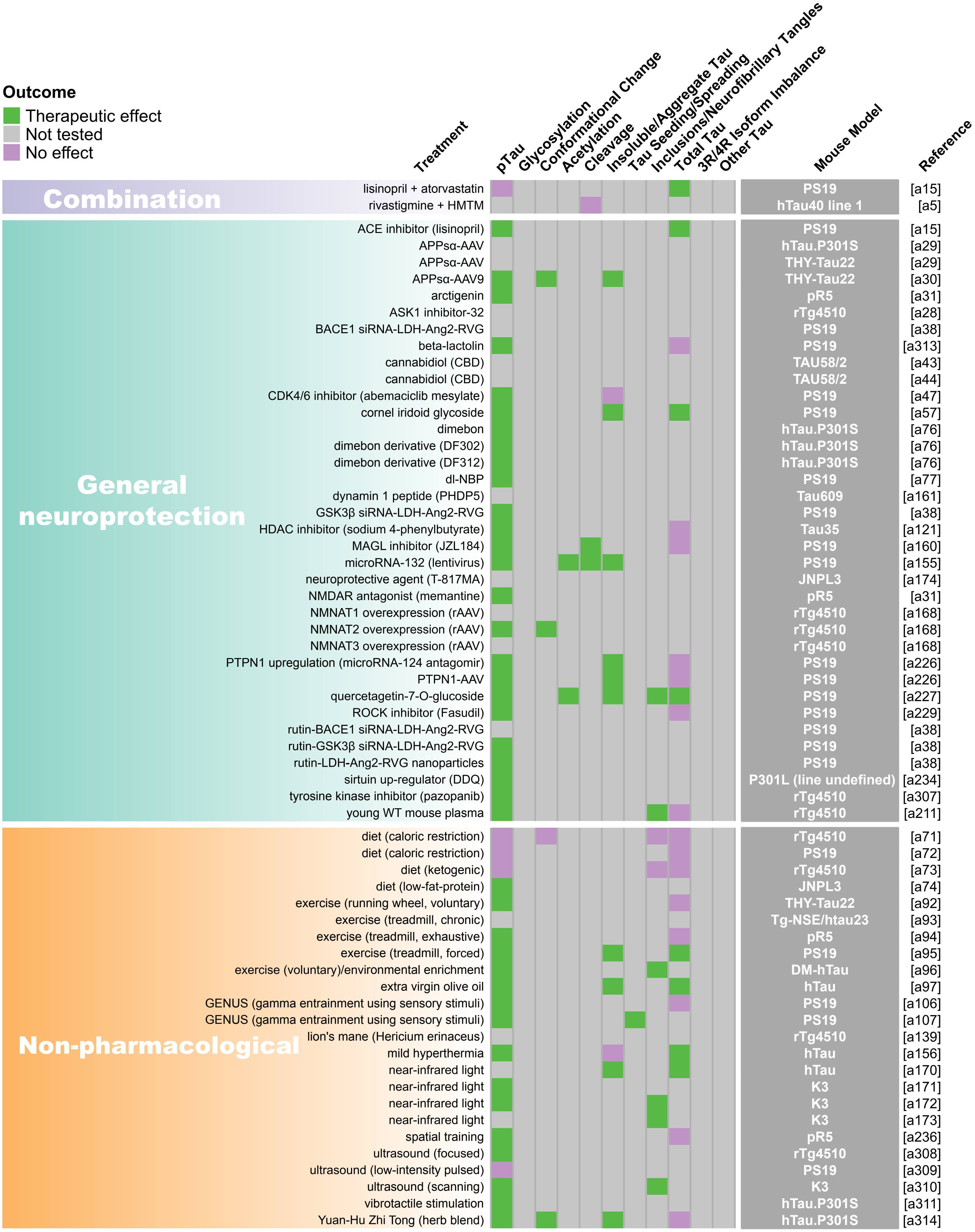

Outcome
Therapeutic effect
Not tested
No effect
Treatment
pTau
Glycosylation
Conformational Change
Acetylation
Cleavage
Insoluble/Aggregate Tau
Tau Seeding/Spreading
Inclusions/Neurofibrillary Tangles
Total Tau
3R/4R Isoform Imbalance
Other Tau
Mouse Model
Reference
Combination
lisinopril + atorvastatin PS19 [a15]
rivastigmine + HMTM hTau40 line 1 [a5]
General neuroprotection
ACE inhibitor (lisinopril) PS19 [a15]
APPsα-AAV hTau.P301S [a29]
APPsα-AAV THY-Tau22 [a29]
APPsα-AAV9 THY-Tau22 [a30]
arctigenin pR5 [a31]
ASK1 inhibitor-32 rTg4510 [a28]
BACE1 siRNA-LDH-Ang2-RVG PS19 [a38]
beta-lactolin PS19 [a313]
cannabidiol (CBD) TAU58/2 [a43]
cannabidiol (CBD) TAU58/2 [a44]
CDK4/6 inhibitor (abemaciclib mesylate) PS19 [a47]
cornel iridoid glycoside PS19 [a57]
dimebon hTau.P301S [a76]
dimebon derivative (DF302) hTau.P301S [a76]
dimebon derivative (DF312) hTau.P301S [a76]
dl-NBP PS19 [a77]
dynamin 1 peptide (PHDP5) Tau609 [a161]
GSK3β siRNA-LDH-Ang2-RVG PS19 [a38]
HDAC inhibitor (sodium 4-phenylbutyrate) Tau35 [a121]
MAGL inhibitor (JZL184) PS19 [a160]
microRNA-132 (lentivirus) PS19 [a155]
neuroprotective agent (T-817MA) JNPL3 [a174]
NMDAR antagonist (memantine) pR5 [a31]
NMNAT1 overexpression (rAAV) rTg4510 [a168]
NMNAT2 overexpression (rAAV) rTg4510 [a168]
NMNAT3 overexpression (rAAV) rTg4510 [a168]
PTPN1 upregulation (microRNA-124 antagomir) PS19 [a226]
PTPN1-AAV PS19 [a226]
quercetagetin-7-O-glucoside PS19 [a227]
ROCK inhibitor (Fasudil) PS19 [a229]
rutin-BACE1 siRNA-LDH-Ang2-RVG PS19 [a38]
rutin-GSK3β siRNA-LDH-Ang2-RVG PS19 [a38]
rutin-LDH-Ang2-RVG nanoparticles PS19 [a38]
sirtuin up-regulator (DDQ) P301L (line undefined) [a234]
tyrosine kinase inhibitor (pazopanib) rTg4510 [a307]
young WT mouse plasma rTg4510 [a211]
Non-pharmacological
diet (caloric restriction) rTg4510 [a71]
diet (caloric restriction) PS19 [a72]
diet (ketogenic) rTg4510 [a73]
diet (low-fat-protein) JNPL3 [a74]
exercise (running wheel, voluntary) THY-Tau22 [a92]
exercise (treadmill, chronic) Tg-NSE/htau23 [a93]
exercise (treadmill, exhaustive) pR5 [a94]
exercise (treadmill, forced) PS19 [a95]
exercise (voluntary)/environmental enrichment DM-hTau [a96]
extra virgin olive oil hTau [a97]
GENUS (gamma entrainment using sensory stimuli) PS19 [a106]
GENUS (gamma entrainment using sensory stimuli) PS19 [a107]
lion's mane (Hericium erinaceus) rTg4510 [a139]
mild hyperthermia hTau [a156]
near-infrared light hTau [a170]
near-infrared light K3 [a171]
near-infrared light K3 [a172]
near-infrared light K3 [a173]
spatial training pR5 [a236]
ultrasound (focused) rTg4510 [a308]
ultrasound (low-intensity pulsed) PS19 [a309]
ultrasound (scanning) K3 [a310]
vibrotactile stimulation hTau.P301S [a311]
Yuan-Hu Zhi Tong (herb blend) hTau.P301S [a314]

Heatmap showing all 409 treatment evaluations included in this review and the effect observed on each pathological tau endpoint. The evaluations are grouped by therapeutic strategy. In the endpoint columns, green indicates that a therapeutic effect on the specified endpoint was reported, pink indicates that the endpoint was assessed but there was no therapeutic effect, and grey indicates that the endpoint was not tested. The dark gray column provides the name/line of MAPT mouse model used for each evaluation (see Table S3 for a full description of each model). Abbreviations are defined in Table S4.The last column provides the reference for each evaluation **(Supplemental References 1).**
