# Supplementary material for: Twenty years of therapeutic development in tauopathy mouse models: a scoping review
Source: Alzheimers Dement. 2025 Aug 18;21(8):e70578. doi: 10.1002/alz.70578 (PMC12360913; doi:10.1002/alz.70578)
Supplement: Supplementary file 6 — Supporting Information [file ALZ-21-e70578-s001.docx]

| Table S2. Summary of major endpoint categories used in the included studies and their associated assays. | |
| --- | --- |
| Pathological tau endpoints | **Measurements/Assays** |
| Phosphorylated tau | AT8 (pS202/pT205), PHF-1 (pS396/pS404), AT100 (pT212/pS214), AT180 (pT231), AT270 (pT181), CP9 (pT231), CP13 (pS202), Ta1505 (pT413), pS199, pS202, pS262, pS356, pS396, pS404, pS409, pS422, pS433, pT205, pT181, pT231, pY18, tau1 (dephosphorylated tau) |
| Glycosylation | CTD110.6, RL2, 1F5.D6, 9D1.E4, 18B10C7, 6D93 |
| Acetylation | K174, K274, K280, K281, KIGS motif (acetylated lysine residues K259 and K353) |
| Conformational change | MC1, Alz50, TTCM2 |
| Cleavage | Caspase 3, Caspase 7, Calpain 2, Tau-C3, cathepsin |
| Insoluble/oligomerization | Sarkosyl-insoluble fraction, RIPA-insoluble fraction (70% formic acid extract), T22, Toc1, 64 kDa tau, HMW bands from total tau antibodies, HTRF assay for tau aggregates, fibrillar oligomer-specific OC antibody |
| Seeding activity | In vitro seeding activity blocked by brain extracts from treated mice, depletion of transgenic homogenate with antibody therapeutic followed by in vitro seeding assay |
| Tau inclusions/neurofibrillary tangles | Thioflavin S, Gallyas silver, Bielschowsky silver, PG5, nY29, NFT specific antibody RN235 |
| Total tau | Soluble or whole cell lysates from CNS derived tissues analyzed by western, ELISA, or IF probed with total tau/pan-tau/phosphorylation independent antibodies (Tau-5, HT7, 3G6, H-150, Tau 46, tau-13, Tau E1, pan-tau, DA31, E178, K9JA, Tau Y9, T49, BR133, BR134 ) |
| 3R/4R tau imbalance | RD3 (3R tau) and RD4 (4R tau) antibodies, RT-PCR (exon 10 inclusion/exclusion) |
|  |  |
| Cellular, systemic, and functional endpoints | **Measurements/Assays** |
| Tau modifying enzymes | CDK5, GSK3, ERK, SAPK/JNK, MAPK, O-GlcNAcase, MARK4 (tau kinase), PP2A, DYRK1A, FYN kinase, TTBK1 |
| Neuron/volume loss | Volume, Area, Cell layer thickness, NeuN count, DAPI count, Cresyl violet/Nissl count, TUNEL, Cell density, Brain region weight |
| Synaptic degeneration | Synaptophysin, Synapsin, PSD95, PSD93, SNL1, NR1, Spine density, Cupric silver, SV2A/B, SNAP-25, Munc18-1, MAP2, NR2B, GluR1, Homer, Bassoon, # of synapses by TEM, Electron microscopy, Axonal dystrophy, Syntaxin1A, Synaptotagmin, GluA2/N1/N2B |
| Synaptic dysfunction | Long-term potentiation, Long-term depression, Paired-pulse facilitation, Basal synaptic transmission, Synaptic-driven spontaneous action potential (sAP), Evoked action potential (eAP), EPSC, Firing rate, Glutamate transients, Ca2+ influx |
| Inflammation | Inflammatory interleukins, Iba1, CD68, CD45, CD11, HLA-D, HLA-DR, GFAP, iNOS, Arg1, Mrc1, C1q, Complement proteins (C3, C3B), mRNA, Inflammatory cytokines (IL-1, IL-4, IL-6, IL-12, IL-18, IL-1B, Cox2, TNF⍺, NFkB, NLRP3, IFN𝛾) |
| Autophagy/UPS | p62/SQSTM1, LC3 II/B, LAMP1, BECN1, Cathepsin D, Chaperone mediated autophagy markers (LAMP2A), mTor |
| Cognitive deficits | Novel object/location/mouse recognition, Morris water maze, Y-maze, Fear conditioning, Barnes/Radial arm water maze, Olfactory memory (Familiar odor discrimination), Cheeseboard maze, Contextual fear conditioning, T-Maze |
| BPSD/ADL | BPSD: aggression/agitation (resident-intruder task), depressive-like behavior (forced swim test, tail suspension test, apathy-like behavior (object exploration, social withdrawal (approach behaviour to conspecifics: free-roaming or three-chamber sociability test)), anxiety-like behavior (time spent in open-field, elevated plus maze, light-dark box, elevated zero maze), sleep/wake disturbances (electroencephalography (EEG), electromyography (EMG), running wheels, infrared beams, and piezoelectric systems) ADL: care of the self, social and domestic environments: (Burrowing, nest building, hoarding); Neuropsychiatric: pre-pulse inhibition |
| Motor deficits | Rotarod, Traverse beam, Horizontal bar, Inverted screen, Ledge test, Ladder test, Clasping, Open field activity (distance traveled), General locomotor activity, Distance traveled, Mean speed, Parallel rod floor, Balance beam, Tail hanging, Pole test |
| Response biomarkers | Plasma / Urine / CSF tau biomarkers, PET, MRI |
| Survival | Kaplan-Meier analysis, % survival, % mortality |
