# Supplementary material for: Twenty years of therapeutic development in tauopathy mouse models: a scoping review
Source: Alzheimers Dement. 2025 Aug 18;21(8):e70578. doi: 10.1002/alz.70578 (PMC12360913; doi:10.1002/alz.70578)
Supplement: Supplementary file 8 — Supporting Information [file ALZ-21-e70578-s007.docx]

| Table S4: Supplemental abbreviations for Figures 2, S1; Table 1 | |
| --- | --- |
| Abbreviation | **Definition** |
| 5-HT | 5-hydroxytryptamine/serotonin |
| AAV | adeno-associated virus |
| ACE | angiotensin-converting enzyme |
| AChE | acetylcholinesterase |
| acK | aceylated lysine |
| AEP | asparagine endopeptidase |
| ALK | anaplastic lymphoma kinase |
| AMPK | AMP-activated protein kinase |
| APPsα | APP ectodomain cleaved by α-secretase |
| ASK1 | apoptosis signal-regulating kinase 1 |
| ASO | antisense oligonucleotide |
| AV | Adenovirus |
| BACE1 | β-site APP cleaving enzyme 1 |
| BDNF | brain-derived neurotrophic factor |
| bin1 | bridging integrator 1 |
| Ca | calcium |
| CBP | CREB-binding protein |
| CoQ10 | coenzyme Q10 |
| COX | cyclooxygenase |
| Crry | complement component (3b/4b) receptor 1-related protein Y |
| CSF1R | colony-stimulating factor 1 receptor |
| CSPG | chondroitin sulfate proteoglycan |
| Cu/Zn | copper/zinc |
| CYP46A1 | cytochrome P450, subfamily 46 (cholesterol 24-hydroxylase) |
| dl-NBP | dl-3-n-Butylphthalide |
| EAAT2 | excitatory amino acid transporter 2 |
| EGFR | epidermal growth factor receptor |
| EHMT | euchromatic histone-lysine N-methyltransferase |
| ERFR | Eagle Research Formulation of Ryanodex |
| FAAH | fatty acid amide hydrolase |
| GABAaR | γ-Aminobutyric acid type A receptor |
| GAIM | general amyloid interaction motif |
| GFAP | Glial fibrillary acidic protein |
| GSK3 | Glycogen synthase kinase 3 |
| h | human |
| HAT | histone acetyltransferase |
| HDAC | histone deacetylase |
| HER2 | human epidermal growth factor receptor 2 |
| HMGB1 | high mobility group box 1 |
| HMTM | hydromethylthionine mesylate |
| hrANXA1 | recombinant human annexin A1 |
| HSP | heat shock protein |
| LMTM | hydromethylthionine |
| LMTX | leucomethylthioninium salts |
| LNA | locked-nucleic-acid |
| LPS | lipopolysaccharide |
| MAGL | monoacylglycerol lipase |
| MAPK | mitogen-activated protein kinase |
| mGluR | metabotropic glutamate receptor |
| miRNA | microRNA |
| MT | microtubule |
| MTC | methylthioninium chloride |
| mTOR | mammalian target of rapamycin |
| MVA | modified vaccinia virus Ankara |
| NAM | negative allosteric modulator |
| NLRP3 | NOD-like receptor pyrin domain-containing protein 3 |
| NMDAR | N-methyl-D-aspartate receptor |
| NMNAT | nicotinamide mononucleotide adenylyltransferase |
| NPC | neural precursor cell |
| NRT | nucleoside reverse transcriptase |
| NSC | neural stem cell |
| nSMase2 | neutral sphingomyelinase 2 |
| O-GlcNAcase | O-Linked N-acetylglucosamine hydrolase |
| P2RX7 | P2X purinoceptor 7 |
| p75NTR | p75 neurotrophin receptor |
| PAC1R | pituitary adenylate cyclase–activating polypeptide type 1 receptor |
| palm11-PrRP31 | analog of prolactin releasing peptide 31, palmitoylated in position 11 |
| PAM | positive allosteric modulator |
| PD-1 | programmed cell death receptor 1 |
| PD-L1 Ab | programmed cell death receptor ligand |
| PDE | phosphodiesterase |
| PERK | RNA‐like endoplasmic reticulum kinase |
| PPARγ | peroxisome proliferator-activated receptor-γ |
| proNGF | the precursor of nerve growth factor |
| PROTAC | proteolysis targeting chimera |
| PTPN1 | protein tyrosine phosphatase non-receptor type 1 |
| R | receptor |
| RAGE | receptor for advanced glycation end products |
| Rbbp7 | RB Binding Protein 7, Chromatin Remodeling Factor |
| scFv | single chain variable fragment |
| siRNA | small interfering RNA |
| smyd3 | SET and MYND Domain Containing 3 |
| SSRI | selective serotonin reuptake inhibitor |
| STAT3 | signal transducer and activator of transcription 3 |
| SYK | spleen tyrosine kinase |
| TFEB | transcription factor EB |
| TfR | transferrin receptor |
| TfRMAb | transferrin receptor antibody |
| TIDMwt peptide | WT TLR2–interacting domain of MyD88 |
| TLR2 | Toll-like receptor 2 |
| TNAP | tissue-nonspecific alkaline phosphatase |
| TPC2 | two-pore channel 2 |
| TREM2 | triggering receptor expressed on myeloid cells 2 |
| VLPs | virus-like particles |
|  |  |
